# Supplementary material for: Hepatitis B, C and D virus infections and risk of hepatocellular carcinoma in Africa: A meta-analysis including sensitivity analyses for studies comparable for confounders
Source: PLoS One. 2022 Jan 21;17(1):e0262903. doi: 10.1371/journal.pone.0262903 (PMC8782350; doi:10.1371/journal.pone.0262903)
Supplement: S1 Table — (PDF) [file pone.0262903.s002.pdf]

S1 Table: Search strategy In Pubmed

Search		Search terms	Items found
#1	Outcomes	Hepatocellular Carcinoma OR HCC	
#2	Exposures	Hepatitis b OR Viral hepatitis b OR Hepatitis b virus OR HBV OR Hepatitis c OR Viral hepatitis c OR Hepatitis c virus OR HCV OR Hepatitis D OR Viral hepatitis D OR Hepatitis D virus OR HDV	
#3	Context	Africa* OR Algeria OR Angola OR Benin OR Botswana OR "Burkina Faso" OR Burundi OR Cameroon OR "Canary Islands" OR "Cape Verde" OR "Central African Republic" OR Chad OR Comoros OR Congo OR "Democratic Republic of Congo" OR Djibouti OR Egypt OR "Equatorial Guinea" OR Eritrea OR Ethiopia OR Gabon OR Gambia OR Ghana OR Guinea OR "Guinea Bissau" OR "Ivory Coast" OR "Cote d'Ivoire" OR Jamahiriya OR Kenya OR Lesotho OR Liberia OR Libya OR Madagascar OR Malawi OR Mali OR Mauritania OR Mauritius OR Mayotte OR Morocco OR Mozambique OR Namibia OR Niger OR Nigeria OR Principe OR Reunion OR Rwanda OR "Sao Tome" OR Senegal OR Seychelles OR "Sierra Leone" OR Somalia OR "South Africa" OR “South Sudan” OR "St Helena" OR Sudan OR Swaziland OR Tanzania OR Togo OR Tunisia OR Uganda OR "Western Sahara" OR Zaire OR Zambia OR Zimbabwe OR "Central Africa" OR "Central African" OR "West Africa" OR "West African" OR "Western Africa" OR "Western African" OR "East Africa" OR "East African" OR "Eastern Africa" OR "Eastern African" OR "North Africa" OR "North African" OR "Northern Africa" OR "Northern African" OR "South African" OR "Southern Africa" OR "Southern African" OR "sub Saharan Africa" OR "sub Saharan African" OR "sub Saharan Africa" OR "sub Saharan African”	
#4		#1 AND #2 AND #3	
#5		Limit #4 in English and French languages	
