# Supplementary material for: Hepatitis B, C and D virus infections and risk of hepatocellular carcinoma in Africa: A meta-analysis including sensitivity analyses for studies comparable for confounders
Source: PLoS One. 2022 Jan 21;17(1):e0262903. doi: 10.1371/journal.pone.0262903 (PMC8782350; doi:10.1371/journal.pone.0262903)
Supplement: S3 Table — (PDF) [file pone.0262903.s004.pdf]

S3 Table. Items for risk of bias assessment

**Newcastle – Ottawa Scale for case cohort studies**	**One star (1)/ No star (0)**
**Selection**	
1) Is the HCC case definition adequate?	**1**
2) Representativeness of the Cases?	**1**
3) Selection of Controls?	**1**
4) Definition of Controls?	**1**
**Comparability**	
1) Patients with and without HCC paired for age?	**1**
2) Patients with and without HCC paired for any additional factor?	**1**
**Exposure**	
1) Laboratory confirmation of hepatitis virus infection	**1**
2) Hepatitis virus infection status ascertain by secure record or structured interview blind to case/control status?	**1**
3) Same method of ascertainment hepatitis infection for cases and controls?	**1**
4) Non-Response rate similar for cases and controls?	**1**
Total score	**10**
**Interpretation of the two risk of bias tools**<br><br>• 7-10: Low risk of bias<br>• 4-6: Moderate risk of bias<br>• 0-3: High risk of bias	
