# Supplementary material for: Hepatitis B, C and D virus infections and risk of hepatocellular carcinoma in Africa: A meta-analysis including sensitivity analyses for studies comparable for confounders
Source: PLoS One. 2022 Jan 21;17(1):e0262903. doi: 10.1371/journal.pone.0262903 (PMC8782350; doi:10.1371/journal.pone.0262903)
Supplement: S4 Table — (PDF) [file pone.0262903.s005.pdf]

S4 Table. Main reasons of exclusion of eligible studies

N°	Author	Year	Title	Reason of exclusion
1	Abdel-Hamid	2007	Genetic diversity in hepatitis C virus in Egypt and possible association with hepatocellular carcinoma	Incorrect study design
2	Abdel-Wahab	2008	Aflatoxins as a risk factor for hepatocellular carcinoma in Egypt, Mansoura Gastroenterology Center study	No abstract and full text available
3	Ahmed	2020	Association of genetic polymorphism of BCL-2 (rs2279115) with susceptibility to HCV-related hepatocellular carcinoma	Incorrect study population
4	Amougou	2016	A prominent role of Hepatitis D Virus in liver cancers documented in Central Africa	Duplicate study
5	Andoulo	2014	Epidemiology of hepatitis C: related hepatocellular carcinoma in Cameroon	Incorrect study population
6	Aubry	1995	[Relationship between chronic liver diseases and hepatitis C in Burundi adults]	Incorrect study population
7	Badawi	1999	Risk factors for hepatocellular carcinoma in Egypt: the role of hepatitis-B viral infection and schistosomiasis	No abstract and full text available
8	Bastawi M	2004	Smoking as a risk factor for hepatocellular carcinoma [HCC] in patients with chronic hepatitis in Egypt	No abstract and full text available
9	Berry	1998	Association of hepatitis C virus & hepatitis B virus in chronic liver disease	No abstract and full text available
10	Bile	1993	Important role of hepatitis C virus infection as a cause of chronic liver disease in Somalia	Incorrect study population
11	Blumberg	1975	The relation of infection with the hepatitis B agent to primary hepatic carcinoma	Incorrect study design
12	Bowry	1981	A controlled study of hepatitis core antibody (HBcAb) in primary hepatocellular carcinoma and liver cirrhosis in Kenya	No abstract and full text available
13	Cenac	1995	Hepatitis B, C, and D virus infections in patients with chronic hepatitis, cirrhosis, and hepatocellular carcinoma: a comparative study in Niger	No abstract and full text available
14	Chen	1997	Epidemiological characteristics and risk factors of hepatocellular carcinoma	Incorrect study design
15	Cohen	1979	Liver cell dysplasia: association with hepatocellular carcinoma, cirrhosis and hepatitis B antigen carrier status	Incorrect study population
16	Council	2016	Association between Hepatitis C Virus Infection, p53 Phenotypes, and Gene Variants of Adenomatous Polyposis Coli in Hepatocellular Carcinomas	No viral hepatitis laboratory-confirmed data
17	Coursaget	1984	Hepatitis B virus serological markers in Africans with liver cirrhosis and hepatocellular carcinoma	No abstract and full text available
18	Coursaget	1980	Primary hepatocellular carcinoma in intertropical Africa: relationship between age and hepatitis B virus etiology.	Not possible to extract relevant data
19	Darwish	1997	Association of hepatitis C virus with liver cirrhosis and hepatocellular carcinoma compared with hepatitis B virus in Egyptian patients	Incorrect study population
20	Darwish	1993	Hepatitis C and B viruses, and their association with hepatocellular carcinoma in Egypt	Incorrect study population
21	Dazza	1993	Absence of a relationship between antibodies to hepatitis C virus and hepatocellular carcinoma in Mozambique	No abstract and full text available
22	Dourdourekas	1975	Hepatocellular carcinoma: relation to alcohol, HB-antigen and alpha-fetoprotein	No abstract and full text available
23	El-Tawdi	2016	Evaluation of Circulatory RNA-Based Biomarker Panel in Hepatocellular Carcinoma	Incorrect study population
24	Evans	1998	Geographic variation in viral load among hepatitis B carriers with differing risks of hepatocellular carcinoma	Incorrect study design
25	Getie	2020	The role of hepatitis C virus genotypes and core mutations in hepatocellular carcinoma in Cameroon	Duplicate study

26	Gilmore	1981	Clustering of hepatitis B virus infection and hepatocellular carcinoma in a family	Incorrect study design
27	Goldman	2007	Candidate markers for the detection of hepatocellular carcinoma in low-molecular weight fraction of serum	Duplicate study
28	Hamdy	2015	Assessment of serum adiponectin in Egyptian patients with HCV-related cirrhosis and hepatocellular carcinoma	Incorrect study population
29	Hifnawy	2004	The role of aflatoxin-contaminated food materials and HCV in developing hepatocellular carcinoma in Al-Sharkia Governorate, Egypt	No abstract and full text available
30	Howell	2016	PROLIFICA: a story of West African clinical and research collaborations to target hepatitis B-related hepatocellular carcinoma in West Africa	Incorrect study design
31	Hu	2014	Intent-to-Treat Analysis of Liver Transplant for Hepatocellular Carcinoma in the MELD Era: Impact of Hepatitis C and Advanced Status	Data outside of Africa
32	Isaacson	1979	Hepatitis B surface antigen and hepatocellular carcinoma in Southern Africa	Incorrect study population
33	Jaskiewicz	1995	Hepatocellular carcinoma in young patients: histology, cellular differentiation, HBV infection and oncoprotein p53	No abstract and full text available
34	Ka	1996	[Hepatitis B and C viruses in hepatocellular carcinoma in Senegal]	No abstract and full text available
35	Karayiannis	1984	Detection of serum HBV-DNA by molecular hybridisation Correlation with HBeAg/anti-HBe status, racial origin, liver histology and hepatocellular carcinoma	Incorrect study design
36	Kashala	1992	Hepatitis B virus, alpha-fetoprotein synthesis, and hepatocellular carcinoma in Zaire	Incorrect study design
37	Kew	1984	Does delta infection play a part in the pathogenesis of hepatitis B virus related hepatocellular carcinoma?	Incorrect study design
38	Kew	1988	Effect of age on the etiologic role of the hepatitis B virus in hepatocellular carcinoma in blacks	Incorrect study design
39	Kew	2006	Interaction between hepatitis B and C viruses in hepatocellular carcinogenesis	Incorrect study design
40	Kew	1979	Histocompatibility antigens in patients with hepatocellular carcinoma and their relationship to chronic hepatitis B virus infection in these patients	Incorrect study population
41	Kew	1985	Smoking as a risk factor in hepatocellular carcinoma A case-control study in southern African blacks	Incorrect study population
42	Kew	1997	The relative roles of hepatitis B and C viruses in the etiology of hepatocellular carcinoma in southern African blacks	Incorrect study population
43	Kew	1990	Contraceptive steroids as a risk factor for hepatocellular carcinoma: a case/control study in South African black women	No viral hepatitis laboratory-confirmed data
44	Kew	2003	Synergistic interaction between aflatoxin B1 and hepatitis B virus in hepatocarcinogenesis	No viral hepatitis laboratory-confirmed data
45	Khattab	2012	Association between metabolic abnormalities and hepatitis C-related hepatocellular carcinoma	Incorrect study population
46	Kirk	2004	The Gambia Liver Cancer Study: Infection with hepatitis B and C and the risk of hepatocellular carcinoma in West Africa	Duplicate study
47	Kirk	2005	Hepatocellular carcinoma and polymorphisms in carcinogen-metabolizing and DNA repair enzymes in a population with aflatoxin exposure and hepatitis B virus endemicity	Duplicate study
48	Lee	1993	Predominant etiologic association of hepatitis C virus with hepatocellular carcinoma compared with hepatitis B virus in elderly patients in a hepatitis B-endemic area	Data outside of Africa
49	Liu	2020	A Viral Exposure Signature Defines Early Onset of Hepatocellular Carcinoma	Data outside of Africa

50	Maher	2016	Serum microRNA panels as potential biomarkers for early detection of hepatocellular carcinoma on top of HCV infection	Incorrect study population
51	Mak	2020	Molecular characterization of hepatitis B virus isolated from Black South African cancer patients, with and without hepatocellular carcinoma	Incorrect study population
52	Matboli	2017	Clinical significance of miRNA-autophagy transcript expression in patients with hepatocellular carcinoma	Incorrect study population
53	M'Bengue	2015	A major shift of viral and nutritional risk factors affects the hepatocellular carcinoma risk among Ivorian patients: a preliminary report	Incorrect study population
54	Mendy	2010	Hepatitis B viral load and risk for liver cirrhosis and hepatocellular carcinoma in The Gambia, West Africa	Incorrect study design
55	Moyo	2016	The role of long non-coding RNAs in hepatitis B virus-related hepatocellular carcinoma	Incorrect study design
56	Nada	2005	The role of the tumor necrosis factor (TNF) - Fas L and HCV in the development of hepatocellular carcinoma	Incorrect study population
57	O'Callaghan	1997	Hepatitis B and C viruses and hepatocellular carcinoma	Incorrect study design
58	Ola	1994	Serum alphafoetoprotein, hepatitis B virus infection and primary hepatocellular carcinoma in Nigerians	Incorrect study population
59	Otedo	2018	Risk factors for liver Cancer in HIV endemic areas of Western Kenya	Incorrect study population
60	Otu	1987	Hepatocellular carcinoma, hepatic cirrhosis, and hepatitis B virus infection in Nigeria	Incorrect study population
61	Pati	1989	Association of hepatitis B virus with chronic liver diseases and hepatocellular carcinoma	No abstract and full text available
62	Perret	2002	[HBs Ag and antibodies to hepatitis C virus in complicated chronic liver disease in Gabon A case control study]	Incorrect study population
63	Peters	1994	Epidemiology of hepatocellular carcinoma Evaluation of viral and other risk factors in a low-endemic area for hepatitis B and C	Data outside of Africa
64	Reys	1977	The relationship between hepatitis B virus infection and hepatic cell carcinoma in Mozambique	No abstract and full text available
65	Ryu	2009	Lack of association between genotypes and subtypes of HCV and occurrence of hepatocellular carcinoma in Egypt	Incorrect study design
66	Sebti	1984	[Acute viral hepatitis and hepatitis B virus markers in chronic liver disease and primary cancer of the liver]	No abstract and full text available
67	Shire	2012	Viral hepatitis among Somali immigrants in Minnesota: association of hepatitis C with hepatocellular carcinoma	Data outside of Africa
68	Sjogren	1988	Hepatitis B virus infection and hepatocellular carcinoma: correlation between IgM antibody to hepatitis B core antigen, hepatitis B e antigen, and hepatitis B DNA	Incorrect study population
69	Smith	1973	Auto-antibodies in acute viral hepatitis, yellow fever, and hepatocellular carcinoma: clinical and experimental findings	Incorrect study population
70	Tang	2004	Hepatitis B viremia is associated with increased risk of hepatocellular carcinoma in chronic carriers	Incorrect study design
71	Tsega	1976	Hepatitis B antigen, alpha-fetoglobulins and primary hepatocellular carcinoma in Ethiopia	No abstract and full text available
72	Turner	2002	The role of aflatoxins and hepatitis viruses in the etiopathogenesis of hepatocellular carcinoma: A basis for primary prevention in Guinea-Conakry, West Africa	Incorrect study population
73	Tzonou	1991	Epidemiologic assessment of interactions of hepatitis-C virus with seromarkers of hepatitis-B and -D viruses, cirrhosis and tobacco smoking in hepatocellular carcinoma	Data outside of Africa
74	Vogel	1972	Hepatitis-associated antigen and antibody in hepatocellular carcinoma: results of a continuing study	No abstract and full text available
75	Wang	2020	Association between the Interleukin-10 -1082 G/A polymorphism and risk of hepatocellular carcinoma	Incorrect study design

76	Yates	1999	Hepatocellular carcinoma in Egyptians with and without a history of hepatitis B virus infection: association with hepatitis C virus (HCV) infection but not with (HCV) RNA level	Incorrect study design
77	Yuwen	1994	Hepatocellular carcinoma: lack of association with a unique hepatitis C virus nucleotide sequence	Incorrect study population
78	Zekri	2006	p53 mutation in HCV-genotype-4 associated hepatocellular carcinoma in Egyptian patients	No abstract and full text available
79	Zhu	2017	Genome-wide association study: new genetic insights into HBV/HCV-related hepatocellular carcinoma genomes	Incorrect study design
