# Supplementary material for: Hepatitis B, C and D virus infections and risk of hepatocellular carcinoma in Africa: A meta-analysis including sensitivity analyses for studies comparable for confounders
Source: PLoS One. 2022 Jan 21;17(1):e0262903. doi: 10.1371/journal.pone.0262903 (PMC8782350; doi:10.1371/journal.pone.0262903)
Supplement: S5 Table — (PDF) [file pone.0262903.s006.pdf]

S5 Table. Risk of bias assessment

Author, Year	Is the HCC case definition adequate?	Representativeness of the Cases?	Selection of Controls?	Definition of Controls?	Patients with and without HCC paired for age?	Patients with and without HCC paired for any additional factor?	Laboratory confirmation of hepatitis virus infection	Hepatitis virus infection status ascertain by secure record or structured interview blind to case/control status?	Same method of ascertainment hepatitis infection for cases and controls?	Non-Response rate similar for cases and controls?	Risk of bias
Amr, 2014	Yes	Yes	Yes	Yes	Yes	Yes	Yes	No	Yes	Yes	Low risk of bias
Amr, 2014	Yes	Yes	Yes	Yes	Yes	Yes	Yes	No	Yes	Yes	Low risk of bias
Bahri, 2011	Yes	Yes	Yes	Yes	Yes	Yes	Yes	No	Yes	Yes	Low risk of bias
Bahri, 2011	Yes	Yes	Yes	Yes	Yes	Yes	Yes	No	Yes	Yes	Low risk of bias
Brown, 1984	Yes	Yes	Yes	Yes	No	No	Yes	No	Yes	No	Moderate risk of bias
Cenac, 1987	Yes	Yes	No	No	No	No	Yes	No	Yes	No	Moderate risk of bias
Cenac, 1987	Yes	Yes	No	No	No	No	Yes	No	Yes	No	Moderate risk of bias
Cenac, 1987	Yes	Yes	Yes	Yes	No	No	Yes	No	Yes	No	Moderate risk of bias
Cenac, 1987	Yes	Yes	No	No	No	No	Yes	No	Yes	No	Moderate risk of bias
Cenac, 1987	Yes	Yes	No	No	No	No	Yes	No	Yes	No	Moderate risk of bias
Cenac, 1987	Yes	Yes	Yes	Yes	No	No	Yes	No	Yes	No	Moderate risk of bias
Chin'ombe, 2009	Yes	Yes	Yes	Yes	No	No	Yes	No	Yes	No	Moderate risk of bias
Chin'ombe, 2009	Yes	Yes	Yes	Yes	No	No	Yes	No	Yes	No	Moderate risk of bias
Chin'ombe, 2009	Yes	Yes	Yes	Yes	No	No	Yes	No	Yes	No	Moderate risk of bias
Coursaget, 1978	No	Yes	No	Yes	Yes	Yes	Yes	No	Yes	Unclear	Moderate risk of bias
Coursaget, 1978	No	Yes	No	Yes	Yes	Yes	Yes	No	Yes	Unclear	Moderate risk of bias
Coursaget, 1978	No	Yes	Yes	Yes	Yes	Yes	Yes	No	Yes	Unclear	Low risk of bias
Coursaget, 1978	No	Yes	Yes	Yes	Yes	Yes	Yes	No	Yes	Unclear	Low risk of bias
Coursaget, 1992	No	Yes	No	Yes	No	No	Yes	No	Yes	Unclear	Moderate risk of bias
Coursaget, 1992	No	Yes	No	Yes	No	No	Yes	No	Yes	Unclear	Moderate risk of bias
Coursaget, 1992	No	Yes	No	Yes	No	No	Yes	No	Yes	Unclear	Moderate risk of bias
Coursaget, 1992	No	Yes	No	Yes	No	No	Yes	No	Yes	Unclear	Moderate risk of bias
Cronberg, 1984	Yes	Yes	Yes	Yes	Yes	No	Yes	No	Yes	Unclear	Low risk of bias
Cronberg, 1984	Yes	Yes	Yes	Yes	Yes	No	Yes	No	Yes	Unclear	Low risk of bias
Cronberg, 1984	Yes	Yes	Yes	Yes	Yes	No	Yes	No	Yes	Unclear	Low risk of bias
Cronberg, 1984	Yes	Yes	Yes	Yes	Yes	No	Yes	No	Yes	Unclear	Low risk of bias
Dhifallah, 2020	No	Yes	No	Yes	Yes	Yes	Yes	No	Yes	Unclear	Moderate risk of bias
Dhifallah, 2020	No	Yes	No	Yes	Yes	Yes	Yes	No	Yes	Unclear	Moderate risk of bias
Dhifallah, 2020	No	Yes	No	Yes	Yes	Yes	Yes	No	Yes	Unclear	Moderate risk of bias
Dhifallah, 2020	No	Yes	No	Yes	Yes	Yes	Yes	No	Yes	Unclear	Moderate risk of bias
Dhifallah, 2020	No	Yes	No	Yes	Yes	Yes	Yes	No	Yes	Unclear	Moderate risk of bias
Ezzat, 2005	Yes	Yes	No	Yes	Yes	Yes	Yes	No	Yes	Unclear	Low risk of bias
Ezzat, 2005	Yes	Yes	No	Yes	Yes	Yes	Yes	No	Yes	Unclear	Low risk of bias
Ezzat, 2005	Yes	Yes	No	Yes	Yes	Yes	Yes	No	Yes	Unclear	Low risk of bias
Gouas, 2012	Yes	Yes	Yes	Yes	No	No	Yes	No	Yes	Unclear	Moderate risk of bias
Gouas, 2012	Yes	Yes	Yes	Yes	Yes	Yes	Yes	No	Yes	Unclear	Low risk of bias
Hassan, 2001	Yes	Yes	Yes	Yes	No	No	Yes	No	Yes	Unclear	Moderate risk of bias
Hassan, 2001	Yes	Yes	Yes	Yes	No	No	Yes	No	Yes	Unclear	Moderate risk of bias
Jaquet, 2018	Yes	Yes	Yes	Yes	Yes	Yes	Yes	No	Yes	Unclear	Low risk of bias
Jaquet, 2018	Yes	Yes	Yes	Yes	Yes	Yes	Yes	No	Yes	Unclear	Low risk of bias
Jaquet, 2018	Yes	Yes	Yes	Yes	Yes	Yes	Yes	No	Yes	Unclear	Low risk of bias
Jaquet, 2018	Yes	Yes	Yes	Yes	Yes	Yes	Yes	No	Yes	Unclear	Low risk of bias
Jaquet, 2018	Yes	Yes	Yes	Yes	Yes	Yes	Yes	No	Yes	Unclear	Low risk of bias
Jaquet, 2018	Yes	Yes	Yes	Yes	Yes	Yes	Yes	No	Yes	Unclear	Low risk of bias
Kew, 1979	Yes	Yes	Yes	Yes	Yes	Yes	Yes	No	Yes	Unclear	Low risk of bias
Kew, 1986	Yes	Yes	Yes	Yes	Yes	Yes	Yes	No	Yes	Unclear	Low risk of bias
Kew, 1990	Yes	Yes	Yes	Yes	Yes	Yes	Yes	No	Yes	Unclear	Low risk of bias
Kew, 1990	Yes	Yes	Yes	Yes	Yes	Yes	Yes	No	Yes	Unclear	Low risk of bias
Kew, 1990	Yes	Yes	Yes	Yes	Yes	Yes	Yes	No	Yes	Unclear	Low risk of bias
Kirk, 2005	Yes	Yes	Yes	Yes	No	No	Yes	No	Yes	No	Moderate risk of bias

Kirk, 2005	Yes	Yes	Yes	Yes	No	No	Yes	No	Yes	No	Moderate risk of bias
Kirk, 2005	Yes	Yes	Yes	Yes	No	No	Yes	No	Yes	No	Moderate risk of bias
Kirk, 2005	Yes	Yes	Yes	Yes	Yes	Yes	Yes	No	Yes	No	Low risk of bias
Kirk, 2005	Yes	Yes	Yes	Yes	Yes	Yes	Yes	No	Yes	No	Low risk of bias
Kirk, 2005	Yes	Yes	Yes	Yes	Yes	Yes	Yes	No	Yes	No	Low risk of bias
Larouzé, 1977	Yes	Yes	Yes	Yes	Yes	Yes	Yes	No	Yes	Unclear	Low risk of bias
Larouzé, 1977	Yes	Yes	Yes	Yes	Yes	Yes	Yes	No	Yes	Unclear	Low risk of bias
Lightfoot, 1997	Yes	Yes	No	Yes	Yes	Yes	Yes	No	Yes	No	Low risk of bias
Lightfoot, 1997	Yes	Yes	No	Yes	Yes	Yes	Yes	No	Yes	No	Low risk of bias
Mahale, 2019	Yes	Yes	Yes	Yes	Yes	Yes	Yes	No	Yes	No	Low risk of bias
Mahale, 2019	Yes	Yes	Yes	Yes	Yes	Yes	Yes	No	Yes	No	Low risk of bias
Mahale, 2019	Yes	Yes	Yes	Yes	Yes	Yes	Yes	No	Yes	No	Low risk of bias
Mahale, 2019	Yes	Yes	Yes	Yes	Yes	Yes	Yes	No	Yes	No	Low risk of bias
Mahale, 2019	Yes	Yes	Yes	Yes	Yes	Yes	Yes	No	Yes	No	Low risk of bias
Mahale, 2019	Yes	Yes	Yes	Yes	Yes	Yes	Yes	No	Yes	No	Low risk of bias
Mahale, 2019	Yes	Yes	Yes	Yes	Yes	Yes	Yes	No	Yes	No	Low risk of bias
Mahale, 2019	Yes	Yes	Yes	Yes	Yes	Yes	Yes	No	Yes	No	Low risk of bias
Mak, 2018	Yes	Yes	Yes	Yes	Yes	Yes	Yes	No	Yes	No	Low risk of bias
Mak, 2018	Yes	Yes	Yes	Yes	Yes	Yes	Yes	No	Yes	No	Low risk of bias
Mandishona, 1998	Yes	Yes	Yes	Yes	Yes	Yes	Yes	No	Yes	No	Low risk of bias
Mandishona, 1998	Yes	Yes	Yes	Yes	Yes	Yes	Yes	No	Yes	No	Low risk of bias
Marchio, 2018	Yes	Yes	Yes	Yes	Yes	Yes	Yes	No	Yes	No	Low risk of bias
Marchio, 2018	Yes	Yes	Yes	Yes	Yes	Yes	Yes	No	Yes	No	Low risk of bias
Marchio, 2018	Yes	Yes	Yes	Yes	Yes	Yes	Yes	No	Yes	No	Low risk of bias
Marchio, 2018	Yes	Yes	Yes	Yes	No	No	Yes	No	Yes	No	Moderate risk of bias
Marchio, 2018	Yes	Yes	Yes	Yes	No	No	Yes	No	Yes	No	Moderate risk of bias
Marchio, 2018	Yes	Yes	Yes	Yes	No	No	Yes	No	Yes	No	Moderate risk of bias
Marchio, 2018	Yes	Yes	Yes	Yes	No	No	Yes	No	Yes	No	Moderate risk of bias
Mboto, 2005	No	Yes	Yes	Yes	Yes	Yes	Yes	No	Yes	Unclear	Low risk of bias
Mboto, 2005	No	Yes	Yes	Yes	Yes	Yes	Yes	No	Yes	Unclear	Low risk of bias
Mets, 1993	No	Yes	Yes	Yes	No	Yes	Yes	No	Yes	Unclear	Moderate risk of bias
Mets, 1993	No	Yes	Yes	Yes	No	Yes	Yes	No	Yes	Unclear	Moderate risk of bias
Mets, 1993	No	Yes	Yes	Yes	No	No	Yes	No	Yes	Unclear	Moderate risk of bias
Mets, 1993	No	Yes	Yes	Yes	No	No	Yes	No	Yes	Unclear	Moderate risk of bias
Mohamed, 1992	Yes	Yes	Yes	Yes	Yes	Yes	Yes	No	Yes	Unclear	Low risk of bias
Montaser, 2007	Yes	Yes	Yes	Yes	No	No	Yes	No	Yes	Unclear	Moderate risk of bias
Montaser, 2007	Yes	Yes	Yes	Yes	No	No	Yes	No	Yes	Unclear	Moderate risk of bias
Montaser, 2007	Yes	Yes	Yes	Yes	Yes	Yes	Yes	No	Yes	Unclear	Low risk of bias
Montaser, 2007	Yes	Yes	Yes	Yes	Yes	Yes	Yes	No	Yes	Unclear	Low risk of bias
Nishioka, 1975	No	Yes	No	No	No	No	Yes	No	Yes	Unclear	High risk of bias
Nishioka, 1975	No	Yes	No	No	No	No	Yes	No	Yes	Unclear	High risk of bias
Ola, 2012	Yes	Yes	Yes	Yes	Yes	Yes	Yes	No	Yes	Unclear	Low risk of bias
Ola, 2012	Yes	Yes	Yes	Yes	Yes	Yes	Yes	No	Yes	Unclear	Low risk of bias
Ola, 2012	Yes	Yes	Yes	Yes	Yes	Yes	Yes	No	Yes	Unclear	Low risk of bias
Ola, 2012	Yes	Yes	Yes	Yes	Yes	Yes	Yes	No	Yes	Unclear	Low risk of bias
Olubuyide, 1997	Yes	Yes	Yes	Yes	Yes	Yes	Yes	No	Yes	Unclear	Low risk of bias
Olubuyide, 1997	Yes	Yes	Yes	Yes	Yes	Yes	Yes	No	Yes	Unclear	Low risk of bias
Olubuyide, 1997	Yes	Yes	Yes	Yes	Yes	Yes	Yes	No	Yes	Unclear	Low risk of bias
Omer, 2001	Yes	Yes	No	Yes	No	Yes	Yes	No	Yes	Yes	Low risk of bias
Omer, 2001	Yes	Yes	No	Yes	No	Yes	Yes	No	Yes	Yes	Low risk of bias

Schiefelbein, 2012	Yes	Yes	Yes	Yes	Yes	Yes	Yes	No	Yes	Unclear	Low risk of bias
Schiefelbein, 2012	Yes	Yes	Yes	Yes	Yes	Yes	Yes	No	Yes	Unclear	Low risk of bias
Schiefelbein, 2012	Yes	Yes	Yes	Yes	Yes	Yes	Yes	No	Yes	Unclear	Low risk of bias
Schiefelbein, 2012	Yes	Yes	Yes	Yes	Yes	Yes	Yes	No	Yes	Unclear	Low risk of bias
Skelton, 2000	Yes	Yes	Yes	Yes	Yes	Yes	Yes	No	Yes	Unclear	Low risk of bias
Skelton, 2000	Yes	Yes	Yes	Yes	Yes	Yes	Yes	No	Yes	Unclear	Low risk of bias
Soliman, 2010	Yes	Yes	Yes	Yes	Yes	Yes	Yes	No	Yes	Unclear	Low risk of bias
Soliman, 2010	Yes	Yes	Yes	Yes	Yes	Yes	Yes	No	Yes	Unclear	Low risk of bias
Soliman, 2010	Yes	Yes	Yes	Yes	Yes	Yes	Yes	No	Yes	Unclear	Low risk of bias
Tabor, 1977	Yes	Yes	Yes	Yes	No	Yes	Yes	No	Yes	Unclear	Low risk of bias
Tabor, 1977	Yes	Yes	Yes	Yes	No	Yes	Yes	No	Yes	Unclear	Low risk of bias
Tswana, 1992	Yes	Yes	Yes	Yes	Yes	Yes	Yes	No	Yes	Unclear	Low risk of bias
Tswana, 1992	Yes	Yes	Yes	Yes	Yes	Yes	Yes	No	Yes	Unclear	Low risk of bias
Tswana, 1992	Yes	Yes	Yes	Yes	Yes	Yes	Yes	No	Yes	Unclear	Low risk of bias
