# Supplementary material for: Hepatitis B, C and D virus infections and risk of hepatocellular carcinoma in Africa: A meta-analysis including sensitivity analyses for studies comparable for confounders
Source: PLoS One. 2022 Jan 21;17(1):e0262903. doi: 10.1371/journal.pone.0262903 (PMC8782350; doi:10.1371/journal.pone.0262903)
Supplement: S6 Table — (PDF) [file pone.0262903.s007.pdf]

S6 Table. Characteristics of included studies

**Characteristics**	**Overall (114)**	**HBV (62)**	**HCV (35)**	**HDV (12)**	**HBV/HCV (5)**
**Year of publication; range**	1975-2020	1975-2020	1990-2020	1987-2020	1990-2012
**Period of inclusion of participants; range**	1974-2015	1974-2015	1981-2015	1982-2014	1995-2009
**Study Design**					
Case control	114 (100.0)	62 (100.0)	35 (100.0)	12 (100.0)	5 (100.0)
**Sampling**					
Non probabilistic	105 (92.1)	58 (93.6)	31 (88.6)	12 (100.0)	4 (80.0)
Probabilistic	9 (7.9)	4 (6.5)	4 (11.4)		1 (20.0)
**Sampling method**					
Consecutive sampling	105 (92.1)	58 (93.6)	31 (88.6)	12 (100.0)	4 (80.0)
Simple random sampling	9 (7.9)	4 (6.5)	4 (11.4)		1 (20.0)
**Timing of viral hepatitis testing**					
Prospetively	56 (49.1)	33 (53.2)	15 (42.9)	6 (50.0)	2 (40.0)
Retrospectively	58 (50.9)	29 (46.8)	20 (57.1)	6 (50.0)	3 (60.0)
**Country**					
Gambia	19 (16.7)	10 (16.1)	5 (14.3)	4 (33.3)	
Egypt	18 (15.8)	7 (11.3)	9 (25.7)		2 (40.0)
South Africa	14 (12.3)	8 (12.9)	5 (14.3)		1 (20.0)
Senegal	13 (11.4)	11 (17.7)	2 (5.7)		
Cameroon; Central African Republic	7 (6.1)	3 (4.8)	2 (5.7)	2 (16.7)	
Nigeria	7 (6.1)	3 (4.8)	2 (5.7)	1 (8.3)	1 (20.0)
Niger	6 (5.3)	3 (4.8)		3 (25.0)	
Zimbabwe	6 (5.3)	3 (4.8)	2 (5.7)		1 (20.0)
Tunisia	5 (4.4)	2 (3.2)	1 (2.9)	2 (16.7)	
Rwanda	4 (3.5)	2 (3.2)	2 (5.7)		
Mali	3 (2.6)	2 (3.2)	1 (2.9)		
Côte d'Ivoire	2 (1.8)	1 (1.6)	1 (2.9)		
Kenya	2 (1.8)	2 (3.2)			
Sudan	2 (1.8)	1 (1.6)	1 (2.9)		
Togo	2 (1.8)	1 (1.6)	1 (2.9)		
Tunisia, Morocco, Algeria	2 (1.8)	1 (1.6)	1 (2.9)		
Uganda	1 (0.9)	1 (1.6)			
Zambia	1 (0.9)	1 (1.6)			
**UNSD Region**					
West Africa	52 (45.6)	31 (50.0)	12 (34.3)	8 (66.7)	1 (20.0)
Northern Africa	27 (23.7)	11 (17.7)	12 (34.3)	2 (16.7)	2 (40.0)
Southern Africa	14 (12.3)	9 (14.5)	5 (14.3)		1 (20.0)
Eastern Africa	14 (12.3)	8 (12.9)	4 (11.4)		1 (20.0)
Central Africa	7 (6.1)	3 (4.8)	2 (5.7)	2 (16.7)	
**Country income level**					
Lower-middle-income economies	56 (49.1)	31 (50.0)	18 (51.4)	3 (25.0)	4 (80.0)
Low-income economies	37 (32.5)	20 (32.3)	10 (28.6)	7 (58.3)	
Upper-middle-income economies	14 (12.3)	8 (12.9)	5 (14.3)		1 (20.0)
Low-income economies; Lower-middle income economies	7 (6.1)	3 (4.8)	2 (5.7)	2 (16.7)	
**Recrutment setting**					
Urban	30 (26.3)	18 (29.0)	9 (25.7)	3 (25.0)	
Urban/rural	28 (24.6)	11 (17.7)	10 (28.6)	6 (50.0)	1 (20.0)
Unclear/ Not reported	56 (49.1)	33 (53.2)	16 (45.7)	3 (25.0)	4 (80.0)

Characteristics	Overall (114)	HBV (62)	HCV (35)	HDV (12)	HBV/HCV (5)
**Setting**					
Hospital-based	89 (78.1)	47 (75.8)	30 (85.7)	8 (66.7)	4 (80.0)
Hospital/community based	10 (8.8)	7 (11.3)	3 (8.6)		
Community-based	6 (5.3)	3 (4.8)		3 (25.0)	
Unclear/ Not reported	9 (7.9)	5 (8.1)	2 (5.7)	1 (8.3)	1 (20.0)
**HCC diagnostic approach**					
Biochemically	70 (61.4)	37 (59.6)	21 (60.0)	10 (83.3)	2 (40.0)
Clinically	68 (59.6)	38 (61.2)	21 (60.0)	6 (50.0)	3 (60.0)
Histologically	70 (61.4)	35 (56.4)	20 (57.1)	10 (83.3)	5 (100)
Radiologically	70 (61.4)	32 (51.6)	24 (68.5)	10 (83.3)	4 (80.0)
Unclear/ Not reported	21 (18.4)	13 (21.0)	6 (17.1)	2 (16.6)	
**Number of HCC diagnosis approach**					
Multiple	79 (69.3)	41 (66.1)	24 (68.5)	10 (83.3)	4 (80.0)
Single	14 (12.2)	8 (12.9)	5 (14.2)		1 (20.0)
Unclear/ Not reported	21 (18.4)	13 (21.0)	6 (17.1)	2 (16.6)	
**Cases and controls pairing**					
Age	78 (68.4)	41 (66.1)	25 (71.4)	8 (66.7)	4 (80.0)
Gender	74 (64.9)	37 (59.7)	25 (71.4)	8 (66.7)	4 (80.0)
Recruitment hospital	14 (12.3)	7 (11.3)	5 (14.3)		
Place of residence	11 (9.7)	10 (16.1)	6 (17.1)		1 (20.0)
Ethnic origin	11 (9.7)	6 (9.7)	1 (2.9)		
Race	10 (8.8)	5 (8.1)	4 (11.4)		1 (20.0)
Geographical background (rural, urban or rural-urban)	5 (4.4)	2 (3.2)	2 (5.7)		1 (20.0)
Study site	4 (3.5)	3 (4.8)	1 (2.9)		
Type of ward (medical or surgical)	3 (2.6)	2 (3.2)	1 (2.9)		
**Controls inclusion criteria**					
Non-hepatic diseases	47 (41.2)	24 (38.7)	17 (48.6)	4 (33.3)	2 (40.0)
Healthy controls	40 (35.1)	22 (35.5)	12 (34.3)	3 (25.0)	3 (60.0)
Liver cirrhosis	17 (14.9)	10 (16.1)	4 (11.4)	3 (25.0)	
Other liver disorders	10 (8.8)	6 (9.7)	2 (5.7)	2 (16.7)	
**Viral hepatitis**					
VHB	62 (54.4)	62 (100.0)			
VHC	35 (30.7)		35 (100.0)		
VHD	12 (10.5)			12 (100.0)	
VHB/VHC	5 (4.4)				5 (100.0)
**Detection assay**					
Radioimmunoassay	26 (22.8)	19 (30.7)	3 (8.6)	3 (25.0)	1 (20.0)
Indirect ELISA	22 (19.3)	5 (8.1)	16 (45.7)	1 (8.3)	
Direct ELISA	17 (14.9)	9 (14.5)	5 (14.3)	3 (25.0)	
Enzyme immunoassay	16 (14.0)	12 (19.4)	3 (8.6)	1 (8.3)	
Rapid Diagnostic test	6 (5.3)	3 (4.8)	3 (8.6)		
Agar gel diffusion	4 (3.5)	4 (6.5)			
Chemiluminescent enzyme immunoassay	4 (3.5)	3 (4.8)	1 (2.9)		
RT-PCR	4 (3.5)		2 (5.7)	2 (16.7)	
PCR	3 (2.6)	3 (4.8)			
Immune adherence haemagglutination	2 (1.8)	1 (1.6)	1 (2.9)		
Indirect ELISA/Direct ELISA	2 (1.8)				2 (40.0)
Quantitative microarray antibody capture assay	2 (1.8)			2 (16.7)	
Reverse passive hemagglutination assay	2 (1.8)	2 (3.2)			
PCR/RT-PCR	1 (0.9)				1 (20.0)
Radioimmunoassay/Indirect ELISA	1 (0.9)				1 (20.0)
Unclear/Not reported	2 (1.8)	1 (1.6)	1 (2.9)		

Characteristics	Overall (114)	HBV (62)	HCV (35)	HDV (12)	HBV/HCV (5)
**Target detected**					
HBV: HBsAg (+)	10 (8.8)	10 (16.1)			
HBV: HBeAg (+)	48 (42.1)	48 (77.4)			
HBV: HBV DNA	4 (3.5)	4 (6.5)			
HCV: Anti-VHC	33 (29.0)		33 (94.3)		
HCV: HCV RNA	2 (1.8)		2 (5.7)		
HDV: Anti-delta (+)	8 (7.0)			8 (66.7)	
HDV: Ag Delta	2 (1.8)			2 (16.7)	
HDV: HDV RNA	2 (1.8)			2 (16.7)	
HBV/HCV: HBsAg (+)/HCV: Anti-VHC	4 (3.5)				4 (80.0)
HBV/HCV: HBV DNA/HCV: HCV RNA	1 (0.9)				1 (20.0)
