# Supplementary material for: Hepatitis B, C and D virus infections and risk of hepatocellular carcinoma in Africa: A meta-analysis including sensitivity analyses for studies comparable for confounders
Source: PLoS One. 2022 Jan 21;17(1):e0262903. doi: 10.1371/journal.pone.0262903 (PMC8782350; doi:10.1371/journal.pone.0262903)
Supplement: S7 Table — (PDF) [file pone.0262903.s008.pdf]

S7 Table. Individual characteristics of included studies

Author, Year	Study Design	Sampling	Sampling method	Timing of exposure collection	Country	UNSD Region	Country income level	Study period	Recrutment setting	Setting	HCC inclusion criteria	HCC diagnostic approach	Controls case definition	Viral hepatitis	Hepatitis detection assay	Target detected
Amr, 2014	Case control	Non probabilistic	Consecutive sampling	Retrospectively	Egypt	Northern Africa	Lower-middle-income economies	1999-2009	Urban	Hospital-based	Briefly, consecutive patients with presumed diagnosis of HCC were recruited from the National Cancer Institute of Cairo University from 1999 through 2009.	They were included in the study only if their liver malignancy was confirmed as primary by either 1) pathology or cytology evidence, 2) alpha-fetoprotein (AFP) levels> 1000 ng/ml or 3) AFP levels> 300 ng/ml along with evidence of single liver mass from an ultrasound or CT scan.	Controls were recruited from the Orthopedic Department of the nearby Kasr El Aini Medical Center of Cairo University, which receives patients from the same geographical areas as the cases. They were frequency-matched to cases by rural versus urban birthplace, gender, and 5-year age category.	VHC	Indirect ELISA	HCV: Anti-VHC
Amr, 2014	Case control	Non probabilistic	Consecutive sampling	Retrospectively	Egypt	Northern Africa	Lower-middle-income economies	1999-2009	Urban	Hospital-based	Briefly, consecutive patients with presumed diagnosis of HCC were recruited from the National Cancer Institute of Cairo University from 1999 through 2009.	They were included in the study only if their liver malignancy was confirmed as primary by either 1) pathology or cytology evidence, 2) alpha-fetoprotein (AFP) levels> 1000 ng/ml or 3) AFP levels> 300 ng/ml along with evidence of single liver mass from an ultrasound or CT scan.	Controls were recruited from the Orthopedic Department of the nearby Kasr El Aini Medical Center of Cairo University, which receives patients from the same geographical areas as the cases. They were frequency-matched to cases by rural versus urban birthplace, gender, and 5-year age category.	VHB	Enzyme immunoassay	HBV: HBsAg (+)
Bahri, 2011	Case control	Non probabilistic	Consecutive sampling	Prospetively	Tunisia, Morocco, Algeria	Northern Africa	Lower-middle-income economies	Jan/2002-Jan/2005	Unclear/ Not reported	Hospital-based	During the study period, 164 cases were recruited from the three countries and we attempted to recruit two matched controls.	Diagnosis of HCC was based on imaging showing the characteristic features of HCC and/or, when possible, histological assessment of tissues samples and serum alphafetoprotein levels.	Control subjects were patients admitted during the same period with no hepatic diseases. We conducted a case-control study matched by age (± 5 years) and gender in Tunisia, Morocco and Algeria from January 2002 to January 2005.	VHC	Indirect ELISA	HCV: Anti-VHC
Bahri, 2011	Case control	Non probabilistic	Consecutive sampling	Prospetively	Tunisia, Morocco, Algeria	Northern Africa	Lower-middle-income economies	Jan/2002-Jan/2005	Unclear/ Not reported	Hospital-based	During the study period, 164 cases were recruited from the three countries and we attempted to recruit two matched controls.	Diagnosis of HCC was based on imaging showing the characteristic features of HCC and/or, when possible, histological assessment of tissues samples and serum alphafetoprotein levels.	Control subjects were patients admitted during the same period with no hepatic diseases. We conducted a case-control study matched by age (± 5 years) and gender in Tunisia, Morocco and Algeria from January 2002 to January 2005.	VHB	Direct ELISA	HBV: HBsAg (+)
Brown, 1984	Case control	Non probabilistic	Consecutive sampling	Retrospectively	Gambia	West Africa	Low-income economies	Unclear/ Not reported	Unclear/ Not reported	Hospital-based	Serum samples were obtained from 36 (32 males and four females, mean age 46 3 years) HCC patients from the MRC Laboratories, Fajara, The Gambia and tested for HBsAg, anti-HBs and antibody to hepatitis B core antigen (anti-HBc).	The diagnosis ofHCC was made on histological or cytological evidence in 19 patients and on clinical criteria (abdominal pain and hepatomegaly) with a serum a-fetoprotein level greater than 1,000 pg/l in the remaining 17 patients.	Control sera from 70 (65 males and five females) healthy medical students were all negative for serological makers of hepatitis B (Table 1).	VHB	Radioimmunoassay	HBV: HBsAg (+)
Cenac, 1987	Case control	Non probabilistic	Consecutive sampling	Prospetively	Niger	West Africa	Low-income economies	Oct/1982-Jun/1985	Urban	Community-based	At least one of the following signs was necessary for inclusion in the study: hepatomegaly, jaundice, ascites, oesophageal varices, abdominal venous pattern, or splenomegaly.	HCC was diagnosed on 4 criteria: clinical (abdominal mass), echographical (large tumour with important vascularization), histological and serological (increased serum alpha-foetoprotein).	Unclear/ Not reported	VHB	Radioimmunoassay	HBV: HBsAg (+)
Cenac, 1987	Case control	Non probabilistic	Consecutive sampling	Prospetively	Niger	West Africa	Low-income economies	Oct/1982-Jun/1985	Urban	Community-based	At least one of the following signs was necessary for inclusion in the study: hepatomegaly, jaundice, ascites, oesophageal varices, abdominal venous pattern, or splenomegaly.	HCC was diagnosed on 4 criteria: clinical (abdominal mass), echographical (large tumour with important vascularization), histological and serological (increased serum alpha-foetoprotein).	Unclear/ Not reported	VHB	Radioimmunoassay	HBV: HBsAg (+)
Cenac, 1987	Case control	Non probabilistic	Consecutive sampling	Prospetively	Niger	West Africa	Low-income economies	Oct/1982-Jun/1985	Urban	Community-based	At least one of the following signs was necessary for inclusion in the study: hepatomegaly, jaundice, ascites, oesophageal varices, abdominal venous pattern, or splenomegaly.	HCC was diagnosed on 4 criteria: clinical (abdominal mass), echographical (large tumour with important vascularization), histological and serological (increased serum alpha-foetoprotein).	Cirrhosis diagnosis was based on clinical, biological and echographical signs: atrophic liver with increased density, dilated portal vein, small intra-hepatic vessels. When diagnosis was uncertam, confirmation was obtamed by histological findings (needle biopsy).	VHB	Radioimmunoassay	HBV: HBsAg (+)
Coursaget, 1992	Case control	Non probabilistic	Consecutive sampling	Prospetively	Senegal	West Africa	Lower-middle-income economies	Unclear/ Not reported	Unclear/ Not reported	Hospital/community based	Selected groups of 48 subjects suffering from liver cirrhosis and 49 subiects suffering from henatocellular carcinoma were investigated for fiCV serological markers.	Unclear/ Not reported	Selected groups of 48 subjects suffering from liver cirrhosis and 49 subiects suffering from henatocellular carcinoma were investigated for fiCV serological markers.	VHC	Indirect ELISA	HCV: Anti-VHC
Coursaget, 1992	Case control	Non probabilistic	Consecutive sampling	Prospetively	Senegal	West Africa	Lower-middle-income economies	Unclear/ Not reported	Unclear/ Not reported	Hospital/community based	Selected groups of 48 subjects suffering from liver cirrhosis and 49 subiects suffering from henatocellular carcinoma were investigated for fiCV serological markers.	Unclear/ Not reported	Selected groups of 48 subjects suffering from liver cirrhosis and 49 subiects suffering from henatocellular carcinoma were investigated for fiCV serological markers.	VHB	Radioimmunoassay	HBV: HBsAg (+)
Coursaget, 1992	Case control	Non probabilistic	Consecutive sampling	Prospetively	Senegal	West Africa	Lower-middle-income economies	Unclear/ Not reported	Unclear/ Not reported	Hospital/community based	Selected groups of 48 subjects suffering from liver cirrhosis and 49 subiects suffering from henatocellular carcinoma were investigated for fiCV serological markers.	Unclear/ Not reported	As a control group, 134 Senegalese adults-selected from the general population were also investigated for HCV infection.	VHC	Indirect ELISA	HCV: Anti-VHC
Coursaget, 1992	Case control	Non probabilistic	Consecutive sampling	Prospetively	Senegal	West Africa	Lower-middle-income economies	Unclear/ Not reported	Unclear/ Not reported	Hospital/community based	Selected groups of 48 subjects suffering from liver cirrhosis and 49 subiects suffering from henatocellular carcinoma were investigated for fiCV serological markers.	Unclear/ Not reported	As a control group, 134 Senegalese adults-selected from the general population were also investigated for HCV infection.	VHB	Radioimmunoassay	HBV: HBsAg (+)
Coursaget, 1978	Case control	Non probabilistic	Consecutive sampling	Prospetively	Senegal	West Africa	Lower-middle-income economies	Unclear/ Not reported	Urban	Hospital-based	Serum specimens of 76 cirrhosis patients and 103 PHC patients were obtained at the Le Dantec Hospital, Dakar, Senegal (Internal Medicine Department, M. Sankale).	Unclear/ Not reported	Serum specimens of 76 cirrhosis patients and 103 PHC patients were obtained at the Le Dantec Hospital, Dakar, Senegal (Internal Medicine Department, M. Sankale).	VHB	Radioimmunoassay	HBV: HBsAg (+)
Coursaget, 1978	Case control	Non probabilistic	Consecutive sampling	Prospetively	Senegal	West Africa	Lower-middle-income economies	Unclear/ Not reported	Urban	Hospital-based	Serum specimens of 76 cirrhosis patients and 103 PHC patients were obtained at the Le Dantec Hospital, Dakar, Senegal (Internal Medicine Department, M. Sankale).	Unclear/ Not reported	Serum specimens of 76 cirrhosis patients and 103 PHC patients were obtained at the Le Dantec Hospital, Dakar, Senegal (Internal Medicine Department, M. Sankale).	VHB	Agar gel diffusion	HBV: HBeAg (+)
Coursaget, 1978	Case control	Non probabilistic	Consecutive sampling	Prospetively	Senegal	West Africa	Lower-middle-income economies	Unclear/ Not reported	Urban	Hospital-based	Serum specimens of 76 cirrhosis patients and 103 PHC patients were obtained at the Le Dantec Hospital, Dakar, Senegal (Internal Medicine Department, M. Sankale).	Unclear/ Not reported	A control group was composed of serum samples from 100 blood donors of the National Blood Centre of Dakar (J. Linhard) matched for age, sex, and ethnic background (Table 1).	VHB	Radioimmunoassay	HBV: HBsAg (+)
Coursaget, 1978	Case control	Non probabilistic	Consecutive sampling	Prospetively	Senegal	West Africa	Lower-middle-income economies	Unclear/ Not reported	Urban	Hospital-based	Serum specimens of 76 cirrhosis patients and 103 PHC patients were obtained at the Le Dantec Hospital, Dakar, Senegal (Internal Medicine Department, M. Sankale).	Unclear/ Not reported	A control group was composed of serum samples from 100 blood donors of the National Blood Centre of Dakar (J. Linhard) matched for age, sex, and ethnic background (Table 1).	VHB	Agar gel diffusion	HBV: HBeAg (+)
Cronberg, 1984	Case control	Non probabilistic	Consecutive sampling	Prospetively	Senegal	West Africa	Lower-middle-income economies	Unclear/ Not reported	Unclear/ Not reported	Hospital-based	They had PHC, liver cirrhosis, chronic hepatitis or other liver diseases, or they were healthy controls.	A clinical diagnosis of PHC was made from the history of the patient and the results of physical clinical examination. Palpable tumors were often found. The diagnosis was occasionally confirmed by biopsy or at necropsy. A laboratory diagnosis of PHC was made by testing for alphafetoprotein (AFP) in serum from the patients, by a method that is described below. Out of I30 clinically diagnosed patients with PHC, 88 had AFP levels in serum above 100 pg/l (Table I). These patients were considered to have highly probable PHC. After clinical examination, 133 subjects were considered to be clinically healthy or to have liver disease other than PHC. Among these, 83 had serum levels of AFP less than 15 pg/l and were therefore considered not likely to have PHC. In the remaining 137 subjects the diagnosis was open for dispute, depending on the diagnostic criteria to be chosen.	The controls were of similar age to the patients and most of them were relatives who were considered healthy.	VHB	Radioimmunoassay	HBV: HBsAg (+)
Cronberg, 1984	Case control	Non probabilistic	Consecutive sampling	Prospetively	Senegal	West Africa	Lower-middle-income economies	Unclear/ Not reported	Unclear/ Not reported	Hospital-based	They had PHC, liver cirrhosis, chronic hepatitis or other liver diseases, or they were healthy controls.	A clinical diagnosis of PHC was made from the history of the patient and the results of physical clinical examination. Palpable tumors were often found. The diagnosis was occasionally confirmed by biopsy or at necropsy. A laboratory diagnosis of PHC was made by testing for alphafetoprotein (AFP) in serum from the patients, by a method that is described below. Out of I30 clinically diagnosed patients with PHC, 88 had AFP levels in serum above 100 pg/l (Table I). These patients were considered to have highly probable PHC. After clinical examination, 133 subjects were considered to be clinically healthy or to have liver disease other than PHC. Among these, 83 had serum levels of AFP less than 15 pg/l and were therefore considered not likely to have PHC. In the remaining 137 subjects the diagnosis was open for dispute, depending on the diagnostic criteria to be chosen.	The controls were of similar age to the patients and most of them were relatives who were considered healthy.	VHB	Agar gel diffusion	HBV: HBeAg (+)
Cronberg, 1984	Case control	Non probabilistic	Consecutive sampling	Prospetively	Senegal	West Africa	Lower-middle-income economies	Unclear/ Not reported	Unclear/ Not reported	Hospital-based	They had PHC, liver cirrhosis, chronic hepatitis or other liver diseases, or they were healthy controls.	A clinical diagnosis of PHC was made from the history of the patient and the results of physical clinical examination. Palpable tumors were often found. The diagnosis was occasionally confirmed by biopsy or at necropsy. A laboratory diagnosis of PHC was made by testing for alphafetoprotein (AFP) in serum from the patients, by a method that is described below. Out of I30 clinically diagnosed patients with PHC, 88 had AFP levels in serum above 100 pg/l (Table I). These patients were considered to have highly probable PHC. After clinical examination, 133 subjects were considered to be clinically healthy or to have liver disease other than PHC. Among these, 83 had serum levels of AFP less than 15 pg/l and were therefore considered not likely to have PHC. In the remaining 137 subjects the diagnosis was open for dispute, depending on the diagnostic criteria to be chosen.	The controls were of similar age to the patients and most of them were relatives who were considered healthy.	VHB	Radioimmunoassay	HBV: HBsAg (+)

Cronberg, 1984	Case control	Non probabilistic	Consecutive sampling	Prospetively	Senegal	West Africa	Lower-middle-income economies	Unclear/ Not reported	Unclear/ Not reported	Hospital-based	They had PHC, liver cirrhosis, chronic hepatitis or other liver diseases, or they were healthy controls.	A clinical diagnosis of PHC was made from the history of the patient and the results of physical clinical examination. Palpable tumors were often found. The diagnosis was occasionally confirmed by biopsy or at necropsy. A laboratory diagnosis of PHC was made by testing for alphafetoprotein (AFP) in serum from the patients, by a method that is described below. Out of I30 clinically diagnosed patients with PHC, 88 had AFP levels in serum above 100 pg/l (Table I). These patients were considered to have highly probable PHC. After clinical examination, 133 subjects were considered to be clinically healthy or to have liver disease other than PHC. Among these, 83 had serum levels of AFP less than 15 pg/l and were therefore considered not likely to have PHC. In the remaining 137 subjects the diagnosis was open for dispute, depending on the diagnostic criteria to be chosen.	The controls were of similar age to the patients and most of them were relatives who were considered healthy.	VHB	Agar gel diffusion	HBV: HBeAg (+)
Dhifallah, 2020	Case control	Non probabilistic	Consecutive sampling	Prospetively	Tunisia	Northern Africa	Lower-middle-income economies	Jan/2011- Dec/2013	Unclear/ Not reported	Hospital-based	During this period, 73 Tunisian HCC patients were prospectively recruited from two departments of gastroenterology at La Rabta Hospital (Tunis) and Tahar Maamouri Hospital (Nabeul, a coastal region in Tunisia) and one department of surgery at Mongi Slim Hospital (La Marsa, in the north of Tunis).	Unclear/ Not reported	The HCC group was compared with a control group constituted of 70 patients admitted during the same period to the same department for non-hepatic diseases. These two groups were matched by age (± 5 years) and gender.	VHD	Indirect ELISA	HDV: Anti-delta (+)
Dhifallah, 2020	Case control	Non probabilistic	Consecutive sampling	Prospetively	Tunisia	Northern Africa	Lower-middle-income economies	Jan/2011- Dec/2013	Unclear/ Not reported	Hospital-based	During this period, 73 Tunisian HCC patients were prospectively recruited from two departments of gastroenterology at La Rabta Hospital (Tunis) and Tahar Maamouri Hospital (Nabeul, a coastal region in Tunisia) and one department of surgery at Mongi Slim Hospital (La Marsa, in the north of Tunis).	Unclear/ Not reported	The HCC group was compared with a control group constituted of 70 patients admitted during the same period to the same department for non-hepatic diseases. These two groups were matched by age (± 5 years) and gender.	VHD	Direct ELISA	HDV: Ag Delta
Dhifallah, 2020	Case control	Non probabilistic	Consecutive sampling	Prospetively	Tunisia	Northern Africa	Lower-middle-income economies	Jan/2011- Dec/2013	Unclear/ Not reported	Hospital-based	During this period, 73 Tunisian HCC patients were prospectively recruited from two departments of gastroenterology at La Rabta Hospital (Tunis) and Tahar Maamouri Hospital (Nabeul, a coastal region in Tunisia) and one department of surgery at Mongi Slim Hospital (La Marsa, in the north of Tunis).	Unclear/ Not reported	The HCC group was compared with a control group constituted of 70 patients admitted during the same period to the same department for non-hepatic diseases. These two groups were matched by age (± 5 years) and gender.	VHC	Indirect ELISA	HCV: Anti-VHC
Dhifallah, 2020	Case control	Non probabilistic	Consecutive sampling	Prospetively	Tunisia	Northern Africa	Lower-middle-income economies	Jan/2011- Dec/2013	Unclear/ Not reported	Hospital-based	During this period, 73 Tunisian HCC patients were prospectively recruited from two departments of gastroenterology at La Rabta Hospital (Tunis) and Tahar Maamouri Hospital (Nabeul, a coastal region in Tunisia) and one department of surgery at Mongi Slim Hospital (La Marsa, in the north of Tunis).	Unclear/ Not reported	The HCC group was compared with a control group constituted of 70 patients admitted during the same period to the same department for non-hepatic diseases. These two groups were matched by age (± 5 years) and gender.	VHB	Direct ELISA	HBV: HBsAg (+)
Dhifallah, 2020	Case control	Non probabilistic	Consecutive sampling	Prospetively	Tunisia	Northern Africa	Lower-middle-income economies	Jan/2011- Dec/2013	Unclear/ Not reported	Hospital-based	During this period, 73 Tunisian HCC patients were prospectively recruited from two departments of gastroenterology at La Rabta Hospital (Tunis) and Tahar Maamouri Hospital (Nabeul, a coastal region in Tunisia) and one department of surgery at Mongi Slim Hospital (La Marsa, in the north of Tunis).	Unclear/ Not reported	The HCC group was compared with a control group constituted of 70 patients admitted during the same period to the same department for non-hepatic diseases. These two groups were matched by age (± 5 years) and gender.	VHB	Direct ELISA	HBV: HBeAg (+)
Ezzat, 2005	Case control	Non probabilistic	Consecutive sampling	Prospetively	Egypt	Northern Africa	Lower-middle-income economies	Unclear/ Not reported	Urban/rural	Hospital-based	Cases were recruited from the NCI of Cairo University, in downtown Cairo, Egypt. Cases and controls were eligible if they were above the age of 17, had a residence in Egypt for more than one year, were physically and mentally capable of understanding and completing the questionnaire, and were diagnosed within the last 6 months.	Cases were recruited with a provisional diagnosis of HCC; they were included in this study only if they had a definite or probable diagnosis of HCC after further investigation and documentation. First, haematoxin and eosin-stained sections were reviewed by the pathologist co-authors to confirm histopathologic diagnosis; if so it was confirmed as a definite case of HCC. Non malignant cases were dropped from this study. Cases without a pathological confirmation were investigated further by medical records review of cytology findings at the NCI, or by documentation of pathology or cytology results from an outside institution. Ultrasound or CT evidence for a mass in the liver, and the serum alphafetoprotein (AFP) level, were also considered as part of the records review. Based on all of these sources of evidence, cases were classified as having a definite diagnosis of HCC if there was documentation of: (a) pathology or cytology evidence of malignancy, or (b) AFP above 1000 ng/ml, or (c) AFP above 300 ng/ml plus evidence of single mass from ultrasound or CT. Cases were classified as having a probable diagnosis of HCC if they had an isolated finding of AFP above 300 ng/ml, or an isolated finding of a large mass on ultrasound or CT, or documentation of treatment for HCC. All other cases were dropped from the study.	Controls were recruited from orthopedic department of the Kasr El Aini Faculty of Medicine, Cairo University, in the same urban area as the NCI. Cases and controls were eligible if they were above the age of 17, had a residence in Egypt for more than one year, were physically and mentally capable of understanding and completing the questionnaire, and were diagnosed within the last 6 months. Controls were frequency- matched with cases as closely as possible on sex, age category (5 year age groups) and region of current residence (categorized as urban or rural based on the subject's report about their current residence). Also, the cases and controls were nearly of the same socioeconomic status since all subjects were seeking government-sponsored health care (see data in Table 2 comparing the education and marital status of cases and controls).	VHC	RT-PCR	HCV: HCV RNA
Ezzat, 2005	Case control	Non probabilistic	Consecutive sampling	Prospetively	Egypt	Northern Africa	Lower-middle-income economies	Unclear/ Not reported	Urban/rural	Hospital-based	Cases were recruited from the NCI of Cairo University, in downtown Cairo, Egypt. Cases and controls were eligible if they were above the age of 17, had a residence in Egypt for more than one year, were physically and mentally capable of understanding and completing the questionnaire, and were diagnosed within the last 6 months.	Cases were recruited with a provisional diagnosis of HCC; they were included in this study only if they had a definite or probable diagnosis of HCC after further investigation and documentation. First, haematoxin and eosin-stained sections were reviewed by the pathologist co-authors to confirm histopathologic diagnosis; if so it was confirmed as a definite case of HCC. Non malignant cases were dropped from this study. Cases without a pathological confirmation were investigated further by medical records review of cytology findings at the NCI, or by documentation of pathology or cytology results from an outside institution. Ultrasound or CT evidence for a mass in the liver, and the serum alphafetoprotein (AFP) level, were also considered as part of the records review. Based on all of these sources of evidence, cases were classified as having a definite diagnosis of HCC if there was documentation of: (a) pathology or cytology evidence of malignancy, or (b) AFP above 1000 ng/ml, or (c) AFP above 300 ng/ml plus evidence of single mass from ultrasound or CT. Cases were classified as having a probable diagnosis of HCC if they had an isolated finding of AFP above 300 ng/ml, or an isolated finding of a large mass on ultrasound or CT, or documentation of treatment for HCC. All other cases were dropped from the study.	Controls were recruited from orthopedic department of the Kasr El Aini Faculty of Medicine, Cairo University, in the same urban area as the NCI. Cases and controls were eligible if they were above the age of 17, had a residence in Egypt for more than one year, were physically and mentally capable of understanding and completing the questionnaire, and were diagnosed within the last 6 months. Controls were frequency- matched with cases as closely as possible on sex, age category (5 year age groups) and region of current residence (categorized as urban or rural based on the subject's report about their current residence). Also, the cases and controls were nearly of the same socioeconomic status since all subjects were seeking government-sponsored health care (see data in Table 2 comparing the education and marital status of cases and controls).	VHC	Indirect ELISA	HCV: Anti-VHC

Ezzat, 2005	Case control	Non probabilistic	Consecutive sampling	Prospetively	Egypt	Northern Africa	Lower-middle-income economies	Unclear/ Not reported	Urban/rural	Hospital-based	Cases were recruited from the NCI of Cairo University, in downtown Cairo, Egypt. Cases and controls were eligible if they were above the age of 17, had a residence in Egypt for more than one year, were physically and mentally capable of understanding and completing the questionnaire, and were diagnosed within the last 6 months.	Cases were recruited with a provisional diagnosis of HCC; they were included in this study only if they had a definite or probable diagnosis of HCC after further investigation and documentation. First, haematoxin and eosin-stained sections were reviewed by the pathologist co-authors to confirm histopathologic diagnosis; if so it was confirmed as a definite case of HCC. Non malignant cases were dropped from this study. Cases without a pathological confirmation were investigated further by medical records review of cytology findings at the NCI, or by documentation of pathology or cytology results from an outside institution. Ultrasound or CT evidence for a mass in the liver, and the serum alphafetoprotein (AFP) level, were also considered as part of the records review. Based on all of these sources of evidence, cases were classified as having a definite diagnosis of HCC if there was documentation of: (a) pathology or cytology evidence of malignancy, or (b) AFP above 1000 ng/ml, or (c) AFP above 300 ng/ml plus evidence of single mass from ultrasound or CT. Cases were classified as having a probable diagnosis of HCC if they had an isolated finding of AFP above 300 ng/ml, or an isolated finding of a large mass on ultrasound or CT, or documentation of treatment for HCC. All other cases were dropped from the study.	Controls were recruited from orthopedic department of the Kasr El Aini Faculty of Medicine, Cairo University, in the same urban area as the NCI. Cases and controls were eligible if they were above the age of 17, had a residence in Egypt for more than one year, were physically and mentally capable of understanding and completing the questionnaire, and were diagnosed within the last 6 months. Controls were frequency- matched with cases as closely as possible on sex, age category (5 year age groups) and region of current residence (categorized as urban or rural based on the subject's report about their current residence). Also, the cases and controls were nearly of the same socioeconomic status since all subjects were seeking government-sponsored health care (see data in Table 2 comparing the education and marital status of cases and controls).	VHB	Enzyme immunoassay	HBV: HBsAg (+)
Gouas, 2012	Case control	Non probabilistic	Consecutive sampling	Retrospectively	Gambia	West Africa	Low-income economies	Sept/1997-Jan/2001	Unclear/ Not reported	Hospital-based	Briefly, The Gambia Liver Cancer Study was a hospital-based case–control study in which incident cases of HCC and cirrhosis were recruited from three tertiary hospitals sites in The Gambia from September 1997 to January 2001.	The diagnosis of HCC was based on concordant clinical and ultrasonography findings and on serum levels of a-fetoprotein of ≥100 ng/ml.	Cirrhosis was diagnosed using clinical parameters and ultrasonography (27). A minority of the cases was confirmed by histopathology of liver biopsies (4).	VHB	PCR	HBV: HBV DNA
Gouas, 2012	Case control	Non probabilistic	Consecutive sampling	Retrospectively	Gambia	West Africa	Low-income economies	Sept/1997-Jan/2001	Unclear/ Not reported	Hospital-based	Briefly, The Gambia Liver Cancer Study was a hospital-based case–control study in which incident cases of HCC and cirrhosis were recruited from three tertiary hospitals sites in The Gambia from September 1997 to January 2001.	The diagnosis of HCC was based on concordant clinical and ultrasonography findings and on serum levels of a-fetoprotein of ≥100 ng/ml.	Hospital controls with no clinical evidence for liver disease were frequency matched by age (within 10 years), gender and study site.	VHB	PCR	HBV: HBV DNA
Hassan, 2001	Case control	Non probabilistic	Consecutive sampling	Prospetively	Egypt	Northern Africa	Lower-middle-income economies	Jul/1995-Jan/1996	Unclear/ Not reported	Hospital-based	The patients included in the study had HCC that was histologically confirmed by members of the University of Cairo National Cancer Institute pathology department, and the controls were healthy individuals who were visiting their hospitalized friends (nonrelatives) at the University of Cairo National Cancer Institute surgical oncology department.	The patients included in the study had HCC that was histologically confirmed by members of the University of Cairo National Cancer Institute pathology department, and the controls were healthy individuals who were visiting their hospitalized friends (nonrelatives) at the University of Cairo National Cancer Institute surgical oncology department.	The patients included in the study had HCC that was histologically confirmed by members of the University of Cairo National Cancer Institute pathology department, and the controls were healthy individuals who were visiting their hospitalized friends (nonrelatives) at the University of Cairo National Cancer Institute surgical oncology department. Matching was not performed. To be enrolled as a control subject, an individual had to have no personal history of liver tumors.	VHC	Radioimmunoassay	HCV: Anti-VHC
Hassan, 2001	Case control	Non probabilistic	Consecutive sampling	Prospetively	Egypt	Northern Africa	Lower-middle-income economies	Jul/1995-Jan/1996	Unclear/ Not reported	Hospital-based	The patients included in the study had HCC that was histologically confirmed by members of the University of Cairo National Cancer Institute pathology department, and the controls were healthy individuals who were visiting their hospitalized friends (nonrelatives) at the University of Cairo National Cancer Institute surgical oncology department.	The patients included in the study had HCC that was histologically confirmed by members of the University of Cairo National Cancer Institute pathology department, and the controls were healthy individuals who were visiting their hospitalized friends (nonrelatives) at the University of Cairo National Cancer Institute surgical oncology department.	The patients included in the study had HCC that was histologically confirmed by members of the University of Cairo National Cancer Institute pathology department, and the controls were healthy individuals who were visiting their hospitalized friends (nonrelatives) at the University of Cairo National Cancer Institute surgical oncology department. Matching was not performed. To be enrolled as a control subject, an individual had to have no personal history of liver tumors.	VHB	Direct ELISA	HBV: HBsAg (+)
Jaquet, 2018	Case control	Non probabilistic	Consecutive sampling	Retrospectively	Togo	West Africa	Low-income economies	2014-2015	Urban	Hospital-based	In the participating hospitals, clinical wards following adult patients seeking for care with a diagnosis of primary liver cancer were solicited to participate.	Suspected cases of HCC underwent a systematic standardized abdominal ultrasounds examination combined with a plasmatic α-fetoprotein (AFP) measurement for diagnostic confirmation. The diagnosis of HCC was defined as the combination of one or more space-occupying well-characterized tumours ≥2cm, suggestive of an HCC and a plasmatic AFP ≥400 ng/ml. In a few cases of abdominal ultrasound highly suggestive of HCC with a serum AFP <400 ng/ml, an abdominal CT Scan was requested for diagnostic confirmation. All cases of HCC were systematically reviewed and confirmed by a panel local gastroenterologists and cancer specialists.	A control group (two controls for one case of HCC) of hospitalized patients seeking care in the same teaching hospitals were recruited in wards not related to cancer or diseases reported in the literature as associated with infectious diseases including orthopaedic surgery, cardiovascular disease and endocrinology wards. All included controls needed to have a clear primary diagnosis that motivated their access to care. Controls were matched with cases on age (+/− two years), gender and participating referral hospital. Facing the high prevalence of HBV in West Africa, all control participants underwent a systematic standardized abdominal ultrasounds examination combined with a plasmatic AFP measurement to discard any sub-clinical HCC.	VHC	Rapid Diagnostic test	HCV: Anti-VHC
Jaquet, 2018	Case control	Non probabilistic	Consecutive sampling	Retrospectively	Togo	West Africa	Low-income economies	2014-2015	Urban	Hospital-based	In the participating hospitals, clinical wards following adult patients seeking for care with a diagnosis of primary liver cancer were solicited to participate.	Suspected cases of HCC underwent a systematic standardized abdominal ultrasounds examination combined with a plasmatic α-fetoprotein (AFP) measurement for diagnostic confirmation. The diagnosis of HCC was defined as the combination of one or more space-occupying well-characterized tumours ≥2cm, suggestive of an HCC and a plasmatic AFP ≥400 ng/ml. In a few cases of abdominal ultrasound highly suggestive of HCC with a serum AFP <400 ng/ml, an abdominal CT Scan was requested for diagnostic confirmation. All cases of HCC were systematically reviewed and confirmed by a panel local gastroenterologists and cancer specialists.	A control group (two controls for one case of HCC) of hospitalized patients seeking care in the same teaching hospitals were recruited in wards not related to cancer or diseases reported in the literature as associated with infectious diseases including orthopaedic surgery, cardiovascular disease and endocrinology wards. All included controls needed to have a clear primary diagnosis that motivated their access to care. Controls were matched with cases on age (+/− two years), gender and participating referral hospital. Facing the high prevalence of HBV in West Africa, all control participants underwent a systematic standardized abdominal ultrasounds examination combined with a plasmatic AFP measurement to discard any sub-clinical HCC.	VHB	Rapid Diagnostic test	HBV: HBsAg (+)
Jaquet, 2018	Case control	Non probabilistic	Consecutive sampling	Retrospectively	Côte d'Ivoire	West Africa	Lower-middle-income economies	2014-2015	Urban	Hospital-based	In the participating hospitals, clinical wards following adult patients seeking for care with a diagnosis of primary liver cancer were solicited to participate.	Suspected cases of HCC underwent a systematic standardized abdominal ultrasounds examination combined with a plasmatic α-fetoprotein (AFP) measurement for diagnostic confirmation. The diagnosis of HCC was defined as the combination of one or more space-occupying well-characterized tumours ≥2cm, suggestive of an HCC and a plasmatic AFP ≥400 ng/ml. In a few cases of abdominal ultrasound highly suggestive of HCC with a serum AFP <400 ng/ml, an abdominal CT Scan was requested for diagnostic confirmation. All cases of HCC were systematically reviewed and confirmed by a panel local gastroenterologists and cancer specialists.	A control group (two controls for one case of HCC) of hospitalized patients seeking care in the same teaching hospitals were recruited in wards not related to cancer or diseases reported in the literature as associated with infectious diseases including orthopaedic surgery, cardiovascular disease and endocrinology wards. All included controls needed to have a clear primary diagnosis that motivated their access to care. Controls were matched with cases on age (+/− two years), gender and participating referral hospital. Facing the high prevalence of HBV in West Africa, all control participants underwent a systematic standardized abdominal ultrasounds examination combined with a plasmatic AFP measurement to discard any sub-clinical HCC.	VHC	Rapid Diagnostic test	HCV: Anti-VHC
Jaquet, 2018	Case control	Non probabilistic	Consecutive sampling	Retrospectively	Côte d'Ivoire	West Africa	Lower-middle-income economies	2014-2015	Urban	Hospital-based	In the participating hospitals, clinical wards following adult patients seeking for care with a diagnosis of primary liver cancer were solicited to participate.	Suspected cases of HCC underwent a systematic standardized abdominal ultrasounds examination combined with a plasmatic α-fetoprotein (AFP) measurement for diagnostic confirmation. The diagnosis of HCC was defined as the combination of one or more space-occupying well-characterized tumours ≥2cm, suggestive of an HCC and a plasmatic AFP ≥400 ng/ml. In a few cases of abdominal ultrasound highly suggestive of HCC with a serum AFP <400 ng/ml, an abdominal CT Scan was requested for diagnostic confirmation. All cases of HCC were systematically reviewed and confirmed by a panel local gastroenterologists and cancer specialists.	A control group (two controls for one case of HCC) of hospitalized patients seeking care in the same teaching hospitals were recruited in wards not related to cancer or diseases reported in the literature as associated with infectious diseases including orthopaedic surgery, cardiovascular disease and endocrinology wards. All included controls needed to have a clear primary diagnosis that motivated their access to care. Controls were matched with cases on age (+/− two years), gender and participating referral hospital. Facing the high prevalence of HBV in West Africa, all control participants underwent a systematic standardized abdominal ultrasounds examination combined with a plasmatic AFP measurement to discard any sub-clinical HCC.	VHB	Rapid Diagnostic test	HBV: HBsAg (+)
Jaquet, 2018	Case control	Non probabilistic	Consecutive sampling	Retrospectively	Mali	West Africa	Low-income economies	2014-2015	Urban	Hospital-based	In the participating hospitals, clinical wards following adult patients seeking for care with a diagnosis of primary liver cancer were solicited to participate.	Suspected cases of HCC underwent a systematic standardized abdominal ultrasounds examination combined with a plasmatic α-fetoprotein (AFP) measurement for diagnostic confirmation. The diagnosis of HCC was defined as the combination of one or more space-occupying well-characterized tumours ≥2cm, suggestive of an HCC and a plasmatic AFP ≥400 ng/ml. In a few cases of abdominal ultrasound highly suggestive of HCC with a serum AFP <400 ng/ml, an abdominal CT Scan was requested for diagnostic confirmation. All cases of HCC were systematically reviewed and confirmed by a panel local gastroenterologists and cancer specialists.	A control group (two controls for one case of HCC) of hospitalized patients seeking care in the same teaching hospitals were recruited in wards not related to cancer or diseases reported in the literature as associated with infectious diseases including orthopaedic surgery, cardiovascular disease and endocrinology wards. All included controls needed to have a clear primary diagnosis that motivated their access to care. Controls were matched with cases on age (+/− two years), gender and participating referral hospital. Facing the high prevalence of HBV in West Africa, all control participants underwent a systematic standardized abdominal ultrasounds examination combined with a plasmatic AFP measurement to discard any sub-clinical HCC.	VHC	Rapid Diagnostic test	HCV: Anti-VHC

Jaquet, 2018	Case control	Non probabilistic	Consecutive sampling	Retrospectively	Mali	West Africa	Low-income economies	2014-2015	Urban	Hospital-based	In the participating hospitals, clinical wards following adult patients seeking for care with a diagnosis of primary liver cancer were solicited to participate.	Suspected cases of HCC underwent a systematic standardized abdominal ultrasounds examination combined with a plasmatic α-fetoprotein (AFP) measurement for diagnostic confirmation. The diagnosis of HCC was defined as the combination of one or more space-occupying well-characterized tumours ≥2cm, suggestive of an HCC and a plasmatic AFP ≥400 ng/ml. In a few cases of abdominal ultrasound highly suggestive of HCC with a serum AFP <400 ng/ml, an abdominal CT Scan was requested for diagnostic confirmation. All cases of HCC were systematically reviewed and confirmed by a panel local gastroenterologists and cancer specialists.	A control group (two controls for one case of HCC) of hospitalized patients seeking care in the same teaching hospitals were recruited in wards not related to cancer or diseases reported in the literature as associated with infectious diseases including orthopaedic surgery, cardiovascular disease and endocrinology wards. All included controls needed to have a clear primary diagnosis that motivated their access to care. Controls were matched with cases on age (+/− two years), gender and participating referral hospital. Facing the high prevalence of HBV in West Africa, all control participants underwent a systematic standardized abdominal ultrasounds examination combined with a plasmatic AFP measurement to discard any sub-clinical HCC.	VHB	Rapid Diagnostic test	HBV: HBsAg (+)
Kew, 1990	Case control	Non probabilistic	Consecutive sampling	Retrospectively	South Africa	Southern Africa	Upper-middle-income economies	Unclear/ Not reported	Urban/rural	Unclear/ Not reported	380 unselected southern African blacks (322 male, 58 female) with histologically proven HCC and 152 apparently healthy controls matched for race, sex, age, and rural/urban origin were studied.	380 unselected southern African blacks (322 male, 58 female) with histologically proven HCC and 152 apparently healthy controls matched for race, sex, age, and rural/urban origin were studied.	380 unselected southern African blacks (322 male, 58 female) with histologically proven HCC and 152 apparently healthy controls matched for race, sex, age, and rural/urban origin were studied.	VHC	Indirect ELISA	HCV: Anti-VHC
Kew, 1990	Case control	Non probabilistic	Consecutive sampling	Retrospectively	South Africa	Southern Africa	Upper-middle-income economies	Unclear/ Not reported	Urban/rural	Unclear/ Not reported	380 unselected southern African blacks (322 male, 58 female) with histologically proven HCC and 152 apparently healthy controls matched for race, sex, age, and rural/urban origin were studied.	380 unselected southern African blacks (322 male, 58 female) with histologically proven HCC and 152 apparently healthy controls matched for race, sex, age, and rural/urban origin were studied.	380 unselected southern African blacks (322 male, 58 female) with histologically proven HCC and 152 apparently healthy controls matched for race, sex, age, and rural/urban origin were studied.	VHB	Radioimmunoassay	HBV: HBsAg (+)
Kew, 1986	Case control	Non probabilistic	Consecutive sampling	Prospetively	South Africa	Southern Africa	Upper-middle-income economies	Unclear/ Not reported	Urban/rural	Hospital-based	The case-control study comprised 392 urban but otherwise unselected southern African blacks with histologically proved hepatocellular carcinoma and 392 matched controls. Serological markers of hepatitis B virus infection in 62 of the urban born blacks with hepatocellular carcinoma were compared with those of 62 matched rural born patients with hepatocellular carcinoma and 62 matched urban born subjects admitted to hospital for diseases other than hepatocellular carcinoma.	The case-control study comprised 392 urban but otherwise unselected southern African blacks with histologically proved hepatocellular carcinoma and 392 matched controls. Serological markers of hepatitis B virus infection in 62 of the urban born blacks with hepatocellular carcinoma were compared with those of 62 matched rural born patients with hepatocellular carcinoma and 62 matched urban born subjects admitted to hospital for diseases other than hepatocellular carcinoma.	Serological markers of hepatitis B virus infection in 62 of the urban born blacks with hepatocellular carcinoma were compared with those of 62 matched rural born patients with hepatocellular carcinoma and 62 matched urban born subjects admitted to hospital for diseases other than hepatocellular carcinoma. The urban born and rural born patients with hepatocellular carcinoma were matched for race, sex, age, and, when possible, tribe; the urban born patients with hepatocellular carcinoma and the controls were, in addition, matched for hospital and ward.	VHB	Radioimmunoassay	HBV: HBsAg (+)
Kew, 1979	Case control	Non probabilistic	Consecutive sampling	Retrospectively	South Africa	Southern Africa	Upper-middle-income economies	Unclear/ Not reported	Urban	Hospital-based	Sera from 289 southern African blacks with histologically proved HCC were tested for HBsAg and its specific antibody anti-HBs.	Sera from 289 southern African blacks with histologically proved HCC were tested for HBsAg and its specific antibody anti-HBs.	The control group for this part of the study was comprised of 213 apparently healthy age-matched, sex matched, and ethnically matched gold miners.	VHB	Radioimmunoassay	HBV: HBsAg (+)
Kirk, 2005	Case control	Non probabilistic	Consecutive sampling	Retrospectively	Gambia	West Africa	Low-income economies	Sep/1997-Jan/2001	Unclear/ Not reported	Hospital-based	Briefly, incident cases of HCC (n= 216) and cirrhosis (n= 121) were recruited from three tertiary hospitals sites in The Gambia from September 1997 to January 2001.	HCC cases were defined based on pathological examination in 54 (25.0%) and by a combination of ultrasound lesions compatible with HCC and an a-fetoprotein (AFP) level >100 ng/ml in 162 (75.0%).	Study participants diagnosed with cirrhosis met defined ultrasound criteria for cirrhosis (Lin et al., 1993) and had no focal lesions suggestive of HCC.	VHC	Indirect ELISA	HCV: Anti-VHC
Kirk, 2005	Case control	Non probabilistic	Consecutive sampling	Retrospectively	Gambia	West Africa	Low-income economies	Sep/1997-Jan/2001	Unclear/ Not reported	Hospital-based	Briefly, incident cases of HCC (n= 216) and cirrhosis (n= 121) were recruited from three tertiary hospitals sites in The Gambia from September 1997 to January 2001.	HCC cases were defined based on pathological examination in 54 (25.0%) and by a combination of ultrasound lesions compatible with HCC and an a-fetoprotein (AFP) level >100 ng/ml in 162 (75.0%).	Study participants diagnosed with cirrhosis met defined ultrasound criteria for cirrhosis (Lin et al., 1993) and had no focal lesions suggestive of HCC.	VHB	Enzyme immunoassay	HBV: HBsAg (+)
Kirk, 2005	Case control	Non probabilistic	Consecutive sampling	Retrospectively	Gambia	West Africa	Low-income economies	Sep/1997-Jan/2001	Unclear/ Not reported	Hospital-based	Briefly, incident cases of HCC (n= 216) and cirrhosis (n= 121) were recruited from three tertiary hospitals sites in The Gambia from September 1997 to January 2001.	HCC cases were defined based on pathological examination in 54 (25.0%) and by a combination of ultrasound lesions compatible with HCC and an a-fetoprotein (AFP) level >100 ng/ml in 162 (75.0%).	Study participants diagnosed with cirrhosis met defined ultrasound criteria for cirrhosis (Lin et al., 1993) and had no focal lesions suggestive of HCC.	VHB	Enzyme immunoassay	HBV: HBeAg (+)
Kirk, 2005	Case control	Non probabilistic	Consecutive sampling	Retrospectively	Gambia	West Africa	Low-income economies	Sep/1997-Jan/2001	Unclear/ Not reported	Hospital-based	Briefly, incident cases of HCC (n= 216) and cirrhosis (n= 121) were recruited from three tertiary hospitals sites in The Gambia from September 1997 to January 2001.	HCC cases were defined based on pathological examination in 54 (25.0%) and by a combination of ultrasound lesions compatible with HCC and an a-fetoprotein (AFP) level >100 ng/ml in 162 (75.0%).	Controls with no clinical evidence for liver disease (n¼408) were frequency matched by age (within 10 years), gender, and study site.	VHC	Indirect ELISA	HCV: Anti-VHC
Kirk, 2005	Case control	Non probabilistic	Consecutive sampling	Retrospectively	Gambia	West Africa	Low-income economies	Sep/1997-Jan/2001	Unclear/ Not reported	Hospital-based	Briefly, incident cases of HCC (n= 216) and cirrhosis (n= 121) were recruited from three tertiary hospitals sites in The Gambia from September 1997 to January 2001.	HCC cases were defined based on pathological examination in 54 (25.0%) and by a combination of ultrasound lesions compatible with HCC and an a-fetoprotein (AFP) level >100 ng/ml in 162 (75.0%).	Controls with no clinical evidence for liver disease (n¼408) were frequency matched by age (within 10 years), gender, and study site.	VHB	Enzyme immunoassay	HBV: HBsAg (+)
Kirk, 2005	Case control	Non probabilistic	Consecutive sampling	Retrospectively	Gambia	West Africa	Low-income economies	Sep/1997-Jan/2001	Unclear/ Not reported	Hospital-based	Briefly, incident cases of HCC (n= 216) and cirrhosis (n= 121) were recruited from three tertiary hospitals sites in The Gambia from September 1997 to January 2001.	HCC cases were defined based on pathological examination in 54 (25.0%) and by a combination of ultrasound lesions compatible with HCC and an a-fetoprotein (AFP) level >100 ng/ml in 162 (75.0%).	Controls with no clinical evidence for liver disease (n¼408) were frequency matched by age (within 10 years), gender, and study site.	VHB	Enzyme immunoassay	HBV: HBeAg (+)
Larouzé, 1977	Case control	Non probabilistic	Consecutive sampling	Prospetively	Mali	West Africa	Low-income economies	Jan/1974-Jul/1974	Unclear/ Not reported	Hospital/community based	Two groups of patients with PHC and one group with CLD were studied. The Mali PHC group included all patients identified between January and July 1974 (excluding May) by the same criteria used for the Senegal patients (No.=21). They were from the Point G and Gabriel Toure Hospitals in Bamako, Mali, and from the hospital of Kati, a village 5 km from Bamako.	The diagnosis of PHC was established on clinical grounds, including palpation of a tumor mass and/or an enlarged liver and by the presence of AFP as detected by immunodiffusion. In a few instances, the diagnosis was confirmed by histologic examination.	Forty apparently healthy individuals (2 controls for each case matched as in Senegal) were selected from villages near Bamako.	VHB	Radioimmunoassay	HBV: HBsAg (+)
Larouzé, 1977	Case control	Non probabilistic	Consecutive sampling	Prospetively	Senegal	West Africa	Lower-middle-income economies	May/1974-Sep/1974	Unclear/ Not reported	Hospital/community based	Two groups of patients with PHC and one group with CLD were studied. One PHC group, designated Senegal, was composed of 39 inpatients and outpatients from the Department of Internal Medicine of Le Dantec Hospital in Dakar, Senegal. These patients included all such patients seen by this department between May and September 1974. Patients with both PHC and tuberculosis were not included, but no other PHC patients were excluded.	The diagnosis of PHC was established on clinical grounds, including palpation of a tumor mass and/or an enlarged liver and by the presence of AFP as detected by immunodiffusion. In a few instances, the diagnosis was confirmed by histologic examination.	One or 2 controls (if suitable matches could be obtained) were selected for each PHC patient from healthy people living in villages near Dakar (No.= 11) and from inpatients and outpatients from Le Dantec Hospital (No.=47). None of the controls had diagnoses of hepatic or renal disease, cancer, leprosy, or tuberculosis.	VHB	Radioimmunoassay	HBV: HBsAg (+)
Lightfoot, 1997	Case control	Non probabilistic	Consecutive sampling	Retrospectively	South Africa	Southern Africa	Upper-middle-income economies	Unclear/ Not reported	Urban/rural	Hospital-based	One hundred and sixty seven unselected southern African blacks diagnosed to have HCC at four Johannesburg hospitals were entered into the study. (None of the patients with HCC suffered from acquired immunodeficiency syndrome or was human immunodeficiency virus–positive.)	HCC was diagnosed histologically or cytologically by fine needle aspiration, or on a combination of the clinical features, demonstration of one or more mass-lesions in the liver on hepatic imaging, and a serum a-fetoprotein concentration greater than 500 ng/mL (500 mg/L).	Each patient with HCC was matched for race, sex, age (within 2 years), geographical background (rural, urban, or rural-urban), and, whenever possible, tribal ethnicity with a patient from the same hospital and type of ward (medical or surgical) having a disease other than HCC and that was not known to be caused by either HGBV-C, HBV, or HCV. Specifically, patients suffering from acquired immunodeficiency syndrome or to be seropositive for the human immunodeficiency virus were excluded from the control pool because of possible confounding effects of this virus on the incidence and course of HGBV-C, HCV, and HBV infections.	VHC	Indirect ELISA	HCV: Anti-VHC
Lightfoot, 1997	Case control	Non probabilistic	Consecutive sampling	Retrospectively	South Africa	Southern Africa	Upper-middle-income economies	Unclear/ Not reported	Urban/rural	Hospital-based	One hundred and sixty seven unselected southern African blacks diagnosed to have HCC at four Johannesburg hospitals were entered into the study. (None of the patients with HCC suffered from acquired immunodeficiency syndrome or was human immunodeficiency virus–positive.)	HCC was diagnosed histologically or cytologically by fine needle aspiration, or on a combination of the clinical features, demonstration of one or more mass-lesions in the liver on hepatic imaging, and a serum a-fetoprotein concentration greater than 500 ng/mL (500 mg/L).	Each patient with HCC was matched for race, sex, age (within 2 years), geographical background (rural, urban, or rural-urban), and, whenever possible, tribal ethnicity with a patient from the same hospital and type of ward (medical or surgical) having a disease other than HCC and that was not known to be caused by either HGBV-C, HBV, or HCV. Specifically, patients suffering from acquired immunodeficiency syndrome or to be seropositive for the human immunodeficiency virus were excluded from the control pool because of possible confounding effects of this virus on the incidence and course of HGBV-C, HCV, and HBV infections.	VHB	Radioimmunoassay	HBV: HBsAg (+)
Mahale, 2019	Case control	Non probabilistic	Consecutive sampling	Retrospectively	Gambia	West Africa	Low-income economies	Sep/1997-Jan/2001	Urban/rural	Hospital-based	All patients with suspected liver disease underwent a standardized ultrasound examination.	Cases were patients with either (1) incident HCC confirmed by liver biopsy, or ultrasound showing one or more space-occupying lesions characteristic of HCC and a serum alpha-fetoprotein of ≥20 ng/ml, or (2) cirrhosis without HCC based on ultrasound findings that were consistent with cirrhosis in the absence of space-occupying lesions.	Cases were patients with either (1) incident HCC confirmed by liver biopsy, or ultrasound showing one or more space-occupying lesions characteristic of HCC and a serum alpha-fetoprotein of ≥20 ng/ml, or (2) cirrhosis without HCC based on ultrasound findings that were consistent with cirrhosis in the absence of space-occupying lesions.	VHD	Quantitative microarray antibody capture assay	HDV: Anti-delta (+)
Mahale, 2019	Case control	Non probabilistic	Consecutive sampling	Retrospectively	Gambia	West Africa	Low-income economies	Sep/1997-Jan/2001	Urban/rural	Hospital-based	All patients with suspected liver disease underwent a standardized ultrasound examination.	Cases were patients with either (1) incident HCC confirmed by liver biopsy, or ultrasound showing one or more space-occupying lesions characteristic of HCC and a serum alpha-fetoprotein of ≥20 ng/ml, or (2) cirrhosis without HCC based on ultrasound findings that were consistent with cirrhosis in the absence of space-occupying lesions.	Cases were patients with either (1) incident HCC confirmed by liver biopsy, or ultrasound showing one or more space-occupying lesions characteristic of HCC and a serum alpha-fetoprotein of ≥20 ng/ml, or (2) cirrhosis without HCC based on ultrasound findings that were consistent with cirrhosis in the absence of space-occupying lesions.	VHD	RT-PCR	HDV: HDV RNA
Mahale, 2019	Case control	Non probabilistic	Consecutive sampling	Retrospectively	Gambia	West Africa	Low-income economies	Sep/1997-Jan/2001	Urban/rural	Hospital-based	All patients with suspected liver disease underwent a standardized ultrasound examination.	Cases were patients with either (1) incident HCC confirmed by liver biopsy, or ultrasound showing one or more space-occupying lesions characteristic of HCC and a serum alpha-fetoprotein of ≥20 ng/ml, or (2) cirrhosis without HCC based on ultrasound findings that were consistent with cirrhosis in the absence of space-occupying lesions.	Control subjects with no clinical evidence of liver disease and normal alpha-foetoprotein levels (<5 ng/mL) were recruited from the general medical outpatient clinics of the same hospital sites and frequency matched to cases on age (10-year groupings) and gender.21	VHD	Quantitative microarray antibody capture assay	HDV: Anti-delta (+)
Mahale, 2019	Case control	Non probabilistic	Consecutive sampling	Retrospectively	Gambia	West Africa	Low-income economies	Sep/1997-Jan/2001	Urban/rural	Hospital-based	All patients with suspected liver disease underwent a standardized ultrasound examination.	Cases were patients with either (1) incident HCC confirmed by liver biopsy, or ultrasound showing one or more space-occupying lesions characteristic of HCC and a serum alpha-fetoprotein of ≥20 ng/ml, or (2) cirrhosis without HCC based on ultrasound findings that were consistent with cirrhosis in the absence of space-occupying lesions.	Control subjects with no clinical evidence of liver disease and normal alpha-foetoprotein levels (<5 ng/mL) were recruited from the general medical outpatient clinics of the same hospital sites and frequency matched to cases on age (10-year groupings) and gender.21	VHD	RT-PCR	HDV: HDV RNA

Mahale, 2019	Case control	Non probabilistic	Consecutive sampling	Retrospectively	Gambia	West Africa	Low-income economies	Sep/1997-Jan/2001	Urban/rural	Hospital-based	All patients with suspected liver disease underwent a standardized ultrasound examination.	Cases were patients with either (1) incident HCC confirmed by liver biopsy, or ultrasound showing one or more space-occupying lesions characteristic of HCC and a serum alpha-fetoprotein of ≥20 ng/ml, or (2) cirrhosis without HCC based on ultrasound findings that were consistent with cirrhosis in the absence of space-occupying lesions.	Cases were patients with either (1) incident HCC confirmed by liver biopsy, or ultrasound showing one or more space-occupying lesions characteristic of HCC and a serum alpha-fetoprotein of ≥20 ng/ml, or (2) cirrhosis without HCC based on ultrasound findings that were consistent with cirrhosis in the absence of space-occupying lesions.	VHC	Indirect ELISA	HCV: Anti-VHC
Mahale, 2019	Case control	Non probabilistic	Consecutive sampling	Retrospectively	Gambia	West Africa	Low-income economies	Sep/1997-Jan/2001	Urban/rural	Hospital-based	All patients with suspected liver disease underwent a standardized ultrasound examination.	Cases were patients with either (1) incident HCC confirmed by liver biopsy, or ultrasound showing one or more space-occupying lesions characteristic of HCC and a serum alpha-fetoprotein of ≥20 ng/ml, or (2) cirrhosis without HCC based on ultrasound findings that were consistent with cirrhosis in the absence of space-occupying lesions.	Cases were patients with either (1) incident HCC confirmed by liver biopsy, or ultrasound showing one or more space-occupying lesions characteristic of HCC and a serum alpha-fetoprotein of ≥20 ng/ml, or (2) cirrhosis without HCC based on ultrasound findings that were consistent with cirrhosis in the absence of space-occupying lesions.	VHB	Reverse passive hemagglutination assay	HBV: HBsAg (+)
Mahale, 2019	Case control	Non probabilistic	Consecutive sampling	Retrospectively	Gambia	West Africa	Low-income economies	Sep/1997-Jan/2001	Urban/rural	Hospital-based	All patients with suspected liver disease underwent a standardized ultrasound examination.	Cases were patients with either (1) incident HCC confirmed by liver biopsy, or ultrasound showing one or more space-occupying lesions characteristic of HCC and a serum alpha-fetoprotein of ≥20 ng/ml, or (2) cirrhosis without HCC based on ultrasound findings that were consistent with cirrhosis in the absence of space-occupying lesions.	Control subjects with no clinical evidence of liver disease and normal alpha-foetoprotein levels (<5 ng/mL) were recruited from the general medical outpatient clinics of the same hospital sites and frequency matched to cases on age (10-year groupings) and gender.21	VHC	Indirect ELISA	HCV: Anti-VHC
Mahale, 2019	Case control	Non probabilistic	Consecutive sampling	Retrospectively	Gambia	West Africa	Low-income economies	Sep/1997-Jan/2001	Urban/rural	Hospital-based	All patients with suspected liver disease underwent a standardized ultrasound examination.	Cases were patients with either (1) incident HCC confirmed by liver biopsy, or ultrasound showing one or more space-occupying lesions characteristic of HCC and a serum alpha-fetoprotein of ≥20 ng/ml, or (2) cirrhosis without HCC based on ultrasound findings that were consistent with cirrhosis in the absence of space-occupying lesions.	Control subjects with no clinical evidence of liver disease and normal alpha-foetoprotein levels (<5 ng/mL) were recruited from the general medical outpatient clinics of the same hospital sites and frequency matched to cases on age (10-year groupings) and gender.21	VHB	Reverse passive hemagglutination assay	HBV: HBsAg (+)
Mak, 2018	Case control	Non probabilistic	Consecutive sampling	Retrospectively	South Africa	Southern Africa	Upper-middle-income economies	Sep/2000-Dec/2012	Urban/rural	Hospital-based	Newly diagnosed with primary HCC (ICD-O: 8170/3 and ICD10: C22), with no prior history of any cancer, was enrolled from 1 September 2000 to 31 December 2012.	Fifty-one (34%) were confirmed by histology, 31 (20.7%) by cytology, 57 (38%) by elevated alpha fetoprotein levels (>450 ng/ml) and/or ultrasound (4) or CT scan (1), with the remaining cases (7.3%) confirmed as HCC by physicians.	For each HCC case three matched controls were randomly selected from the JCCCS database, matched by recruitment hospital, sex, and age at the time of interview (± 3 years).	VHC	Indirect ELISA	HCV: Anti-VHC
Mak, 2018	Case control	Non probabilistic	Consecutive sampling	Retrospectively	South Africa	Southern Africa	Upper-middle-income economies	Sep/2000-Dec/2012	Urban/rural	Hospital-based	Newly diagnosed with primary HCC (ICD-O: 8170/3 and ICD10: C22), with no prior history of any cancer, was enrolled from 1 September 2000 to 31 December 2012.	Fifty-one (34%) were confirmed by histology, 31 (20.7%) by cytology, 57 (38%) by elevated alpha fetoprotein levels (>450 ng/ml) and/or ultrasound (4) or CT scan (1), with the remaining cases (7.3%) confirmed as HCC by physicians.	For each HCC case three matched controls were randomly selected from the JCCCS database, matched by recruitment hospital, sex, and age at the time of interview (± 3 years).	VHB	Direct ELISA	HBV: HBV DNA
Mandishona, 1998	Case control	Non probabilistic	Consecutive sampling	Retrospectively	South Africa	Southern Africa	Upper-middle-income economies	Unclear/ Not reported	Urban/rural	Hospital-based	Twenty four consecutive patients from Shongwe and Themba Hospitals in the Mpumulanga Province of South Africa in whom HCC was suspected clinically underwent percutaneous liver biopsies for confirmation of the diagnosis and assessment of iron content of non-malignant hepatic tissue.	Twenty four consecutive patients from Shongwe and Themba Hospitals in the Mpumulanga Province of South Africa in whom HCC was suspected clinically underwent percutaneous liver biopsies for confirmation of the diagnosis and assessment of iron content of non-malignant hepatic tissue.	Two hospital controls without liver disease and matched for race, age, and sex were recruited for each patient. The 48 control subjects were predominantly hospitalized for trauma or acute or chronic infections. In addition, 51 first-degree relatives, 8 second degree relatives, and 16 unrelated family members of the index cases and living in proximity to the index cases were studied.	VHC	Indirect ELISA	HCV: Anti-VHC
Mandishona, 1998	Case control	Non probabilistic	Consecutive sampling	Retrospectively	South Africa	Southern Africa	Upper-middle-income economies	Unclear/ Not reported	Urban/rural	Hospital-based	Twenty four consecutive patients from Shongwe and Themba Hospitals in the Mpumulanga Province of South Africa in whom HCC was suspected clinically underwent percutaneous liver biopsies for confirmation of the diagnosis and assessment of iron content of non-malignant hepatic tissue.	Twenty four consecutive patients from Shongwe and Themba Hospitals in the Mpumulanga Province of South Africa in whom HCC was suspected clinically underwent percutaneous liver biopsies for confirmation of the diagnosis and assessment of iron content of non-malignant hepatic tissue.	Two hospital controls without liver disease and matched for race, age, and sex were recruited for each patient. The 48 control subjects were predominantly hospitalized for trauma or acute or chronic infections. In addition, 51 first-degree relatives, 8 second degree relatives, and 16 unrelated family members of the index cases and living in proximity to the index cases were studied.	VHB	Enzyme immunoassay	HBV: HBsAg (+)
Marchio, 2018	Case control	Non probabilistic	Consecutive sampling	Retrospectively	Cameroon; Central African Republic	Central Africa	Low-income economies; Lower-middle income economies	Feb/2013-Jan/2014	Urban/rural	Hospital-based	All participants were recruited in a single institution of Cameroun and Central African Republic. Cases were represented by patients affected with HCC consecutively enrolled in the Gastroenterology and Radiology Departments of the Central Hospital, Yaoundé, Cameroon, and in the Department of Hepato-Gastroenterology at the Hôpital de l'Amitié in Bangui, CAR, between February 2013 and January 2014.	The diagnosis of 195 HCC cases was based on suggestive clinical symptoms, presence of a liver mass at ultrasound, viral context, and, when possible, histology of tissues samples together with measurement of serum alpha-fetoprotein (AFP) levels.	In addition, a third group of subjects, patients without any liver disease, were taken to provide a baseline for the prevalence of TP53 mutations in Cameroonians and Central Africans. Cases were individually 1:1 paired–matched by sex and age (± 5 years) with control subjects consecutively selected and represented by patients with or without serological signs of chronic liver infection attending at the same period in the same medical departments. Patients with healthy liver (HL, n = 49) were recruited in the same medical departments where they were followed for a non-liver affection.	VHD	Direct ELISA	HDV: Anti-delta (+)
Marchio, 2018	Case control	Non probabilistic	Consecutive sampling	Retrospectively	Cameroon; Central African Republic	Central Africa	Low-income economies; Lower-middle income economies	Feb/2013-Jan/2014	Urban/rural	Hospital-based	All participants were recruited in a single institution of Cameroun and Central African Republic. Cases were represented by patients affected with HCC consecutively enrolled in the Gastroenterology and Radiology Departments of the Central Hospital, Yaoundé, Cameroon, and in the Department of Hepato-Gastroenterology at the Hôpital de l'Amitié in Bangui, CAR, between February 2013 and January 2014.	The diagnosis of 195 HCC cases was based on suggestive clinical symptoms, presence of a liver mass at ultrasound, viral context, and, when possible, histology of tissues samples together with measurement of serum alpha-fetoprotein (AFP) levels.	In addition, a third group of subjects, patients without any liver disease, were taken to provide a baseline for the prevalence of TP53 mutations in Cameroonians and Central Africans. Cases were individually 1:1 paired–matched by sex and age (± 5 years) with control subjects consecutively selected and represented by patients with or without serological signs of chronic liver infection attending at the same period in the same medical departments. Patients with healthy liver (HL, n = 49) were recruited in the same medical departments where they were followed for a non-liver affection.	VHC	Enzyme immunoassay	HCV: Anti-VHC
Marchio, 2018	Case control	Non probabilistic	Consecutive sampling	Retrospectively	Cameroon; Central African Republic	Central Africa	Low-income economies; Lower-middle income economies	Feb/2013-Jan/2014	Urban/rural	Hospital-based	All participants were recruited in a single institution of Cameroun and Central African Republic. Cases were represented by patients affected with HCC consecutively enrolled in the Gastroenterology and Radiology Departments of the Central Hospital, Yaoundé, Cameroon, and in the Department of Hepato-Gastroenterology at the Hôpital de l'Amitié in Bangui, CAR, between February 2013 and January 2014.	The diagnosis of 195 HCC cases was based on suggestive clinical symptoms, presence of a liver mass at ultrasound, viral context, and, when possible, histology of tissues samples together with measurement of serum alpha-fetoprotein (AFP) levels.	In addition, a third group of subjects, patients without any liver disease, were taken to provide a baseline for the prevalence of TP53 mutations in Cameroonians and Central Africans. Cases were individually 1:1 paired–matched by sex and age (± 5 years) with control subjects consecutively selected and represented by patients with or without serological signs of chronic liver infection attending at the same period in the same medical departments. Patients with healthy liver (HL, n = 49) were recruited in the same medical departments where they were followed for a non-liver affection.	VHB	Indirect ELISA	HBV: HBsAg (+)
Marchio, 2018	Case control	Non probabilistic	Consecutive sampling	Retrospectively	Cameroon; Central African Republic	Central Africa	Low-income economies; Lower-middle income economies	Feb/2013-Jan/2014	Urban/rural	Hospital-based	All participants were recruited in a single institution of Cameroun and Central African Republic. Cases were represented by patients affected with HCC consecutively enrolled in the Gastroenterology and Radiology Departments of the Central Hospital, Yaoundé, Cameroon, and in the Department of Hepato-Gastroenterology at the Hôpital de l'Amitié in Bangui, CAR, between February 2013 and January 2014.	The diagnosis of 195 HCC cases was based on suggestive clinical symptoms, presence of a liver mass at ultrasound, viral context, and, when possible, histology of tissues samples together with measurement of serum alpha-fetoprotein (AFP) levels.	The study was designed as a case-control study comparing patients with a hepatocellular carcinoma (HCC) considered as cases, with patients with chronic liver diseases (CLD) but without liver cancer. Inclusion criteria for control subjects with a known chronic liver disease (CLD, n = 263) were the absence of liver mass at ultrasound and a normal level of serum AFP (< 10 ng/mL).	VHC	Direct ELISA	HCV: Anti-VHC
Marchio, 2018	Case control	Non probabilistic	Consecutive sampling	Retrospectively	Cameroon; Central African Republic	Central Africa	Low-income economies; Lower-middle income economies	Feb/2013-Jan/2014	Urban/rural	Hospital-based	All participants were recruited in a single institution of Cameroun and Central African Republic. Cases were represented by patients affected with HCC consecutively enrolled in the Gastroenterology and Radiology Departments of the Central Hospital, Yaoundé, Cameroon, and in the Department of Hepato-Gastroenterology at the Hôpital de l'Amitié in Bangui, CAR, between February 2013 and January 2014.	The diagnosis of 195 HCC cases was based on suggestive clinical symptoms, presence of a liver mass at ultrasound, viral context, and, when possible, histology of tissues samples together with measurement of serum alpha-fetoprotein (AFP) levels.	The study was designed as a case-control study comparing patients with a hepatocellular carcinoma (HCC) considered as cases, with patients with chronic liver diseases (CLD) but without liver cancer. Inclusion criteria for control subjects with a known chronic liver disease (CLD, n = 263) were the absence of liver mass at ultrasound and a normal level of serum AFP (< 10 ng/mL).	VHD	Enzyme immunoassay	HDV: Anti-delta (+)
Marchio, 2018	Case control	Non probabilistic	Consecutive sampling	Retrospectively	Cameroon; Central African Republic	Central Africa	Low-income economies; Lower-middle income economies	Feb/2013-Jan/2014	Urban/rural	Hospital-based	All participants were recruited in a single institution of Cameroun and Central African Republic. Cases were represented by patients affected with HCC consecutively enrolled in the Gastroenterology and Radiology Departments of the Central Hospital, Yaoundé, Cameroon, and in the Department of Hepato-Gastroenterology at the Hôpital de l'Amitié in Bangui, CAR, between February 2013 and January 2014.	The diagnosis of 195 HCC cases was based on suggestive clinical symptoms, presence of a liver mass at ultrasound, viral context, and, when possible, histology of tissues samples together with measurement of serum alpha-fetoprotein (AFP) levels.	The study was designed as a case-control study comparing patients with a hepatocellular carcinoma (HCC) considered as cases, with patients with chronic liver diseases (CLD) but without liver cancer. Inclusion criteria for control subjects with a known chronic liver disease (CLD, n = 263) were the absence of liver mass at ultrasound and a normal level of serum AFP (< 10 ng/mL).	VHB	Indirect ELISA	HBV: HBsAg (+)
Marchio, 2018	Case control	Non probabilistic	Consecutive sampling	Retrospectively	Cameroon; Central African Republic	Central Africa	Low-income economies; Lower-middle income economies	Feb/2013-Jan/2014	Urban/rural	Hospital-based	All participants were recruited in a single institution of Cameroun and Central African Republic. Cases were represented by patients affected with HCC consecutively enrolled in the Gastroenterology and Radiology Departments of the Central Hospital, Yaoundé, Cameroon, and in the Department of Hepato-Gastroenterology at the Hôpital de l'Amitié in Bangui, CAR, between February 2013 and January 2014.	The diagnosis of 195 HCC cases was based on suggestive clinical symptoms, presence of a liver mass at ultrasound, viral context, and, when possible, histology of tissues samples together with measurement of serum alpha-fetoprotein (AFP) levels.	The study was designed as a case-control study comparing patients with a hepatocellular carcinoma (HCC) considered as cases, with patients with chronic liver diseases (CLD) but without liver cancer. Inclusion criteria for control subjects with a known chronic liver disease (CLD, n = 263) were the absence of liver mass at ultrasound and a normal level of serum AFP (< 10 ng/mL).	VHB	Direct ELISA	HBV: HBeAg (+)
Mboto, 2005	Case control	Non probabilistic	Consecutive sampling	Prospetively	Gambia	West Africa	Low-income economies	Jul/2002-Dec/2002	Urban	Hospital-based	The study population consisted of a total of 13 HCC patients seen consecutively at the Royal Victoria Teaching Hospital (RVTH), Banjul between the months of July to December 2002. The patients were aged 32 years to 76 years and were made up of 11 men and 2 women.	Unclear/ Not reported	Each patient was matched by three persons on the basis of age and sex. The primary choice of control group persons were blood donors, however due to the lack of female blood donors, the two female HCC patients were matched with two women attending antenatal clinic in their first trimester of pregnancy, and four other female patients with history of malaria. In all a total of thirty-nine healthy controls made up of 33 blood donors and 6 women were enrolled for the study.	VHC	Enzyme immunoassay	HCV: Anti-VHC
Mboto, 2005	Case control	Non probabilistic	Consecutive sampling	Prospetively	Gambia	West Africa	Low-income economies	Jul/2002-Dec/2002	Urban	Hospital-based	The study population consisted of a total of 13 HCC patients seen consecutively at the Royal Victoria Teaching Hospital (RVTH), Banjul between the months of July to December 2002. The patients were aged 32 years to 76 years and were made up of 11 men and 2 women.	Unclear/ Not reported	Each patient was matched by three persons on the basis of age and sex. The primary choice of control group persons were blood donors, however due to the lack of female blood donors, the two female HCC patients were matched with two women attending antenatal clinic in their first trimester of pregnancy, and four other female patients with history of malaria. In all a total of thirty-nine healthy controls made up of 33 blood donors and 6 women were enrolled for the study.	VHB	Enzyme immunoassay	HBV: HBsAg (+)

Mets, 1993	Case control	Non probabilistic	Consecutive sampling	Prospetively	Rwanda	Eastern Africa	Low-income economies	1981-1987	Urban	Hospital-based	All patients with suspicion of LC or HCC were included and were examined by ultrasound (Kranzbuhler real time, 3.5 MHz). Biopsy specimens were handled routinely, and diagnoses were made without knowledge of any serologic results. Only patients with histologically proven diagnoses of LC and HCC were considered for further study.	Unclear/ Not reported	Persons volunteering as blood donors in 1985 were used as controls; all volunteers came from the same region as the patients and had similar life styles.	VHC	Enzyme immunoassay	HCV: Anti-VHC
Mets, 1993	Case control	Non probabilistic	Consecutive sampling	Prospetively	Rwanda	Eastern Africa	Low-income economies	1981-1987	Urban	Hospital-based	All patients with suspicion of LC or HCC were included and were examined by ultrasound (Kranzbuhler real time, 3.5 MHz). Biopsy specimens were handled routinely, and diagnoses were made without knowledge of any serologic results. Only patients with histologically proven diagnoses of LC and HCC were considered for further study.	Unclear/ Not reported	Persons volunteering as blood donors in 1985 were used as controls; all volunteers came from the same region as the patients and had similar life styles.	VHB	Enzyme immunoassay	HBV: HBsAg (+)
Mets, 1993	Case control	Non probabilistic	Consecutive sampling	Prospetively	Rwanda	Eastern Africa	Low-income economies	1981-1987	Urban	Hospital-based	All patients with suspicion of LC or HCC were included and were examined by ultrasound (Kranzbuhler real time, 3.5 MHz). Biopsy specimens were handled routinely, and diagnoses were made without knowledge of any serologic results. Only patients with histologically proven diagnoses of LC and HCC were considered for further study.	Unclear/ Not reported	Signs of alcoholic hepatitis (mainly polymorphonuclear infiltration and, when present, Mallory bodies) and the presence of pericellular fibrosis were considered to be indicative of alcoholic cirrhosis, whereas the presence of a mononuclear infiltrate and piecemeal necrosis and the absence of pericellular fibrosis were considered to be indicative of postnecrotic cirrhosis. If insufficient histologic evidence was present to enable classification, the LC was considered indeterminate.	VHC	Radioimmunoassay	HCV: Anti-VHC
Mets, 1993	Case control	Non probabilistic	Consecutive sampling	Prospetively	Rwanda	Eastern Africa	Low-income economies	1981-1987	Urban	Hospital-based	All patients with suspicion of LC or HCC were included and were examined by ultrasound (Kranzbuhler real time, 3.5 MHz). Biopsy specimens were handled routinely, and diagnoses were made without knowledge of any serologic results. Only patients with histologically proven diagnoses of LC and HCC were considered for further study.	Unclear/ Not reported	Signs of alcoholic hepatitis (mainly polymorphonuclear infiltration and, when present, Mallory bodies) and the presence of pericellular fibrosis were considered to be indicative of alcoholic cirrhosis, whereas the presence of a mononuclear infiltrate and piecemeal necrosis and the absence of pericellular fibrosis were considered to be indicative of postnecrotic cirrhosis. If insufficient histologic evidence was present to enable classification, the LC was considered indeterminate.	VHB	Chemiluminescent enzyme immunoassay	HBV: HBsAg (+)
Mohamed, 1992	Case control	Non probabilistic	Consecutive sampling	Prospetively	South Africa	Southern Africa	Upper-middle-income economies	Unclear/ Not reported	Urban	Hospital-based	One hundred and one southern African blacks with HCC seen prospectively at Baragwanath Hospital just outside Johannesburg were included in the study.	HCC was confirmed histologically in 85 patients: the remainder were diagnosed on a combination of the clinical features, the demonstration of a “mass lesion” on hepatic imaging, and a serum alphafetoprotein concentration above 1000 ng/ml.	Each patient with HCC was matched for ethnic origin, sex and age (? 2 years) with a patient from the same wards of Baragwanath Hospital having a diagnosis other than HCC, excluding rural patients, those considered to have diseases caused by alcohol abuse (avitaminoses, recurrent or chronic pancreatitis, gastritis, alcoholic hepatitis or cirrhosis, peripheral neuropathy, myopathy, cerebellar ataxia, congestive cardiomyopathy), and patients unable to answer questions.	VHB	Chemiluminescent enzyme immunoassay	HBV: HBsAg (+)
Montaser, 2007	Case control	Non probabilistic	Consecutive sampling	Prospetively	Egypt	Northern Africa	Lower-middle-income economies	Unclear/ Not reported	Urban	Hospital-based	Forty-seven patients with HCC and/or CLD were included in the study.	The HCC diagnosis was made by abdominal ultrasonography, and Doppler study.	All participants were divided into 4 groups: Group II were non-tumor 15 patients with CLD.	VHC	Chemiluminescent enzyme immunoassay	HCV: Anti-VHC
Montaser, 2007	Case control	Non probabilistic	Consecutive sampling	Prospetively	Egypt	Northern Africa	Lower-middle-income economies	Unclear/ Not reported	Urban	Hospital-based	Forty-seven patients with HCC and/or CLD were included in the study.	The HCC diagnosis was made by abdominal ultrasonography, and Doppler study.	All participants were divided into 4 groups: Group II were non-tumor 15 patients with CLD.	VHB	Chemiluminescent enzyme immunoassay	HBV: HBsAg (+)
Montaser, 2007	Case control	Non probabilistic	Consecutive sampling	Prospetively	Egypt	Northern Africa	Lower-middle-income economies	Unclear/ Not reported	Urban	Hospital-based	Forty-seven patients with HCC and/or CLD were included in the study.	The HCC diagnosis was made by abdominal ultrasonography, and Doppler study.	In addition, 10 apparently healthy individuals of comparable age and sex were included as a control group.	VHC	Immune adherence haemagglutination	HCV: Anti-VHC
Montaser, 2007	Case control	Non probabilistic	Consecutive sampling	Prospetively	Egypt	Northern Africa	Lower-middle-income economies	Unclear/ Not reported	Urban	Hospital-based	Forty-seven patients with HCC and/or CLD were included in the study.	The HCC diagnosis was made by abdominal ultrasonography, and Doppler study.	In addition, 10 apparently healthy individuals of comparable age and sex were included as a control group.	VHB	Immune adherence haemagglutination	HBV: HBsAg (+)
Nishioka, 1975	Case control	Non probabilistic	Consecutive sampling	Prospetively	Kenya	Eastern Africa	Lower-middle-income economies	Unclear/ Not reported	Unclear/ Not reported	Unclear/ Not reported	Hepatocellular carcinoma	Unclear/ Not reported	Liver cirrhosis	VHB	Enzyme immunoassay	HBV: HBsAg (+)
Nishioka, 1975	Case control	Non probabilistic	Consecutive sampling	Prospetively	Kenya	Eastern Africa	Lower-middle-income economies	Unclear/ Not reported	Unclear/ Not reported	Unclear/ Not reported	Hepatocellular carcinoma	Unclear/ Not reported	Healthy control	VHB	Direct ELISA	HBV: HBsAg (+)
Chin'ombe, 2009	Case control	Non probabilistic	Consecutive sampling	Prospetively	Zimbabwe	Eastern Africa	Lower-middle-income economies	Oct/1999-Aug/2000	Unclear/ Not reported	Hospital-based	The sixty patients were presented to Parirenyatwa Hospital between October 1999 and August 2000 and were diagnosed as having PHC.	The medical records of the patients were also examined for diagnostic tests and evaluations suggesting or confirming primary hepatocellular carcinoma. The tests included ultrasound scanning, X-ray photography, needle aspirate cytological proof and liver biopsy histology. Of the 60 patients recruited in this study, only 6 had liver biopsy evaluation of PHC by histological examination. The rest of the patients' diagnosis depended on ultrasound scanning, cytology (fine needle aspirate), clinical features and biochemical evaluations of liver functions and AFP levels.	Controls (health blood donors) (n = 30) were included in the study.	VHC	Direct ELISA	HCV: Anti-VHC
Chin'ombe, 2009	Case control	Non probabilistic	Consecutive sampling	Prospetively	Zimbabwe	Eastern Africa	Lower-middle-income economies	Oct/1999-Aug/2000	Unclear/ Not reported	Hospital-based	The sixty patients were presented to Parirenyatwa Hospital between October 1999 and August 2000 and were diagnosed as having PHC.	The medical records of the patients were also examined for diagnostic tests and evaluations suggesting or confirming primary hepatocellular carcinoma. The tests included ultrasound scanning, X-ray photography, needle aspirate cytological proof and liver biopsy histology. Of the 60 patients recruited in this study, only 6 had liver biopsy evaluation of PHC by histological examination. The rest of the patients' diagnosis depended on ultrasound scanning, cytology (fine needle aspirate), clinical features and biochemical evaluations of liver functions and AFP levels.	Controls (health blood donors) (n = 30) were included in the study.	VHB	Indirect ELISA	HBV: HBsAg (+)
Ola, 2012	Case control	Non probabilistic	Consecutive sampling	Prospetively	Nigeria	West Africa	Lower-middle-income economies	Unclear/ Not reported	Unclear/ Not reported	Unclear/ Not reported	This prospective case controlled study involved forty one adult Nigerian patients with PHCC and 45 controls (apparently healthy adult without liver diseases) who were enrolled after obtaining informed consent from them.	The patients were diagnosed by clinical features suggestive of PHCC, use of ultrasonography of their liver, elevated serum alphafeotoprotein and confirmed histologically.	This prospective case controlled study involved forty one adult Nigerian patients with PHCC and 45 controls (apparently healthy adult without liver diseases) who were enrolled after obtaining informed consent from them. The controls and the patients were sex and age matched.	VHD	Direct ELISA	HDV: Ag Delta
Ola, 2012	Case control	Non probabilistic	Consecutive sampling	Prospetively	Nigeria	West Africa	Lower-middle-income economies	Unclear/ Not reported	Unclear/ Not reported	Unclear/ Not reported	This prospective case controlled study involved forty one adult Nigerian patients with PHCC and 45 controls (apparently healthy adult without liver diseases) who were enrolled after obtaining informed consent from them.	The patients were diagnosed by clinical features suggestive of PHCC, use of ultrasonography of their liver, elevated serum alphafeotoprotein and confirmed histologically.	This prospective case controlled study involved forty one adult Nigerian patients with PHCC and 45 controls (apparently healthy adult without liver diseases) who were enrolled after obtaining informed consent from them. The controls and the patients were sex and age matched.	VHC	Direct ELISA	HCV: Anti-VHC
Ola, 2012	Case control	Non probabilistic	Consecutive sampling	Prospetively	Nigeria	West Africa	Lower-middle-income economies	Unclear/ Not reported	Unclear/ Not reported	Unclear/ Not reported	This prospective case controlled study involved forty one adult Nigerian patients with PHCC and 45 controls (apparently healthy adult without liver diseases) who were enrolled after obtaining informed consent from them.	The patients were diagnosed by clinical features suggestive of PHCC, use of ultrasonography of their liver, elevated serum alphafeotoprotein and confirmed histologically.	This prospective case controlled study involved forty one adult Nigerian patients with PHCC and 45 controls (apparently healthy adult without liver diseases) who were enrolled after obtaining informed consent from them. The controls and the patients were sex and age matched.	VHB	Enzyme immunoassay	HBV: HBsAg (+)
Ola, 2012	Case control	Non probabilistic	Consecutive sampling	Prospetively	Nigeria	West Africa	Lower-middle-income economies	Unclear/ Not reported	Unclear/ Not reported	Unclear/ Not reported	This prospective case controlled study involved forty one adult Nigerian patients with PHCC and 45 controls (apparently healthy adult without liver diseases) who were enrolled after obtaining informed consent from them.	The patients were diagnosed by clinical features suggestive of PHCC, use of ultrasonography of their liver, elevated serum alphafeotoprotein and confirmed histologically.	This prospective case controlled study involved forty one adult Nigerian patients with PHCC and 45 controls (apparently healthy adult without liver diseases) who were enrolled after obtaining informed consent from them. The controls and the patients were sex and age matched.	VHB	Enzyme immunoassay	HBV: HBeAg (+)
Olubuyide, 1997	Case control	Non probabilistic	Consecutive sampling	Prospetively	Nigeria	West Africa	Lower-middle-income economies	Jan/1995-Sep/1995	Unclear/ Not reported	Hospital-based	During this period, 90 patients with an initial diagnosis of HCC were referred to the unit.	The diagnosis of HCC was based on clinical features, liver tests and echographic suspicion. The HCC cases were then clinicallv examined and subjected to needle liver biopsy; only &e 64 (7 1%) histologically diagnosed as having HCC were used for subsequent analysis.	The 90 control subjects were patients admitted to the hospital during the same period for reasons other than neoplasm or liver disease. They were frequency-matched with HCC cases according to age (?5 years), gender and place of residence, since the prevalence of risk factors probably varies in different areas.	VHC	Indirect ELISA	HCV: Anti-VHC
Olubuyide, 1997	Case control	Non probabilistic	Consecutive sampling	Prospetively	Nigeria	West Africa	Lower-middle-income economies	Jan/1995-Sep/1995	Unclear/ Not reported	Hospital-based	During this period, 90 patients with an initial diagnosis of HCC were referred to the unit.	The diagnosis of HCC was based on clinical features, liver tests and echographic suspicion. The HCC cases were then clinicallv examined and subjected to needle liver biopsy; only &e 64 (7 1%) histologically diagnosed as having HCC were used for subsequent analysis.	The 90 control subjects were patients admitted to the hospital during the same period for reasons other than neoplasm or liver disease. They were frequency-matched with HCC cases according to age (?5 years), gender and place of residence, since the prevalence of risk factors probably varies in different areas.	VHB	Direct ELISA	HBV: HBsAg (+)
Omer, 2001	Case control	Probabilistic	Simple random sampling	Retrospectively	Sudan	Northern Africa	Low-income economies	Sep/1996-Sep/1998	Unclear/ Not reported	Hospital/community based	A total of 150 HCC cases between 2 l-70 years were recruited from the 2 study regions, in a period of 2 successive years; from 115 of these, blood samples were obtained and tested for HBsAg and for HCV antibodies, missing blood samples were attributable to study logistics only. Cases were recruited from 5 out of 6 Khartoum (capital) hospitals (Tropical Medicine Hospital, Oumdurman Hospital, Khartoum Hospital, Soba Hospital and Ibn Sena Hospital).	In these hospitals liver cancers are diagnosed, following referral from the regional hospitals. For all 115 cases, the diagnosis of HCC was verified clinically, by liver function tests and by histopathological examination of a liver biopsy.	As hepatitis infection is one of the present study questions, and since subjects with hepatitis and other diseases may be overrepresented in hospitals, the control group for this study was enrolled from the general population. The controls were community-based subjects, recruited from the same catchment areas as the cases; the number of controls was chosen proportional to the population size of the 2 areas.	VHC	RT-PCR	HCV: Anti-VHC
Omer, 2001	Case control	Probabilistic	Simple random sampling	Retrospectively	Sudan	Northern Africa	Low-income economies	Sep/1996-Sep/1998	Unclear/ Not reported	Hospital/community based	A total of 150 HCC cases between 2 l-70 years were recruited from the 2 study regions, in a period of 2 successive years; from 115 of these, blood samples were obtained and tested for HBsAg and for HCV antibodies, missing blood samples were attributable to study logistics only. Cases were recruited from 5 out of 6 Khartoum (capital) hospitals (Tropical Medicine Hospital, Oumdurman Hospital, Khartoum Hospital, Soba Hospital and Ibn Sena Hospital).	In these hospitals liver cancers are diagnosed, following referral from the regional hospitals. For all 115 cases, the diagnosis of HCC was verified clinically, by liver function tests and by histopathological examination of a liver biopsy.	As hepatitis infection is one of the present study questions, and since subjects with hepatitis and other diseases may be overrepresented in hospitals, the control group for this study was enrolled from the general population. The controls were community-based subjects, recruited from the same catchment areas as the cases; the number of controls was chosen proportional to the population size of the 2 areas.	VHB	PCR	HBV: HBsAg (+)
Schiefelbein, 2012	Case control	Probabilistic	Simple random sampling	Retrospectively	Egypt	Northern Africa	Lower-middle-income economies	Dec/2007-Jan/2009	Unclear/ Not reported	Hospital-based	Cases were all newly-diagnosed liver cancer patients seen at the recruiting hospitals during the study period without age or sex restriction.	About 40% of liver cancer Cases in the recruiting hospitals were diagnosed by histopathological confirmation while the remaining Cases were diagnosed by clinical, biochemical (alpha-fetoprotein), and radiological (ultrasound, triphasicity) confirmation (Ibrahim et al., 2007).	Controls were randomly chosen from non-relative visitors of cancer patients admitted to the study hospitals during the study period.	VHC	Indirect ELISA	HCV: HCV RNA
Schiefelbein, 2012	Case control	Probabilistic	Simple random sampling	Retrospectively	Egypt	Northern Africa	Lower-middle-income economies	Dec/2007-Jan/2009	Unclear/ Not reported	Hospital-based	Cases were all newly-diagnosed liver cancer patients seen at the recruiting hospitals during the study period without age or sex restriction.	About 40% of liver cancer Cases in the recruiting hospitals were diagnosed by histopathological confirmation while the remaining Cases were diagnosed by clinical, biochemical (alpha-fetoprotein), and radiological (ultrasound, triphasicity) confirmation (Ibrahim et al., 2007).	Controls were randomly chosen from non-relative visitors of cancer patients admitted to the study hospitals during the study period.	VHB	Indirect ELISA	HBV: HBV DNA

Schiefelbein, 2012	Case control	Probabilistic	Simple random sampling	Retrospectively	Egypt	Northern Africa	Lower-middle-income economies	Dec/2007-Jan/2009	Unclear/ Not reported	Hospital-based	Cases were all newly-diagnosed liver cancer patients seen at the recruiting hospitals during the study period without age or sex restriction.	About 40% of liver cancer Cases in the recruiting hospitals were diagnosed by histopathological confirmation while the remaining Cases were diagnosed by clinical, biochemical (alpha-fetoprotein), and radiological (ultrasound, triphasicity) confirmation (Ibrahim et al., 2007).	Controls were randomly chosen from non-relative visitors of cancer patients admitted to the study hospitals during the study period.	VHC	Direct ELISA	HCV: Anti-VHC
Skelton, 2000	Case control	Non probabilistic	Consecutive sampling	Retrospectively	South Africa	Southern Africa	Upper-middle-income economies	Unclear/ Not reported	Unclear/ Not reported	Hospital-based	One hundred and forty-eight southern African blacks diagnosed with HCC at four Johannesburg hospitals comprised the case series of the case-control study.	HCC was diagnosed histologically or cytologically by fine needle aspiration, or on a combination of the clinical features, demonstration of one or more mass lesions in the liver on hepatic imaging and a serum a-fetoprotein level of > 500 ng ml)1.	Each patient was matched for race, gender, age (within 3 years), geographical background (rural, urban, or rural-urban) and, when possible, for tribal ethnicity, with a patient from the same hospital and type of ward (medical or surgical), who was diagnosed with a disease other than HCC that was not known to be caused by HBV or HCV and had not been described to be associated with TTV. Only a small minority of the controls suffered from liver disease (amoebic liver abscess, extrahepatic biliary obstruction, penetrating liver injury, Budd-Chiari Syndrome).	VHC	Unclear/Not reported	HCV: Anti-VHC
Skelton, 2000	Case control	Non probabilistic	Consecutive sampling	Retrospectively	South Africa	Southern Africa	Upper-middle-income economies	Unclear/ Not reported	Unclear/ Not reported	Hospital-based	One hundred and forty-eight southern African blacks diagnosed with HCC at four Johannesburg hospitals comprised the case series of the case-control study.	HCC was diagnosed histologically or cytologically by fine needle aspiration, or on a combination of the clinical features, demonstration of one or more mass lesions in the liver on hepatic imaging and a serum a-fetoprotein level of > 500 ng ml)1.	Each patient was matched for race, gender, age (within 3 years), geographical background (rural, urban, or rural-urban) and, when possible, for tribal ethnicity, with a patient from the same hospital and type of ward (medical or surgical), who was diagnosed with a disease other than HCC that was not known to be caused by HBV or HCV and had not been described to be associated with TTV. Only a small minority of the controls suffered from liver disease (amoebic liver abscess, extrahepatic biliary obstruction, penetrating liver injury, Budd-Chiari Syndrome).	VHB	Unclear/Not reported	HBV: HBsAg (+)
Soliman, 2010	Case control	Non probabilistic	Consecutive sampling	Retrospectively	Egypt	Northern Africa	Lower-middle-income economies	Dec/2007-Jan/2009	Unclear/ Not reported	Hospital-based	A total of 150 liver cancer patients representing about 80% of the overall liver cancer patients seen during the period of the study in the Gharbiah population-based cancer registry were recruited in this study.	Cases were diagnosed by radiological and specific tumor markers, and 27.33% of them had histological confirmation.	Controls were recruited from the TCC and GCS from healthy individuals who were visiting or accompanying patients of TCC and GCS during the period of the study. Each control subject was individually matched to cases by sex and age (±5 years).	VHC	Radioimmunoassay	HCV: Anti-VHC
Soliman, 2010	Case control	Non probabilistic	Consecutive sampling	Retrospectively	Egypt	Northern Africa	Lower-middle-income economies	Dec/2007-Jan/2009	Unclear/ Not reported	Hospital-based	A total of 150 liver cancer patients representing about 80% of the overall liver cancer patients seen during the period of the study in the Gharbiah population-based cancer registry were recruited in this study.	Cases were diagnosed by radiological and specific tumor markers, and 27.33% of them had histological confirmation.	Controls were recruited from the TCC and GCS from healthy individuals who were visiting or accompanying patients of TCC and GCS during the period of the study. Each control subject was individually matched to cases by sex and age (±5 years).	VHB	Radioimmunoassay	HBV: HBsAg (+)
Tabor, 1977	Case control	Non probabilistic	Consecutive sampling	Retrospectively	Zambia	Eastern Africa	Lower-middle-income economies	Unclear/ Not reported	Unclear/ Not reported	Hospital/community based	Single serum samples from 47 PHC patients from Uganda, 19 PHC patients from Zambia, and 27 PHC patients from the United States were tested.	Liver biopsies from patients from Uganda, Zambia, and the Southeastern Cancer Study Group were read by one pathologist (Dr. Peter Anthony).	Control sera included 50 hospital in-patients from Uganda with either melanoma or Kaposi's sarcoma and 40 healthy Zambian villagers.	VHB	Indirect ELISA	HBV: HBsAg (+)
Tabor, 1977	Case control	Non probabilistic	Consecutive sampling	Retrospectively	Uganda	Eastern Africa	Low-income economies	Unclear/ Not reported	Unclear/ Not reported	Hospital/community based	Single serum samples from 47 PHC patients from Uganda, 19 PHC patients from Zambia, and 27 PHC patients from the United States were tested.	Liver biopsies from patients from Uganda, Zambia, and the Southeastern Cancer Study Group were read by one pathologist (Dr. Peter Anthony).	Control sera included 50 hospital in-patients from Uganda with either melanoma or Kaposi's sarcoma and 40 healthy Zambian villagers.	VHB	Direct ELISA	HBV: HBsAg (+)
Tswana, 1992	Case control	Probabilistic	Simple random sampling	Retrospectively	Zimbabwe	Eastern Africa	Lower-middle-income economies	Unclear/ Not reported	Unclear/ Not reported	Hospital-based	Two hundred eighty-two patients aged 20-65 years (mean age, 30) were enrolled in the study conducted at Parirenyatwa Teaching Hospital.	Of the 282 subjects, 182 were liver disease patients as defined by raised AFP (>1,000 ng/ml). These patients with raised AFP were classified as HCC patients. The authors appreciate that using an AFP level of >1,000 ng/ml might have most probably excluded some patients with HCC, since no ultrasound or scanner imaging were available. Thus an arbitrary higher level of AFP was used.	The remaining 100 patients, 50 males and 50 females, served as controls, and presented with illnesses other than liver disease. These were randomly selected and had AFP levels of <1,000 ng/ml, and were comparable in age and sex to the HCC patients. All attended the same hospital where the study group was selected.	VHC	Direct ELISA	HCV: Anti-VHC
Tswana, 1992	Case control	Probabilistic	Simple random sampling	Retrospectively	Zimbabwe	Eastern Africa	Lower-middle-income economies	Unclear/ Not reported	Unclear/ Not reported	Hospital-based	Two hundred eighty-two patients aged 20-65 years (mean age, 30) were enrolled in the study conducted at Parirenyatwa Teaching Hospital.	Of the 282 subjects, 182 were liver disease patients as defined by raised AFP (>1,000 ng/ml). These patients with raised AFP were classified as HCC patients. The authors appreciate that using an AFP level of >1,000 ng/ml might have most probably excluded some patients with HCC, since no ultrasound or scanner imaging were available. Thus an arbitrary higher level of AFP was used.	The remaining 100 patients, 50 males and 50 females, served as controls, and presented with illnesses other than liver disease. These were randomly selected and had AFP levels of <1,000 ng/ml, and were comparable in age and sex to the HCC patients. All attended the same hospital where the study group was selected.	VHB	Radioimmunoassay	HBV: HBsAg (+)
Tswana, 1992	Case control	Probabilistic	Simple random sampling	Retrospectively	Zimbabwe	Eastern Africa	Lower-middle-income economies	Unclear/ Not reported	Unclear/ Not reported	Hospital-based	Two hundred eighty-two patients aged 20-65 years (mean age, 30) were enrolled in the study conducted at Parirenyatwa Teaching Hospital.	Of the 282 subjects, 182 were liver disease patients as defined by raised AFP (>1,000 ng/ml). These patients with raised AFP were classified as HCC patients. The authors appreciate that using an AFP level of >1,000 ng/ml might have most probably excluded some patients with HCC, since no ultrasound or scanner imaging were available. Thus an arbitrary higher level of AFP was used.	The remaining 100 patients, 50 males and 50 females, served as controls, and presented with illnesses other than liver disease. These were randomly selected and had AFP levels of <1,000 ng/ml, and were comparable in age and sex to the HCC patients. All attended the same hospital where the study group was selected.	VHB	Radioimmunoassay	HBV: HBeAg (+)
Cenac, 1987	Case control	Non probabilistic	Consecutive sampling	Prospetively	Niger	West Africa	Low-income economies	Oct/1982-Jun/1985	Urban	Community-based	At least one of the following signs was necessary for inclusion in the study: hepatomegaly, jaundice, ascites, oesophageal varices, abdominal venous pattern, or splenomegaly.	HCC was diagnosed on 4 criteria: clinical (abdominal mass), echographical (large tumour with important vascularization), histological and serological (increased serum alpha-foetoprotein).	Unclear/ Not reported	VHD	Radioimmunoassay	HDV: Anti-delta (+)
Cenac, 1987	Case control	Non probabilistic	Consecutive sampling	Prospetively	Niger	West Africa	Low-income economies	Oct/1982-Jun/1985	Urban	Community-based	At least one of the following signs was necessary for inclusion in the study: hepatomegaly, jaundice, ascites, oesophageal varices, abdominal venous pattern, or splenomegaly.	HCC was diagnosed on 4 criteria: clinical (abdominal mass), echographical (large tumour with important vascularization), histological and serological (increased serum alpha-foetoprotein).	Unclear/ Not reported	VHD	Radioimmunoassay	HDV: Anti-delta (+)
Cenac, 1987	Case control	Non probabilistic	Consecutive sampling	Prospetively	Niger	West Africa	Low-income economies	Oct/1982-Jun/1985	Urban	Community-based	At least one of the following signs was necessary for inclusion in the study: hepatomegaly, jaundice, ascites, oesophageal varices, abdominal venous pattern, or splenomegaly.	HCC was diagnosed on 4 criteria: clinical (abdominal mass), echographical (large tumour with important vascularization), histological and serological (increased serum alpha-foetoprotein).	Cirrhosis diagnosis was based on clinical, biological and echographical signs: atrophic liver with increased density, dilated portal vein, small intra-hepatic vessels. When diagnosis was uncertam, confirmation was obtamed by histological findings (needle biopsy).	VHD	Radioimmunoassay	HDV: Anti-delta (+)
Chin'ombe, 2009	Case control	Non probabilistic	Consecutive sampling	Prospetively	Zimbabwe	Eastern Africa	Lower-middle-income economies	Oct/1999-Aug/2000	Unclear/ Not reported	Hospital-based	The sixty patients were presented to Parirenyatwa Hospital between October 1999 and August 2000 and were diagnosed as having PHC.	The medical records of the patients were also examined for diagnostic tests and evaluations suggesting or confirming primary hepatocellular carcinoma. The tests included ultrasound scanning, X-ray photography, needle aspirate cytological proof and liver biopsy histology. Of the 60 patients recruited in this study, only 6 had liver biopsy evaluation of PHC by histological examination. The rest of the patients' diagnosis depended on ultrasound scanning, cytology (fine needle aspirate), clinical features and biochemical evaluations of liver functions and AFP levels.	Controls (health blood donors) (n = 30) were included in the study.	VHB/VHC	Indirect ELISA/Direct ELISA	HBV: HBsAg (+)/HCV: Anti-VHC
Kew, 1990	Case control	Non probabilistic	Consecutive sampling	Retrospectively	South Africa	Southern Africa	Upper-middle-income economies	Unclear/ Not reported	Urban/rural	Unclear/ Not reported	380 unselected southern African blacks (322 male, 58 female) with histologically proven HCC and 152 apparently healthy controls matched for race, sex, age, and rural/urban origin were studied.	380 unselected southern African blacks (322 male, 58 female) with histologically proven HCC and 152 apparently healthy controls matched for race, sex, age, and rural/urban origin were studied.	380 unselected southern African blacks (322 male, 58 female) with histologically proven HCC and 152 apparently healthy controls matched for race, sex, age, and rural/urban origin were studied.	VHB/VHC	Radioimmunoassay/Indirect ELISA	HBV: HBsAg (+)/HCV: Anti-VHC
Olubuyide, 1997	Case control	Non probabilistic	Consecutive sampling	Prospetively	Nigeria	West Africa	Lower-middle-income economies	Jan/1995-Sep/1995	Unclear/ Not reported	Hospital-based	During this period, 90 patients with an initial diagnosis of HCC were referred to the unit.	The diagnosis of HCC was based on clinical features, liver tests and echographic suspicion. The HCC cases were then clinicallv examined and subjected to needle liver biopsy; only &e 64 (7 1%) histologically diagnosed as having HCC were used for subsequent analysis.	The 90 control subjects were patients admitted to the hospital during the same period for reasons other than neoplasm or liver disease. They were frequency-matched with HCC cases according to age (?5 years), gender and place of residence, since the prevalence of risk factors probably varies in different areas.	VHB/VHC	Indirect ELISA/Direct ELISA	HBV: HBsAg (+)/HCV: Anti-VHC
Schiefelbein, 2012	Case control	Probabilistic	Simple random sampling	Retrospectively	Egypt	Northern Africa	Lower-middle-income economies	Dec/2007-Jan/2009	Unclear/ Not reported	Hospital-based	Cases were all newly-diagnosed liver cancer patients seen at the recruiting hospitals during the study period without age or sex restriction.	About 40% of liver cancer Cases in the recruiting hospitals were diagnosed by histopathological confirmation while the remaining Cases were diagnosed by clinical, biochemical (alpha-fetoprotein), and radiological (ultrasound, triphasicity) confirmation (Ibrahim et al., 2007).	Controls were randomly chosen from non-relative visitors of cancer patients admitted to the study hospitals during the study period.	VHB/VHC	PCR/RT-PCR	HBV: HBV DNA/HCV: HCV RNA
Soliman, 2010	Case control	Non probabilistic	Consecutive sampling	Retrospectively	Egypt	Northern Africa	Lower-middle-income economies	Dec/2007-Jan/2009	Unclear/ Not reported	Hospital-based	A total of 150 liver cancer patients representing about 80% of the overall liver cancer patients seen during the period of the study in the Gharbiah population-based cancer registry were recruited in this study.	Cases were diagnosed by radiological and specific tumor markers, and 27.33% of them had histological confirmation.	Controls were recruited from the TCC and GCS from healthy individuals who were visiting or accompanying patients of TCC and GCS during the period of the study. Each control subject was individually matched to cases by sex and age (±5 years).	VHB/VHC	Radioimmunoassay	HBV: HBsAg (+)/HCV: Anti-VHC
