# Supplementary material for: Hepatitis B, C and D virus infections and risk of hepatocellular carcinoma in Africa: A meta-analysis including sensitivity analyses for studies comparable for confounders
Source: PLoS One. 2022 Jan 21;17(1):e0262903. doi: 10.1371/journal.pone.0262903 (PMC8782350; doi:10.1371/journal.pone.0262903)
Supplement: S8 Table — (PDF) [file pone.0262903.s009.pdf]

**S8 Table. P-value of Khi-2 and Fisher exact tests for qualitative confounding factors**

| | | Data extracted from included studies | | | | Results from this study | | |
|---|---|---|---|---|---|---|---|---|
| Author, Year | Qualitative confounding factors | Total number of HCC (+) | Number of HCC (+) with confounding factors | Total number of HCC (-) | Total number of HCC (-) with confounding factors | P-value Khi-2 test | P-value Fisher exact test | Status |
| Amr, 2014 | Born in rural areas | 132 | 109 | 669 | 443 | < 0.05 | < 0.05 | Asymmetric |
| Amr, 2014 | Education (some) | 132 | 40 | 669 | 336 | < 0.05 | < 0.05 | Asymmetric |
| Bahri, 2011 | Diabete mellitus | 139 | 27 | 225 | 7 | < 0.05 | < 0.05 | Asymmetric |
| Bahri, 2011 | Male gender | 164 | 100 | 250 | 152 | 1 | 1 | Symmetric |
| Brown, 1984 | Male gender | 36 | 32 | 70 | 65 | 0.744 | 0.485 | Symmetric |
| Cenac, 1987 | Male gender | 29 | 21 | 55 | 31 | 0.229 | 0.166 | Symmetric |
| Cenac, 1987 | Male gender | 29 | 21 | 46 | 25 | 0.186 | 0.148 | Symmetric |
| Cenac, 1987 | Male gender | 29 | 21 | 28 | 12 | 0.046 | 0.033 | Asymmetric |
| Coursaget�1978 | Male gender | 103 | 90 | 76 | 62 | 0.39 | 0.299 | Symmetric |
| Coursaget�1978 | Male gender | 103 | 90 | 100 | 85 | 0.774 | 0.687 | Symmetric |
| Dhifallah�2020 | Alcohol drinking | 64 | 15 | 61 | 2 | 0.002 | 0.001 | Asymmetric |
| Dhifallah�2020 | Dental care | 48 | 30 | 55 | 9 | < 0.05 | < 0.05 | Asymmetric |
| Dhifallah�2020 | Diabete mellitus | 66 | 21 | 62 | 3 | < 0.05 | < 0.05 | Asymmetric |
| Dhifallah�2020 | Male gender | 73 | 39 | 70 | 29 | 0.205 | 0.181 | Symmetric |
| Dhifallah�2020 | Non-BDC | 73 | 8 | 70 | 59 | < 0.05 | < 0.05 | Asymmetric |
| Dhifallah�2020 | Previous blood transfusion | 59 | 15 | 60 | 6 | 0.049 | 0.032 | Asymmetric |
| Dhifallah�2020 | Smoke exposure | 62 | 23 | 61 | 8 | 0.004 | 0.003 | Asymmetric |
| Dhifallah�2020 | Surgical antecedents | 66 | 31 | 70 | 3 | < 0.05 | < 0.05 | Asymmetric |
| Dhifallah�2020 | Tattoos-scarifications | 58 | 34 | 58 | 34 | 1 | 1 | Symmetric |
| Ezzat�2005 | Agricultural pesticides | 236 | 86 | 236 | 58 | 0.007 | 0.007 | Asymmetric |
| Ezzat�2005 | Alcohol drinking | 236 | 1 | 236 | 9 | 0.025 | 0.02 | Asymmetric |
| Ezzat�2005 | Born in rural areas | 236 | 194 | 236 | 182 | 0.208 | 0.208 | Symmetric |
| Ezzat�2005 | Current shisha smoking | 236 | 12 | 236 | 25 | 0.04 | 0.039 | Asymmetric |
| Ezzat�2005 | Education (some) | 236 | 77 | 236 | 73 | 0.767 | 0.767 | Symmetric |
| Ezzat�2005 | Ever helping in farming | 236 | 45 | 236 | 33 | 0.173 | 0.173 | Symmetric |
| Ezzat�2005 | Ever working in farming | 236 | 87 | 236 | 74 | 0.244 | 0.244 | Symmetric |
| Ezzat�2005 | Male gender | 236 | 176 | 236 | 176 | 1 | 1 | Symmetric |
| Ezzat�2005 | Married | 236 | 219 | 236 | 191 | < 0.05 | < 0.05 | Asymmetric |
| Ezzat�2005 | Pesticides at home | 236 | 146 | 236 | 146 | 1 | 1 | Symmetric |
| Ezzat�2005 | Rodenticides at field | 236 | 59 | 236 | 43 | 0.093 | 0.093 | Symmetric |
| Ezzat�2005 | Rodenticides at home | 236 | 29 | 236 | 23 | 0.462 | 0.463 | Symmetric |
| Ezzat�2005 | Smoke exposure | 236 | 70 | 236 | 90 | 0.065 | 0.065 | Symmetric |
| Gouas, 2012 | Male gender | 198 | 160 | 78 | 50 | 0.006 | 0.005 | Asymmetric |
| Gouas, 2012 | Male gender | 198 | 160 | 325 | 224 | 0.004 | 0.003 | Asymmetric |
| Hassan, 2001 | Alcohol drinking | 33 | 2 | 35 | 6 | 0.298 | 0.26 | Symmetric |
| Hassan, 2001 | Anti-Schistosoma IgG | 33 | 7 | 35 | 5 | 0.667 | 0.534 | Symmetric |
| Hassan, 2001 | Male gender | 33 | 23 | 35 | 14 | 0.027 | 0.017 | Asymmetric |
| Hassan, 2001 | Oral contraceptive user | 33 | 3 | 35 | 6 | 0.534 | 0.478 | Symmetric |
| Hassan, 2001 | Smoke exposure | 33 | 15 | 35 | 16 | 1 | 1 | Symmetric |
| Jaquet, 2018 | Alcohol drinking | 40 | 29 | 80 | 60 | 0.941 | 0.826 | Symmetric |
| Jaquet, 2018 | Alcohol drinking | 44 | 17 | 88 | 48 | 0.124 | 0.099 | Symmetric |
| Jaquet, 2018 | Alcohol drinking | 76 | 2 | 152 | 2 | 0.858 | 0.602 | Symmetric |
| Jaquet, 2018 | Education (some) | 40 | 27 | 80 | 71 | 0.01 | 0.011 | Asymmetric |

| | | | | | | | | |
|---|---|---|---|---|---|---|---|---|
| Jaquet, 2018 | Education (some) | 44 | 35 | 88 | 80 | 0.118 | 0.096 | Symmetric |
| Jaquet, 2018 | Education (some) | 76 | 32 | 152 | 79 | 0.206 | 0.206 | Symmetric |
| Jaquet, 2018 | HIV coinfection | 40 | 1 | 80 | 4 | 0.872 | 0.664 | Symmetric |
| Jaquet, 2018 | HIV coinfection | 44 | 6 | 88 | 3 | 0.067 | 0.059 | Symmetric |
| Jaquet, 2018 | HIV coinfection | 76 | 0 | 152 | 2 | 0.802 | 0.554 | Symmetric |
| Jaquet, 2018 | Smoke exposure | 40 | 2 | 80 | 0 | 0.207 | 0.109 | Symmetric |
| Jaquet, 2018 | Smoke exposure | 44 | 9 | 88 | 38 | 0.017 | 0.012 | Asymmetric |
| Jaquet, 2018 | Smoke exposure | 76 | 33 | 152 | 54 | 0.311 | 0.252 | Symmetric |
| Kew, 1986 | Male gender | 392 | 330 | 392 | 330 | 1 | 1 | Symmetric |
| Kew, 1986 | Residence in rural area | 392 | 207 | 392 | 324 | < 0.05 | < 0.05 | Asymmetric |
| Kirk, 2005 | Male gender | 186 | 151 | 98 | 64 | 0.005 | 0.004 | Asymmetric |
| Kirk, 2005 | Male gender | 186 | 151 | 348 | 243 | 0.006 | 0.005 | Asymmetric |
| Larouzé, 1977 | Male gender | 21 | 19 | 40 | 37 | 1 | 1 | Symmetric |
| Larouzé, 1977 | Male gender | 39 | 29 | 59 | 41 | 0.769 | 0.654 | Symmetric |
| Lightfoot, 1997 | Male gender | 167 | 147 | 167 | 147 | 1 | 1 | Symmetric |
| Lightfoot, 1997 | Presence of HGV | 167 | 19 | 167 | 21 | 0.866 | 0.866 | Symmetric |
| Mahale, 2019 | Alcohol drinking | 300 | 40 | 114 | 10 | 0.27 | 0.239 | Symmetric |
| Mahale, 2019 | Alcohol drinking | 414 | 50 | 457 | 39 | 0.107 | 0.093 | Symmetric |
| Mahale, 2019 | Earth floor house | 303 | 177 | 117 | 74 | 0.427 | 0.377 | Symmetric |
| Mahale, 2019 | Earth floor house | 420 | 251 | 464 | 233 | 0.005 | 0.005 | Asymmetric |
| Mahale, 2019 | Education (some) | 306 | 240 | 117 | 92 | 1 | 1 | Symmetric |
| Mahale, 2019 | Education (some) | 423 | 332 | 464 | 412 | < 0.05 | < 0.05 | Asymmetric |
| Mahale, 2019 | Family history of cancer | 306 | 30 | 117 | 8 | 0.445 | 0.447 | Symmetric |
| Mahale, 2019 | Family history of cancer | 423 | 38 | 461 | 17 | 0.002 | 0.001 | Asymmetric |
| Mahale, 2019 | Male gender | 312 | 251 | 119 | 78 | 0.002 | 0.002 | Asymmetric |
| Mahale, 2019 | Male gender | 431 | 329 | 470 | 329 | 0.039 | 0.035 | Asymmetric |
| Mahale, 2019 | Married | 306 | 272 | 117 | 99 | 0.302 | 0.248 | Symmetric |
| Mahale, 2019 | Married | 423 | 371 | 463 | 401 | 0.699 | 0.688 | Symmetric |
| Mahale, 2019 | Ser-249 TP53 mutation | 164 | 66 | 99 | 15 | < 0.05 | < 0.05 | Asymmetric |
| Mahale, 2019 | Ser-249 TP53 mutation | 263 | 81 | 349 | 17 | < 0.05 | < 0.05 | Asymmetric |
| Mahale, 2019 | Smoke exposure | 300 | 164 | 117 | 48 | 0.017 | 0.016 | Asymmetric |
| Mahale, 2019 | Smoke exposure | 417 | 212 | 461 | 184 | 0.001 | 0.001 | Asymmetric |
| Mak, 2018 | Alcohol drinking | 150 | 73 | 438 | 213 | 1 | 1 | Symmetric |
| Mak, 2018 | Born in rural areas | 150 | 98 | 438 | 241 | 0.035 | 0.028 | Asymmetric |
| Mak, 2018 | Diabete mellitus | 150 | 5 | 438 | 23 | 0.466 | 0.505 | Symmetric |
| Mak, 2018 | HIV coinfection | 150 | 28 | 438 | 113 | 0.098 | 0.096 | Symmetric |
| Mak, 2018 | Male gender | 150 | 111 | 438 | 324 | 1 | 1 | Symmetric |
| Mak, 2018 | Married | 150 | 122 | 438 | 353 | 0.938 | 0.905 | Symmetric |
| Mak, 2018 | Residence in rural area | 149 | 11 | 437 | 44 | 0.419 | 0.416 | Symmetric |
| Mak, 2018 | Smoke exposure | 150 | 79 | 438 | 223 | 0.782 | 0.777 | Symmetric |
| Mandishona, 1998 | Alcohol drinking | 24 | 13 | 48 | 18 | 0.274 | 0.212 | Symmetric |
| Mandishona, 1998 | Iron overload | 24 | 5 | 48 | 3 | 0.145 | 0.107 | Symmetric |
| Mandishona, 1998 | Male gender | 24 | 20 | 48 | 40 | 1 | 1 | Symmetric |
| Mandishona, 1998 | Other alcoholic beverages | 24 | 8 | 48 | 17 | 1 | 1 | Symmetric |
| Mandishona, 1998 | Traditional beer | 24 | 12 | 48 | 7 | 0.003 | 0.004 | Asymmetric |
| Marchio, 2018 | Alcohol drinking | 195 | 99 | 49 | 5 | < 0.05 | < 0.05 | Asymmetric |
| Marchio, 2018 | Alcohol drinking | 195 | 99 | 263 | 64 | < 0.05 | < 0.05 | Asymmetric |
| Marchio, 2018 | Anti-HEV (IgG and/or IgM) | 195 | 76 | 49 | 0 | < 0.05 | < 0.05 | Asymmetric |
| Marchio, 2018 | Anti-HEV (IgG and/or IgM) | 195 | 76 | 263 | 62 | 0.001 | < 0.05 | Asymmetric |

| | | | | | | | | |
|---|---|---|---|---|---|---|---|---|
| Marchio, 2018 | Diabete mellitus | 195 | 19 | 49 | 2 | 0.328 | 0.265 | Symmetric |
| Marchio, 2018 | Diabete mellitus | 195 | 19 | 263 | 16 | 0.201 | 0.158 | Symmetric |
| Marchio, 2018 | Diet cassava consumption | 195 | 108 | 49 | 21 | 0.158 | 0.149 | Symmetric |
| Marchio, 2018 | Diet cassava consumption | 195 | 108 | 263 | 154 | 0.56 | 0.505 | Symmetric |
| Marchio, 2018 | Diet groundnut consumption | 195 | 1 | 49 | 0 | 1 | 1 | Symmetric |
| Marchio, 2018 | Diet groundnut consumption | 195 | 1 | 263 | 53 | < 0.05 | < 0.05 | Asymmetric |
| Marchio, 2018 | Diet maize consumption | 195 | 57 | 49 | 9 | 0.177 | 0.151 | Symmetric |
| Marchio, 2018 | Diet maize consumption | 195 | 57 | 263 | 73 | 0.809 | 0.754 | Symmetric |
| Marchio, 2018 | Diet millet consumption | 195 | 102 | 49 | 10 | < 0.05 | < 0.05 | Asymmetric |
| Marchio, 2018 | Diet millet consumption | 195 | 102 | 263 | 69 | < 0.05 | < 0.05 | Asymmetric |
| Marchio, 2018 | Diet plantain consumption | 195 | 111 | 49 | 14 | 0.001 | < 0.05 | Asymmetric |
| Marchio, 2018 | Diet plantain consumption | 195 | 111 | 263 | 121 | 0.027 | 0.023 | Asymmetric |
| Marchio, 2018 | Diet rice cunsumption | 195 | 11 | 49 | 3 | 1 | 1 | Symmetric |
| Marchio, 2018 | Diet rice cunsumption | 195 | 11 | 263 | 24 | 0.226 | 0.213 | Symmetric |
| Marchio, 2018 | Diet sorghum consumption | 195 | 39 | 49 | 8 | 0.704 | 0.687 | Symmetric |
| Marchio, 2018 | Diet sorghum consumption | 195 | 39 | 263 | 50 | 0.885 | 0.812 | Symmetric |
| Marchio, 2018 | HIV coinfection | 195 | 47 | 49 | 1 | 0.001 | < 0.05 | Asymmetric |
| Marchio, 2018 | HIV coinfection | 195 | 47 | 263 | 40 | 0.023 | 0.022 | Asymmetric |
| Marchio, 2018 | Male gender | 195 | 144 | 49 | 41 | 0.211 | 0.192 | Symmetric |
| Marchio, 2018 | Male gender | 195 | 144 | 263 | 168 | 0.031 | 0.026 | Asymmetric |
| Marchio, 2018 | Non-BDC | 195 | 47 | 49 | 43 | < 0.05 | < 0.05 | Asymmetric |
| Marchio, 2018 | Non-BDC | 195 | 47 | 263 | 47 | 0.13 | 0.128 | Symmetric |
| Marchio, 2018 | Obesity | 195 | 19 | 49 | 0 | 0.048 | 0.017 | Asymmetric |
| Marchio, 2018 | Obesity | 195 | 19 | 263 | 7 | 0.002 | 0.002 | Asymmetric |
| Marchio, 2018 | Smoke exposure | 195 | 18 | 49 | 3 | 0.683 | 0.775 | Symmetric |
| Marchio, 2018 | Smoke exposure | 195 | 18 | 263 | 18 | 0.446 | 0.382 | Symmetric |
| Mboto, 2005 | Male gender | 13 | 11 | 39 | 33 | 1 | 1 | Symmetric |
| Mets, 1993 | Male gender | 26 | 19 | 54 | 49 | 0.082 | 0.05 | Asymmetric |
| Mets, 1993 | Male gender | 26 | 19 | 79 | 44 | 0.181 | 0.166 | Symmetric |
| Mohamed, 1992 | Alcohol drinking | 101 | 65 | 101 | 52 | 0.087 | 0.087 | Symmetric |
| Mohamed, 1992 | Male gender | 101 | 77 | 101 | 77 | 1 | 1 | Symmetric |
| Mohamed, 1992 | Smoke exposure | 101 | 53 | 101 | 41 | 0.121 | 0.121 | Symmetric |
| Montaser, 2007 | Liver cirrhosis | 32 | 32 | 15 | 15 | NA | 1 | Symmetric |
| Montaser, 2007 | Liver cirrhosis | 32 | 32 | 10 | 0 | < 0.05 | < 0.05 | Asymmetric |
| Montaser, 2007 | Male gender | 32 | 20 | 15 | 11 | 0.689 | 0.527 | Symmetric |
| Montaser, 2007 | Male gender | 32 | 20 | 10 | 4 | 0.374 | 0.281 | Symmetric |
| Ola, 2012 | Male gender | 41 | 31 | 45 | 33 | 1 | 1 | Symmetric |
| Olubuyide, 1997 | Male gender | 64 | 42 | 64 | 42 | 1 | 1 | Symmetric |
| Olubuyide, 1997 | Occupation, None | 64 | 5 | 64 | 3 | 0.715 | 0.718 | Symmetric |
| Olubuyide, 1997 | Previous blood transfusion | 64 | 11 | 64 | 9 | 0.808 | 0.808 | Symmetric |
| Olubuyide, 1997 | Surgical antecedents | 75 | 64 | 79 | 64 | 0.617 | 0.524 | Symmetric |
| Olubuyide, 1997 | Tattoos-scarifications | 64 | 48 | 64 | 46 | 0.841 | 0.842 | Symmetric |
| Olubuyide, 1997 | Use of intravenous drug | 64 | 0 | 64 | 0 | NA | 1 | Symmetric |
| Omer, 2001 | Alcohol drinking | 115 | 45 | 199 | 61 | 0.16 | 0.138 | Symmetric |
| Omer, 2001 | Education (some) | 115 | 35 | 199 | 131 | < 0.05 | < 0.05 | Asymmetric |
| Omer, 2001 | Male gender | 115 | 88 | 199 | 150 | 0.927 | 0.892 | Symmetric |
| Schiefelbein, 2012 | Male gender | 148 | 123 | 148 | 123 | 1 | 1 | Symmetric |
| Schiefelbein, 2012 | Residence in rural area | 148 | 134 | 148 | 131 | 0.704 | 0.705 | Symmetric |
| Soliman, 2010 | Cancer family history | 149 | 12 | 150 | 15 | 0.7 | 0.687 | Symmetric |

| | | | | | | | | |
|---|---|---|---|---|---|---|---|---|
| Soliman, 2010 | Education (some) | 150 | 113 | 150 | 74 | < 0.05 | < 0.05 | Asymmetric |
| Soliman, 2010 | Liver cirrhosis | 142 | 63 | 141 | 17 | < 0.05 | < 0.05 | Asymmetric |
| Soliman, 2010 | Male gender | 150 | 126 | 150 | 126 | 1 | 1 | Symmetric |
| Soliman, 2010 | Married | 150 | 127 | 150 | 130 | 0.742 | 0.742 | Symmetric |
| Soliman, 2010 | Previous blood transfusion | 149 | 29 | 150 | 6 | < 0.05 | < 0.05 | Asymmetric |
| Soliman, 2010 | Smoke exposure | 150 | 83 | 150 | 79 | 0.728 | 0.728 | Symmetric |
| Tabor, 1977 | Male gender | 19 | 17 | 40 | 20 | 0.008 | 0.004 | Asymmetric |
| Tabor, 1977 | Male gender | 47 | 32 | 50 | 43 | 0.062 | 0.052 | Symmetric |
| Tswana, 1992 | Previous blood transfusion | 182 | 6 | 100 | 2 | 0.801 | 0.716 | Symmetric |
| Tswana, 1992 | Tattoos-scarifications | 182 | 24 | 100 | 6 | 0.095 | 0.07 | Symmetric |
