# Supplementary material for: Hepatitis B, C and D virus infections and risk of hepatocellular carcinoma in Africa: A meta-analysis including sensitivity analyses for studies comparable for confounders
Source: PLoS One. 2022 Jan 21;17(1):e0262903. doi: 10.1371/journal.pone.0262903 (PMC8782350; doi:10.1371/journal.pone.0262903)
Supplement: S9 Table — (PDF) [file pone.0262903.s010.pdf]

**S9 Table. P-value of Student test for quantitative confounding factors**

| Author, Year | Quantitative confounding factors | Total number of HCC (+) | Mean for HCC (+) | SD for HCC (+) | Total number of HCC (-) | Mean for HCC (-) | SD for HCC (-) | P-Value for Student Test with unequal variance | Status |
|---|---|---|---|---|---|---|---|---|---|
| Amr, 2014 | Age | 132 | 52.2 | 10.5 | 669 | 45.4 | 14.9 | 0.000 | Asymmetric |
| Amr, 2014 | Number of children | 132 | 6.3 | 2.7 | 669 | 4.7 | 2.6 | 0.000 | Asymmetric |
| Amr, 2014 | Number of pregnancies | 132 | 7.3 | 3.4 | 669 | 5.6 | 3.3 | 0.000 | Asymmetric |
| Bahri, 2011 | Age | 164 | 62.0 | 10.4 | 250 | 58.0 | 10.6 | 0.000 | Asymmetric |
| Dhifallah�2020 | Alanine Aminotransferase (ALT) (IU/mL) | 73 | 82.0 | 55.0 | 70 | 47.0 | 47.0 | 0.000 | Asymmetric |
| Dhifallah�2020 | Alkaline phosphate (UI/mL) | 73 | 368.0 | 355.0 | 70 | 185.0 | 102.0 | 0.000 | Asymmetric |
| Dhifallah�2020 | De Ritis (ASAT/ALAT) ratio | 73 | 1.3 | 0.5 | 70 | 1.0 | 0.3 | 0.000 | Asymmetric |
| Dhifallah�2020 | GammaGT (UI/mL) | 73 | 116.0 | 118.0 | 70 | 45.0 | 77.0 | 0.000 | Asymmetric |
| Dhifallah�2020 | Aspartate aminotransferase (AST) (IU/mL) | 73 | 67.0 | 52.0 | 70 | 48.0 | 41.0 | 0.008 | Asymmetric |
| Dhifallah�2020 | Age | 73 | 63.2 | 9.5 | 70 | 63.7 | 10.9 | 0.385 | Symmetric |
| Larouzé, 1977 | Age | 39 | 41.5 | 11.7 | 59 | 38.8 | 12.8 | 0.143 | Symmetric |
| Larouzé, 1977 | Age | 21 | 49.8 | 13.2 | 40 | 47.7 | 12.6 | 0.277 | Symmetric |
| Mak, 2018 | Age | 150 | 46.1 | 13.3 | 438 | 45.7 | 13.3 | 0.375 | Symmetric |
| Mandishona, 1998 | Age | 24 | 24.8 | 17.7 | 48 | 41.3 | 15.0 | 0.000 | Asymmetric |
| Mandishona, 1998 | Serum ferritin (ng/mL) | 24 | 4725.3 | 4616.8 | 48 | 1529.5 | 1293.8 | 0.001 | Asymmetric |
| Mandishona, 1998 | White blood cells (31023/µL) | 24 | 11.8 | 5.2 | 48 | 8.1 | 3.9 | 0.002 | Asymmetric |
| Mandishona, 1998 | Erythrocyte sedementation rate (mm/hr) | 24 | 65.5 | 29.3 | 48 | 54.8 | 26.7 | 0.069 | Symmetric |
| Mandishona, 1998 | Aflatoxin B1-albumin adducts (pg/mg) | 24 | 27.1 | 22.8 | 48 | 22.3 | 10.2 | 0.167 | Symmetric |
| Mandishona, 1998 | Transferrin saturation (%) | 24 | 38.3 | 27.0 | 48 | 37.5 | 18.9 | 0.452 | Symmetric |
| Mandishona, 1998 | Hemoglobin (g/dL) | 24 | 12.7 | 3.5 | 48 | 12.7 | 1.4 | 0.474 | Symmetric |
| Marchio, 2018 | Age | 195 | 47.1 | 16.6 | 263 | 41.2 | 15.5 | 0.000 | Asymmetric |
| Marchio, 2018 | Alanine Aminotransferase (ALT) (IU/mL) | 195 | 70.0 | 4.0 | 263 | 122.0 | 36.0 | 0.000 | Asymmetric |
| Marchio, 2018 | Aspartate aminotransferase (AST) (IU/mL) | 195 | 164.0 | 15.0 | 263 | 186.0 | 57.0 | 0.000 | Asymmetric |
| Marchio, 2018 | De Ritis (ASAT/ALAT) ratio | 195 | 2.7 | 0.2 | 263 | 1.9 | 0.1 | 0.000 | Asymmetric |
| Marchio, 2018 | Age | 195 | 47.1 | 16.6 | 49 | 42.7 | 16.3 | 0.048 | Asymmetric |
| Mets, 1993 | Age | 26 | 52.0 | 13.0 | 54 | 30.0 | 9.0 | 0.000 | Asymmetric |
| Mets, 1993 | Age | 26 | 52.0 | 13.0 | 79 | 51.0 | 13.0 | 0.368 | Symmetric |
| Mohamed, 1992 | Age | 101 | 53.7 | 1.9 | 101 | 53.8 | 1.8 | 0.349 | Symmetric |
| Olubuyide, 1997 | Age | 64 | 52.4 | 15.8 | 64 | 52.3 | 15.7 | 0.486 | Symmetric |
| Skelton, 2000 | Age | 148 | 45.4 | 15.3 | 148 | 45.3 | 15.2 | 0.478 | Symmetric |
| Soliman, 2010 | Age | 150 | 56.0 | 10.6 | 150 | 55.4 | 10.3 | 0.312 | Symmetric |
