# Supplementary material for: Hepatitis B, C and D virus infections and risk of hepatocellular carcinoma in Africa: A meta-analysis including sensitivity analyses for studies comparable for confounders
Source: PLoS One. 2022 Jan 21;17(1):e0262903. doi: 10.1371/journal.pone.0262903 (PMC8782350; doi:10.1371/journal.pone.0262903)
Supplement: S10 Table — (PDF) [file pone.0262903.s011.pdf]

S10 Table. Subgroup analyses of hepatocellular carcinoma development in people with and without viral hepatitis infections in Africa.

	OR (95%CI)	95% Prediction interval	N Studies	N HCC cases	N controls	H (95%CI)	I² (95%CI)	P-value heterogeneity	P-value Egger test	P-value subgroup difference
**HBV**										
**HBeAg**										
**Healthy controls**										
**Timing of exposure collection**										0.001
Prospetively	1 [0.3-3.3]	[0-1911.9]	3	319	195	1.4 [1-2.5]	46.3 [0-84.2]	0.155	0.968	
Retrospectively	22 [5.1-95]	NA	1	186	348	NA	NA	1	NA	
**Country**										0.001
Gambia	22 [5.1-95]	NA	1	186	348	NA	NA	1	NA	
Nigeria	13.7 [0.7-256.2]	NA	1	41	45	NA	NA	1	NA	
Senegal	0.6 [0.2-2.2]	NA	2	278	150	1	0	0.727	NA	
**Country income level**										0.001
Low-income economies	22 [5.1-95]	NA	1	186	348	NA	NA	1	NA	
Lower-middle-income economies	1 [0.3-3.3]	[0-1911.9]	3	319	195	1.4 [1-2.5]	46.3 [0-84.2]	0.155	0.968	
**Year of publication**										< 0.001
1975-2000	0.6 [0.2-2.2]	NA	2	278	150	1	0	0.727	NA	
2000-2020	20 [5.4-74.1]	NA	2	227	393	1	0	0.777	NA	
**Detection assay**										0.001
Agar gel diffusion	0.6 [0.2-2.2]	NA	2	278	150	1	0	0.727	NA	
Direct ELISA	13.7 [0.7-256.2]	NA	1	41	45	NA	NA	1	NA	
Enzyme immunoassay	22 [5.1-95]	NA	1	186	348	NA	NA	1	NA	
**HBsAg**										
**Healthy controls**										
**Sampling**										0.806

	OR (95%CI)	95% Prediction interval	N Studies	N HCC cases	N controls	H (95%CI)	I² (95%CI)	P-value heterogeneity	P-value Egger test	P-value subgroup difference
Non probabilistic	8.9 [5.9-13.5]	[2-39.9]	17	2065	2388	2 [1.6-2.5]	74.7 [59.3-84.2]	0	0.235	
Probabilistic	9.8 [5.1-18.9]	NA	1	115	199	NA	NA	1	NA	
**Timing of exposure collection**										0.875
Prospetively	8.4 [4.4-15.9]	[1.1-64.8]	11	595	985	2.2 [1.6-2.9]	79.1 [63.1-88.1]	0	0.433	
Retrospectively	8.8 [6.7-11.7]	[4.4-18]	7	1585	1602	1.6 [1-2.4]	60.4 [9.2-82.7]	0.019	0.022	
**Country**										0.005
Egypt	8.5 [4.8-15.2]	[0.2-357.5]	3	213	195	1 [1-1.3]	0 [0-43.2]	0.833	0.746	
Gambia	6.5 [5.1-8.2]	[3.8-11]	4	666	927	1.4 [1-2.5]	51.2 [0-83.9]	0.104	0.424	
Kenya	24.7 [11.7-52.3]	NA	1	42	450	NA	NA	1	NA	
Mali	16.4 [3.1-86.2]	NA	1	21	38	NA	NA	1	NA	
Nigeria	155.6 [8.9-2707.4]	NA	1	41	45	NA	NA	1	NA	
Rwanda	8.4 [2.5-27.9]	NA	1	26	54	NA	NA	1	NA	
Senegal	4.7 [1.5-15.1]	[0-7843490]	3	327	284	3.5 [2.2-5.6]	92 [79.9-96.8]	0	0.293	
South Africa	13.3 [8.9-19.9]	NA	2	669	365	1	0	0.703	NA	
Sudan	9.8 [5.1-18.9]	NA	1	115	199	NA	NA	1	NA	
Zimbabwe	6.1 [1.9-19.6]	NA	1	60	30	NA	NA	1	NA	
**UNSD Region**										0.427
Eastern Africa	12.6 [5.9-26.7]	[0-17416.9]	3	128	534	1.6 [1-2.9]	58.9 [0-88.3]	0.088	0.597	
Northern Africa	9.1 [5.9-14]	[3.5-23.5]	4	328	394	1 [1-1]	0 [0-0.7]	0.927	0.707	
Southern Africa	13.3 [8.9-19.9]	NA	2	669	365	1	0	0.703	NA	
West Africa	7.7 [3.9-15.3]	[0.9-69.4]	9	1055	1294	2.2 [1.6-3.1]	80.2 [63.1-89.3]	0	0.47	
**Country income level**										0.021
Low-income economies	7 [5.6-8.7]	[5.2-9.3]	7	828	1218	1.2 [1-1.8]	30.8 [0-70.5]	0.193	0.136	
Lower-middle-income economies	8.5 [4.2-17.4]	[0.9-82.6]	9	683	1004	2.4 [1.8-3.3]	82.8 [68.8-90.6]	0	0.44	
Upper-middle-income economies	13.3 [8.9-19.9]	NA	2	669	365	1	0	0.703	NA	
**Year of publication**										0.251

	OR (95%CI)	95% Prediction interval	N Studies	N HCC cases	N controls	H (95%CI)	I² (95%CI)	P-value heterogeneity	P-value Egger test	P-value subgroup difference
1975-2000	10.5 [5.4-20.4]	[1.1-96.1]	9	1121	1261	2.6 [1.9-3.4]	85 [73.2-91.5]	0	0.283	
2000-2020	7 [5.7-8.6]	[5.4-9]	9	1059	1326	1 [1-1.7]	0 [0-63.7]	0.457	0.519	
**Detection assay**										0.030
Chemiluminescent enzyme immunoassay	18.6 [1-344.3]	NA	1	32	10	NA	NA	1	NA	
Direct ELISA	8.6 [5.2-14.3]	[3.8-19.7]	5	262	348	1.2 [1-1.8]	26.3 [0-70.7]	0.246	0.555	
Enzyme immunoassay	6 [4-8.9]	NA	2	212	402	1	0	0.559	NA	
Immune adherence haemagglutination	24.7 [11.7-52.3]	NA	1	42	450	NA	NA	1	NA	
Radioimmunoassay	9.5 [4.3-21.3]	[0.7-133]	7	1053	757	2.8 [2-3.8]	86.8 [75.1-93]	0	0.786	
Reverse passive hemagglutination assay	6.7 [5-9.2]	NA	1	431	470	NA	NA	1	NA	
**Liver cirrhosis**										
**Timing of exposure collection**										0.943
Prospectively	1.2 [0.6-2.5]	[0.1-15]	5	249	276	2 [1.3-3.2]	76.1 [41.7-90.2]	0.002	0.47	
Retrospectively	1.1 [0.8-1.6]	NA	2	498	213	1	0	0.774	NA	
**Country**										0.051
Gambia	1.1 [0.8-1.6]	NA	2	498	213	1	0	0.774	NA	
Kenya	0.5 [0.1-1.5]	NA	1	42	18	NA	NA	1	NA	
Niger	1.2 [0.4-3.2]	NA	1	29	55	NA	NA	1	NA	
Rwanda	4.4 [1.6-11.5]	NA	1	26	79	NA	NA	1	NA	
Senegal	0.9 [0.4-2.5]	NA	2	152	124	2.5 [1.2-5.1]	84.3 [35.7-96.2]	0.012	NA	
**UNSD Region**										0.672
Eastern Africa	1.5 [0.3-7.1]	NA	2	68	97	2.8 [1.4-5.6]	87.6 [51.8-96.8]	0.005	NA	
West Africa	1.1 [0.8-1.4]	[0.7-1.7]	5	679	392	1.3 [1-2.2]	44.8 [0-79.7]	0.124	0.959	
**Country income level**										0.242
Low-income economies	1.3 [1-1.7]	[0.7-2.5]	4	553	347	1.5 [1-2.6]	54.6 [0-85]	0.085	0.291	

	OR (95%CI)	95% Prediction interval	N Studies	N HCC cases	N controls	H (95%CI)	I² (95%CI)	P-value heterogeneity	P-value Egger test	P-value subgroup difference
Lower-middle-income economies	0.8 [0.4-1.7]	[0-3515.4]	3	194	142	1.9 [1-3.5]	72.4 [6.8-91.8]	0.027	0.951	
**Setting**										0.966
Community-based	1.2 [0.4-3.2]	NA	1	29	55	NA	NA	1	NA	
Hospital-based	1.2 [0.6-2.3]	[0.1-20.4]	4	627	368	2.1 [1.3-3.4]	77 [37.2-91.6]	0.005	0.591	
**Year of publication**										0.943
1975-2000	1.2 [0.6-2.5]	[0.1-15]	5	249	276	2 [1.3-3.2]	76.1 [41.7-90.2]	0.002	0.47	
2000-2020	1.1 [0.8-1.6]	NA	2	498	213	1	0	0.774	NA	
**Detection assay**										0.267
Enzyme immunoassay	2 [0.9-4.6]	NA	2	212	173	2.3 [1.1-4.7]	80.9 [18.5-95.5]	0.022	NA	
Immune adherence haemagglutination	0.5 [0.1-1.5]	NA	1	42	18	NA	NA	1	NA	
Radioimmunoassay	1 [0.5-2]	[0-1816.5]	3	181	179	1.8 [1-3.4]	69.9 [0-91.2]	0.036	0.509	
Reverse passive hemagglutination assay	1.1 [0.7-1.7]	NA	1	312	119	NA	NA	1	NA	
**Non-hepatic diseases**										
**Sampling**										0.933
Non probabilistic	9.9 [6.1-16.2]	[1.4-73.2]	18	1722	2373	2.2 [1.8-2.8]	79.7 [68.7-86.9]	0	0.242	
Probabilistic	10.3 [5.2-20.6]	NA	1	182	100	NA	NA	1	NA	
**Timing of exposure collection**										0.347
Prospetively	7.5 [3.2-17.6]	[0.4-131.8]	8	830	882	2.4 [1.7-3.3]	82.2 [66.1-90.6]	0	0.29	
Retrospectively	11.9 [7.6-18.9]	[2.7-52.1]	11	1074	1591	1.9 [1.4-2.6]	73 [50.5-85.2]	0	0.088	
**Country**										< 0.001
Egypt	4.1 [2.8-5.8]	NA	2	368	905	1.4	47.4	0.168	NA	
Ivory Coast	24.8 [9.1-67.4]	NA	1	44	88	NA	NA	1	NA	
Mali	13.6 [6.9-27.1]	NA	1	76	152	NA	NA	1	NA	
Niger	118.1 [13.9-1006.4]	NA	1	29	46	NA	NA	1	NA	
Nigeria	1.5 [0.7-2.9]	NA	1	64	64	NA	NA	1	NA	

	OR (95%CI)	95% Prediction interval	N Studies	N HCC cases	N controls	H (95%CI)	I² (95%CI)	P-value heterogeneity	P-value Egger test	P-value subgroup difference
Senegal	30.4 [9.6-96]	NA	1	39	53	NA	NA	1	NA	
South Africa	9.6 [5.2-17.8]	[1.6-59.3]	5	564	526	1.5 [1-2.5]	57.9 [0-84.3]	0.05	0.948	
Togo	59.1 [18.5-189.2]	NA	1	40	80	NA	NA	1	NA	
Tunisia	3.2 [1.2-8.8]	NA	1	73	70	NA	NA	1	NA	
Uganda	13.8 [3.8-50.6]	NA	1	47	50	NA	NA	1	NA	
Zambia	21.1 [4.7-94.9]	NA	1	19	40	NA	NA	1	NA	
Zimbabwe	10.3 [5.2-20.6]	NA	1	182	100	NA	NA	1	NA	
**UNSD Region**										0.001
Central Africa	12.7 [4.8-33.3]	NA	1	195	49	NA	NA	1	NA	
Eastern Africa	12.1 [6.9-21.2]	[0.3-471.7]	3	248	190	1 [1-1.9]	0 [0-73.1]	0.679	0.231	
Northern Africa	4.2 [3.1-5.7]	[2.1-8.3]	4	605	1225	1 [1-2.5]	0 [0-83.8]	0.419	0.294	
Southern Africa	9.6 [5.2-17.8]	[1.6-59.3]	5	564	526	1.5 [1-2.5]	57.9 [0-84.3]	0.05	0.948	
West Africa	18.6 [6.1-56.9]	[0.4-875]	6	292	483	3.2 [2.3-4.4]	90.1 [81.2-94.8]	0	0.364	
**Country income level**										0.032
Low-income economies	25.2 [11.5-54.8]	[1.5-408.7]	4	192	328	1.6 [1-2.7]	59.8 [0-86.6]	0.058	0.684	
Lower-middle-income economies	6.6 [3.4-12.6]	[0.7-60.6]	9	953	1570	2.3 [1.7-3.1]	81.3 [65.6-89.9]	0	0.627	
Upper-middle-income economies	9.6 [5.2-17.8]	[1.6-59.3]	5	564	526	1.5 [1-2.5]	57.9 [0-84.3]	0.05	0.948	
**Year of publication**										0.664
1975-2000	11.1 [5.5-22.3]	[1.1-114.5]	10	796	731	2.2 [1.7-3]	80 [63.9-88.9]	0	0.18	
2000-2020	9 [5-16.3]	[1.2-66.6]	9	1108	1742	2.2 [1.6-3.1]	80 [62.7-89.3]	0	0.606	
**Detection assay**										< 0.001
Direct ELISA	8.9 [6.2-12.9]	[4.5-17.6]	6	786	665	1.2 [1-2]	35.9 [0-74.4]	0.168	0.881	
Enzyme immunoassay	2.7 [1.5-4.9]	[0-1610.6]	3	432	969	2 [1.1-3.7]	76.2 [21.9-92.7]	0.015	0.275	
Radioimmunoassay	14.7 [7-31]	[1.7-128.6]	7	526	519	1.7 [1.2-2.6]	66.6 [25.6-85.1]	0.006	0.16	
Rapid Diagnostic test	23 [12-44.2]	[0.1-9519.1]	3	160	320	1.5 [1-2.9]	57.2 [0-87.8]	0.097	0.293	
**Other liver disorders**										

	OR (95%CI)	95% Prediction interval	N Studies	N HCC cases	N controls	H (95%CI)	I² (95%CI)	P-value heterogeneity	P-value Egger test	P-value subgroup difference
**Timing of exposure collection**										0.005
Prospetively	2 [1.3-3.1]	[0.1-37.3]	3	236	126	1 [1-2.4]	0 [0-82.3]	0.555	0.675	
Retrospectively	0.9 [0.6-1.3]	NA	1	195	263	NA	NA	1	NA	
**Country**										0.555
Egypt	1.8 [0.5-6.3]	NA	1	32	15	NA	NA	1	NA	
Niger	3.5 [1.2-10.6]	NA	1	29	28	NA	NA	1	NA	
Senegal	1.8 [1-3.1]	NA	1	175	83	NA	NA	1	NA	
**UNSD Region**										0.021
Central Africa	0.9 [0.6-1.3]	NA	1	195	263	NA	NA	1	NA	
Northern Africa	1.8 [0.5-6.3]	NA	1	32	15	NA	NA	1	NA	
West Africa	2 [1.3-3.3]	NA	2	204	111	1.1	11.9	0.287	NA	
**Country income level**										0.278
Low-income economies	3.5 [1.2-10.6]	NA	1	29	28	NA	NA	1	NA	
Lower-middle-income economies	1.8 [1.1-2.9]	NA	2	207	98	1	0	0.981	NA	
**Setting**										0.088
Community-based	3.5 [1.2-10.6]	NA	1	29	28	NA	NA	1	NA	
Hospital-based	1.2 [0.8-1.9]	[0-105.4]	3	402	361	1.6 [1-3]	61.5 [0-89]	0.075	0.568	
**Year of publication**										0.01
1975-2000	2 [1.3-3.3]	NA	2	204	111	1.1	11.	0.287	NA	
2000-2020	0.9 [0.6-1.3]	NA	2	227	278	1	8.1	0.297	NA	
**Detection assay**										0.021
Chemiluminescent enzyme immunoassay	1.8 [0.5-6.3]	NA	1	32	15	NA	NA	1	NA	
Direct ELISA	0.9 [0.6-1.3]	NA	1	195	263	NA	NA	1	NA	
Radioimmunoassay	2 [1.3-3.3]	NA	2	204	111	1.1	11.9	0.287	NA	
**DNA**										

	OR (95%CI)	95% Prediction interval	N Studies	N HCC cases	N controls	H (95%CI)	I² (95%CI)	P-value heterogeneity	P-value Egger test	P-value subgroup difference
**Non-hepatic diseases**										
**Sampling**										0.445
Non probabilistic	8.7 [5.8-13.2]	NA	2	348	763	1.9 [1-3.9]	71 [0-93.5]	0.063	NA	
Probabilistic	26.6 [1.6-449.3]	NA	1	148	114	NA	NA	1	NA	
**Country**										0.134
Egypt	26.6 [1.6-449.3]	NA	1	148	114	NA	NA	1	NA	
Gambia	32.3 [7.7-136.3]	NA	1	198	325	NA	NA	1	NA	
South Africa	7.8 [5.1-11.9]	NA	1	150	438	NA	NA	1	NA	
**UNSD Region**										0.134
Northern Africa	26.6 [1.6-449.3]	NA	1	148	114	NA	NA	1	NA	
Southern Africa	7.8 [5.1-11.9]	NA	1	150	438	NA	NA	1	NA	
West Africa	32.3 [7.7-136.3]	NA	1	198	325	NA	NA	1	NA	
**Country income level**										0.134
Low-income economies	32.3 [7.7-136.3]	NA	1	198	325	NA	NA	1	NA	
Lower-middle-income economies	26.6 [1.6-449.3]	NA	1	148	114	NA	NA	1	NA	
Upper-middle-income economies	7.8 [5.1-11.9]	NA	1	150	438	NA	NA	1	NA	
**HCV**										
**Anti-HCV**										
**Healthy controls**										
**Sampling**										0.918
Non probabilistic	7.8 [5.5-10.9]	[4.7-12.8]	11	1350	1440	1 [1-1.7]	8.8 [0-63.7]	0.361	0.225	
Probabilistic	8.3 [2.3-29.9]	NA	1	115	199	NA	NA	1	NA	
**Timing of exposure collection**										0.636
Prospetively	6.9 [3.8-12.6]	[3.2-15.1]	7	254	347	1 [1-1.3]	0 [0-43.2]	0.798	0.032	
Retrospectively	8.3 [5.5-12.6]	[3.2-21.3]	5	1211	1292	1.4 [1-2.3]	48.2 [0-81]	0.102	0.517	
**Country**										0.522

	OR (95%CI)	95% Prediction interval	N Studies	N HCC cases	N controls	H (95%CI)	I² (95%CI)	P-value heterogeneity	P-value Egger test	P-value subgroup difference
Egypt	9.1 [5.2-15.9]	[0.2-342.4]	3	213	195	1.4 [1-2.6]	47.7 [0-84.7]	0.148	0.976	
Gambia	6 [3.8-9.5]	[0.3-119.8]	3	581	830	1 [1-2.2]	0 [0-79]	0.61	0.504	
Nigeria	6.3 [2.1-19.1]	NA	1	41	45	NA	NA	1	NA	
Rwanda	8.9 [1.9-41.5]	NA	1	26	54	NA	NA	1	NA	
Senegal	9.2 [1.8-47.3]	NA	1	49	134	NA	NA	1	NA	
South Africa	61.5 [8.5-445.1]	NA	1	380	152	NA	NA	1	NA	
Sudan	8.3 [2.3-29.9]	NA	1	115	199	NA	NA	1	NA	
Zimbabwe	15.7 [0.9-275.3]	NA	1	60	30	NA	NA	1	NA	
**UNSD Region**										0.123
Eastern Africa	10.1 [2.6-39.3]	NA	2	86	84	1	0	0.732	NA	
Northern Africa	9 [5.4-15]	[2.9-27.5]	4	328	394	1.1 [1-2.9]	21.9 [0-88]	0.279	0.941	
Southern Africa	61.5 [8.5-445.1]	NA	1	380	152	NA	NA	1	NA	
West Africa	6.2 [4.1-9.4]	[3.2-12.1]	5	671	1009	1 [1-1.2]	0 [0-32.3]	0.873	0.16	
**Country income level**										0.074
Low-income economies	6.4 [4.2-9.7]	[3.3-12.6]	5	722	1083	1 [1-1.3]	0 [0-40.4]	0.845	0.235	
Lower-middle-income economies	8.7 [5.4-13.9]	[4.5-16.8]	6	363	404	1 [1-1.9]	0 [0-70.9]	0.499	0.873	
Upper-middle-income economies	61.5 [8.5-445.1]	NA	1	380	152	NA	NA	1	NA	
**Year of publication**										0.187
1975-2000	14.4 [5.4-38.3]	[0-8058.5]	3	455	340	1.2 [1-3.6]	26.7 [0-92.4]	0.255	0.536	
2000-2020	7.2 [5.2-10]	[4.7-10.9]	9	1010	1299	1 [1-1.5]	0 [0-56.6]	0.592	0.341	
**Detection assay**										0.33
Chemiluminescent enzyme immunoassay	45 [2.4-842.8]	NA	1	32	10	NA	NA	1	NA	
Enzyme immunoassay	10.1 [2.6-39.3]	NA	2	86	84		0	0.732	NA	
Indirect ELISA	7 [4.7-10.2]	[4.2-11.5]	7	1166	1360	1 [1-1.9]	4 [0-72]	0.396	0.066	
Radioimmunoassay	4.2 [1.5-11.8]	NA	1	33	35	NA	NA	1	NA	

	OR (95%CI)	95% Prediction interval	N Studies	N HCC cases	N controls	H (95%CI)	I² (95%CI)	P-value heterogeneity	P-value Egger test	P-value subgroup difference
Unclear/Not reported	11.6 [5.9-22.8]	NA	1	148	150	NA	NA	1	NA	
**Liver cirrhosis**										
**Timing of exposure collection**										0.014
Prospetively	0.8 [0.3-2]	NA	2	75	127	1	0	0.821	NA	
Retrospectively	3.2 [1.7-6.1]	NA	2	462	195	1	0	0.624	NA	
**Country**										0.049
Gambia	3.2 [1.7-6.1]	NA	2	462	195	1	0	0.624	NA	
Rwanda	0.6 [0.1-3.7]	NA	1	26	79	NA	NA	1	NA	
Senegal	0.8 [0.3-2.6]	NA	1	49	48	NA	NA	1	NA	
**UNSD Region**										0.175
Eastern Africa	0.6 [0.1-3.7]	NA	1	26	79	NA	NA	1	NA	
West Africa	2.3 [1.2-4.3]	[0-359.9]	3	511	243	1.5 [1-2.8]	54 [0-86.8]	0.114	0.271	
**Country income level**										0.076
Low-income economies	2.7 [1.5-4.9]	[0.1-126.8]	3	488	274	1.3 [1-2.2]	36.1 [0-79.6]	0.209	0.264	
Lower-middle-income economies	0.8 [0.3-2.6]	NA	1	49	48	NA	NA	1	NA	
**Year of publication**										0.014
1975-2000	0.8 [0.3-2]	NA	2	75	127	1 NA	0 NA	0.821	NA	
2000-2020	3.2 [1.7-6.1]	NA	2	462	195	1 NA	0 NA	0.624	NA	
**Detection assay**										0.175
Enzyme immunoassay	0.6 [0.1-3.7]	NA	1	26	79	NA	NA	1	NA	
Indirect ELISA	2.3 [1.2-4.3]	[0-359.9]	3	511	243	1.5 [1-2.8]	54 [0-86.8]	0.114	0.271	
**Non-hepatic diseases**										
**Sampling**										0.366
Non probabilistic	8.7 [5.6-13.6]	[2-37.9]	13	1513	2459	1.8 [1.4-2.4]	70.2 [47.5-83.1]	0	0.104	
Probabilistic	13.2 [6-28.9]	NA	2	330	248	2 [1-4.2]	74.4 [0-94.2]	0.048	NA	
**Timing of exposure collection**										0.898

	OR (95%CI)	95% Prediction interval	N Studies	N HCC cases	N controls	H (95%CI)	I² (95%CI)	P-value heterogeneity	P-value Egger test	P-value subgroup difference
Prospetively	9.8 [3.6-26.6]	[0.1-943.6]	4	537	620	2.8 [1.8-4.3]	87.1 [69.1-94.6]	0	0.483	
Retrospectively	9.2 [6.3-13.4]	[3.3-25.2]	11	1306	2087	1.4 [1-2]	52.3 [5.3-76]	0.021	0.186	
**Country**										0.002
Egypt	11.4 [7.7-16.8]	[0.3-518.9]	3	516	1053	1.6 [1-3]	62.1 [0-89.2]	0.071	0.895	
Ivory Coast	3.5 [1.2-10.6]	NA	1	44	88	NA	NA	1	NA	
Mali	9.4 [4-22]	NA	1	76	152	NA	NA	1	NA	
Nigeria	1.9 [0.7-5.1]	NA	1	64	64	NA	NA	1	NA	
South Africa	5.9 [3.8-9.2]	[2.3-15.5]	4	489	801	1 [1-1.7]	0 [0-64.4]	0.732	0.491	
Togo	22.9 [2.8-188.7]	NA	1	40	80	NA	NA	1	NA	
Tunisia	12.9 [5-33.6]	NA	1	73	70	NA	NA	1	NA	
Zimbabwe	28.6 [10.1-81.1]	NA	1	182	100	NA	NA	1	NA	
**UNSD Region**										0.006
Central Africa	15.2 [2-113.5]	NA	1	195	49	NA	NA	1	NA	
Eastern Africa	28.6 [10.1-81.1]	NA	1	182	100	NA	NA	1	NA	
Northern Africa	14.2 [9-22.3]	[3.2-63]	5	753	1373	1.9 [1.2-3]	70.8 [25.9-88.5]	0.008	0.812	
Southern Africa	5.9 [3.8-9.2]	[2.3-15.5]	4	489	801	1 [1-1.7]	0 [0-64.4]	0.732	0.491	
West Africa	5 [2.3-11]	[0.3-92.2]	4	224	384	1.6 [1-2.8]	63 [0-87.5]	0.044	0.981	
**Country income level**										0.207
Low-income economies	10.6 [4.8-23.5]	NA	2	116	232	1 NA	0 NA	0.44	NA	
Lower-middle-income economies	10.7 [5.8-19.5]	[1.4-81.1]	8	1043	1625	2.2 [1.6-3.1]	80 [61.2-89.7]	0	0.14	
Upper-middle-income economies	5.9 [3.8-9.2]	[2.3-15.5]	4	489	801	1 [1-1.7]	0 [0-64.4]	0.732	0.491	
**Year of publication**										0.365
1975-2000	6.1 [2-18.6]	[0.1-645.8]	4	437	379	2.2 [1.3-3.6]	79 [43.8-92.1]	0.003	0.364	
2000-2020	10.6 [7.2-15.5]	[3.3-33.9]	11	1406	2328	1.6 [1.2-2.3]	62.7 [28.5-80.6]	0.003	0.277	
**Detection assay**										0.115
Enzyme immunoassay	3.3 [1.1-10.6]	NA	2	259	113	1.8 [1-3.9]	70.1 [0-93.3]	0.068	NA	

	OR (95%CI)	95% Prediction interval	N Studies	N HCC cases	N controls	H (95%CI)	I² (95%CI)	P-value heterogeneity	P-value Egger test	P-value subgroup difference
Indirect ELISA	11.2 [7.5-16.7]	[3.2-39]	10	1424	2274	1.8 [1.3-2.5]	68.1 [38.3-83.5]	0.001	0.257	
Rapid Diagnostic test	7.3 [3.8-13.9]	[0.1-475.5]	3	160	320	1.3 [1-2.2]	36.2 [0-79.6]	0.208	0.974	
**HDV**										
**Anti-HDV**										
**Non-hepatic diseases**										
**Timing of exposure collection**										0.886
Prospectively	24.2 [4.3-135.7]	NA	2	80	101	1 NA	0 NA	0.375	NA	
Retrospectively	30.8 [1.9-509]	NA	1	195	49	NA	NA	1	NA	
**Country**										0.375
Niger	42 [5.1-346.9]	NA	1	29	46	NA	NA	1	NA	
Tunisia	8 [0.4-159]	NA	1	51	55	NA	NA	1	NA	
**UNSD Region**										0.667
Central Africa	30.8 [1.9-509]	NA	1	195	49	NA	NA	1	NA	
Northern Africa	8 [0.4-159]	NA	1	51	55	NA	NA	1	NA	
West Africa	42 [5.1-346.9]	NA	1	29	46	NA	NA	1	NA	
**Country income level**										0.375
Low-income economies	42 [5.1-346.9]	NA	1	29	46	NA	NA	1	NA	
Lower-middle-income economies	8 [0.4-159]	NA	1	51	55	NA	NA	1	NA	
**Setting**										0.53
Community-based	42 [5.1-346.9]	NA	1	29	46	NA	NA	1	NA	
Hospital-based	16.4 [2.1-126.7]	NA	2	246	104	1 NA	0 NA	0.52	NA	
**Year of publication**										0.53
1975-2000	42 [5.1-346.9]	NA	1	29	46	NA	NA	1	NA	
2000-2020	16.4 [2.1-126.7]	NA	2	246	104	1 NA	0 NA	0.52	NA	
**Detection assay**										0.53
Indirect ELISA	16.4 [2.1-126.7]	NA	2	246	104	1 NA	0 NA	0.52	NA	

	OR (95%CI)	95% Prediction interval	N Studies	N HCC cases	N controls	H (95%CI)	I² (95%CI)	P-value heterogeneity	P-value Egger test	P-value subgroup difference
Radioimmunoassay	42 [5.1-346.9]	NA	1	29	46	NA	NA	1	NA	
**HBV/HCV**										
**HBsAg/Anti-HCV**										
**Healthy controls**										
**Timing of exposure collection**										0.86
Prospetively	6 [0.3-113]	NA	1	60	30	NA	NA	1	NA	
Retrospectively	7.9 [4.5-13.9]	NA	2	528	302	1.2 NA	32.9 NA	0.222	NA	
**Country**										0.468
Egypt	7.4 [4.1-13.1]	NA	1	148	150	NA	NA	1	NA	
South Africa	43.4 [2.7-709.3]	NA	1	380	152	NA	NA	1	NA	
Zimbabwe	6 [0.3-113]	NA	1	60	30	NA	NA	1	NA	
**UNSD Region**										0.468
Eastern Africa	6 [0.3-113]	NA	1	60	30	NA	NA	1	NA	
Northern Africa	7.4 [4.1-13.1]	NA	1	148	150	NA	NA	1	NA	
Southern Africa	43.4 [2.7-709.3]	NA	1	380	152	NA	NA	1	NA	
**Country income level**										0.22
Lower-middle-income economies	7.3 [4.1-12.9]	NA	2	208	180	1 NA	0 NA	0.898	NA	
Upper-middle-income economies	43.4 [2.7-709.3]	NA	1	380	152	NA	NA	1	NA	
**Year of publication**										0.22
1975-2000	43.4 [2.7-709.3]	NA	1	380	152	NA	NA	1	NA	
2000-2020	7.3 [4.1-12.9]	NA	2	208	180	1 NA	0 NA	0.898	NA	
**Detection assay**										0.435
Indirect ELISA/Direct ELISA	7.4 [4.1-13.1]	NA	1	148	150	NA	NA	1	NA	
Radioimmunoassay	17 [2.2-128.1]	NA	2	440	182	1 NA	0 NA	0.34	NA	
