# Supplementary material for: Hepatitis B, C and D virus infections and risk of hepatocellular carcinoma in Africa: A meta-analysis including sensitivity analyses for studies comparable for confounders
Source: PLoS One. 2022 Jan 21;17(1):e0262903. doi: 10.1371/journal.pone.0262903 (PMC8782350; doi:10.1371/journal.pone.0262903)

S7 Fig. Funnel chart for publications of the association between anti-HCV in cases and apparently healthy controls and the risk of developing hepatocellular carcinoma.

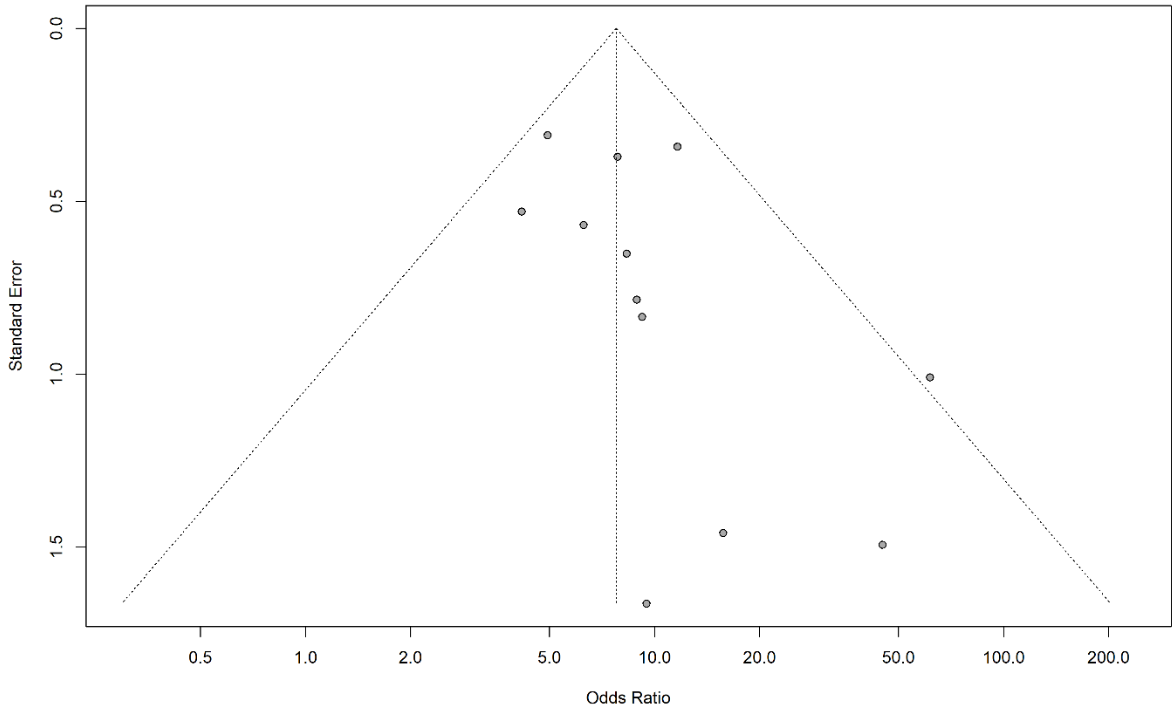

Supplement: S7 Fig — (PDF) [file pone.0262903.s018.pdf]
