# Supplementary material for: Hepatitis B, C and D virus infections and risk of hepatocellular carcinoma in Africa: A meta-analysis including sensitivity analyses for studies comparable for confounders
Source: PLoS One. 2022 Jan 21;17(1):e0262903. doi: 10.1371/journal.pone.0262903 (PMC8782350; doi:10.1371/journal.pone.0262903)

S10 Fig. Funnel chart for publications of the association between anti-HDV in cases and controls with non-hepatic diseases and the risk of developing hepatocellular carcinoma.

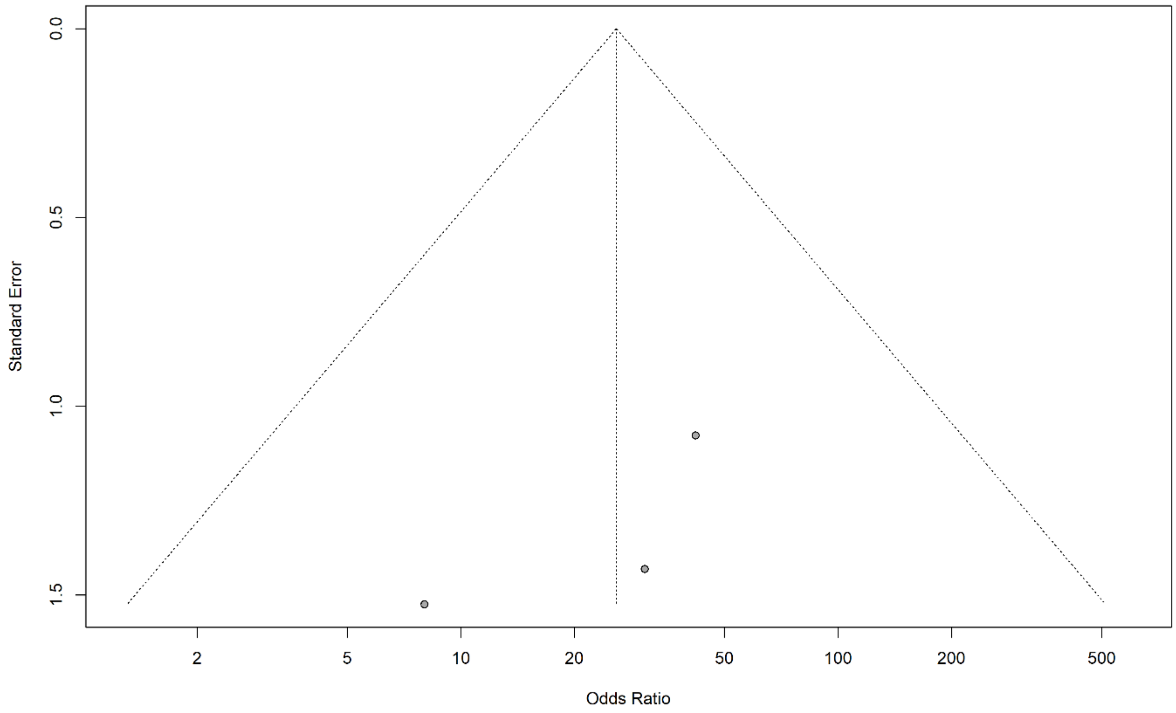

Supplement: S10 Fig — (PDF) [file pone.0262903.s021.pdf]
