# Supplementary material for: Hepatitis B, C and D virus infections and risk of hepatocellular carcinoma in Africa: A meta-analysis including sensitivity analyses for studies comparable for confounders
Source: PLoS One. 2022 Jan 21;17(1):e0262903. doi: 10.1371/journal.pone.0262903 (PMC8782350; doi:10.1371/journal.pone.0262903)
Supplement: Attachment — Submitted filename: Response to Reviewers.doc [file pone.0262903.s023.doc]

**Review Comments to the Author**

Editor

The authors tried to address reviewer's queries. However, the response was not perfect. So, authors need to fully answer the reviewer's comments.

Reviewer #1:

All comments have been addressed.

Authors: Thank you.

Reviewer #1: The analyses were performed according to the presence of HBsAg, HBeAg, HBV DNA or HCV RNA. Actually, the odds ratio of developing HCC should be performed according to the presence of chronic hepatitis B or C or the presence of LC. This study is a meta-analysis which systemically reviewed and analyzed the previously published studies; therefore, I understand the limitation. Considering the specificity of the region where studies were performed, I think it can be a valuable study.

Authors: Thank you.

Reviewer #2: Authors tried to revise their article following the reviewer’s comment, however, still there is unresolved issues.

1. If authors only included patients without any antiviral therapy, that should be declared as one of inclusion criteria.

Authors: Thanks to the Reviewer for this comment. We understand that antiviral therapy for hepatitis B, C or D may be a major factor influencing the development of hepatocellular carcinoma. We systematically collected from the included studies and presented all reported socio-demographic and clinical, quantitative and qualitative variables likely to influence the association between viral hepatitis (B, C and D) and hepatocellular carcinoma in Africa. We have carefully reviewed all of the included studies and to the best of our knowledge, the information on antiviral treatment for hepatitis is unclear and/or reported. We would have performed a subgroup analysis for studies with patients on and without viral therapy to highlight this factor as a potential source of heterogeneity in our estimates. Unfortunately, these data were not found in the included studies. We have now clarified in the main manuscript that our inclusion criteria did not depend on the antiviral therapy status to remove any potential confusion.

2. When collecting characteristics from each article, mean age, proportion of male gender, median time to HCC development should be investigated and presented in table. I can’t find those data in S8 or S9 tables.

Authors: For each included study, we systematically collected the reported mean age and standard deviation for cases and controls. This information is presented in the S9 Table. In line 2 of the S9 table, for example, for the study by Amr et al., 2014, the mean age reported is 52.2 years for cases and 45.4 years for controls. We also systematically collected the number of male subjects for cases

and controls. This information is presented in the S8 Table. In line 5 of the S8 Table, for example, for the study by Bahri et al., 2011, the number of reported males is 100 for cases and 152 for controls.

For the median time to development of HCC, this information is not clear and/or reported in the included studies. This could also be understood from the fact that all the included studies are case controls which are characterized by retrospective exposure (viral hepatitis infection) of which the exact time of occurrence is not known.
